# A strategic approach to multi-omics literature retrieval in next generation mammalian cell bioprocessing

**DOI:** 10.1038/s41540-025-00630-x

**Published:** 2025-11-28

**Authors:** Eva Price, Duygu Dikicioglu

**Affiliations:** Department of Biochemical Engineering, University College London, London, UK

**Keywords:** Biological techniques, Biotechnology, Computational biology and bioinformatics

## Abstract

Scientific literature is being published at an exponential rate, including in the field of mammalian cell bioprocessing. At the same time, the research landscape is becoming more diverse, with the emergence of multiple specialised subfields. This rise in information availability as well as broadening of research fields has a direct impact on ease of information retrieval. While this growth offers valuable insights, it also makes information retrieval more complex. Developing effective literature search queries has become increasingly challenging. This work discusses the process of literature query search refinement and the nuances of maintaining search sensitivity and specificity in the context of multi-omics research for next-generation mammalian cell bioprocessing.

## Introduction

Multi-omics refers to the combined analysis of two or more high-throughput omics disciplines including genomics, transcriptomics, proteomics or metabolomics. Combining different types of molecular data helps in understanding complex biological systems, and combining these data in a mathematically meaningful way is referred to as multi-omics integration^1,2^. In the context of bioprocessing of medicines and next-generation therapies, such methods are increasingly used to characterise cell lines and optimise bioproduction processes^3,4^.

As the field of multi-omics continues to expand, the richness of its potential is matched by the complexity of its communication. While technological advances have made it easier to generate multi-layered molecular data, the inconsistent use of terminology and metadata threatens the accessibility, discoverability, and interpretability of that work, especially in an era increasingly reliant on machine-driven research. This obstacle severely manifests itself in endeavours to analyse literature trends in multi-omics research, highlighting a critical bottleneck: the difficulty of accurately retrieving relevant literature. The challenge arises due to the complexity of search semantics, the interplay between language, keyword structure and search engine behaviour. Together, these factors can unintentionally bias or limit our understanding of the research field.

The critical assessment we will detail in the following sections identified that certain terms presumed to be blanket descriptors across the fields, such as “multi-omics” failed to capture the full scope of relevant studies. In theory, such umbrella terms should encompass a wide range of omics combinations such as “genomics and transcriptomics” or “genomics, transcriptomics and proteomics”, but in practice they often retrieved only a subset of the available literature. There were numerous multi-omics studies that were not indexed under the term at all, instead using specific combinations or alternatives such as “integrative omics” or “systems-level analysis”. Small changes in terminology impact which papers are retrieved, how many results are returned and how those results shaped the understanding of the field. This variation in keyword usage highlights a challenge in search terms that appear comprehensive, which may, in fact, be semantically narrow and therefore offer and incomplete or skewed picture of the research landscape.

In mammalian cell culture systems used in bioprocessing, multi-omics-driven insights are becoming increasingly essential for improving cell line performance, productivity, and product quality^3,5,6^. Among these systems, Human Embryonic Kidney (HEK293) cells serve as a widely used mammalian host, particularly in the production of viral vectors and recombinant proteins for cell and gene therapy applications^7–9^. Their human origin, robustness, and ease of culture make them a model of both biological and industrial relevance^10^. Prior work with publicly available HEK293 multi-omics datasets has identified challenges related to secondary data usage^11^. This review reports a related barrier and expands the investigation to mammalian bioprocessing as a broader framework, which also includes the standard antibody expression workhorse, Chinese Hamster Ovary (CHO) platform: the semantic and structural inconsistencies in how such studies are indexed, retrieved, and interpreted.

In this work, we set out to investigate how multi-omics methods are being applied to advance mammalian cell bioprocessing research with a critical examination of the impact of query structure and semantic choices on literature and information retrieval. A framework is proposed for building more consistent, logic-aware search strategies and demonstrating its application in identifying publications relevant to multi-omics approaches in mammalian cell bioprocessing. This framework was then implemented to conduct a targeted bibliometric case study on mammalian bioprocessing, offering insights into trends, blind spots, and practical recommendations for researchers navigating this interdisciplinary space.

## Results

### Development of domain-specific search term dictionaries

The primary function of the study was to investigate mammalian cell culture for bioprocessing; this began by surveying the breadth of publications in the field by exploring how different search terms affected literature retrieval related to key industrially relevant cell lines. Specifically, the focus was on mammalian cells widely used in biologics manufacturing, cell and gene therapy applications, particularly those involved in the production of viral vectors or therapeutic proteins^12^. These include African Green Monkey Kidney (Vero) cells^13^, Baby Hamster Kidney (BHK-21) cells^14^, Chinese Hamster Ovary cells^15^, Human Embryonic Kidney cells^16^, Human Embryonic Retinal (PER.C6) cells^17^, Human Fetal Lung Fibroblast (MRC-5)^18^, and Mouse Myeloma (NS0) cells^19^.

Topic search was performed in Web of Science (WoS) using both the full cell line name and commonly used abbreviation form (Table 1) in order to assess how these cell lines were represented in the literature. This overview shows that there are striking differences in the number of publications available for searches depending on the form of the search term used.

**Table. 1:** The number of publications retrieved in WoS as of July 18^th^ 2025, using “Topic Search” for both full cell line names and their commonly used abbreviation

Cell Type	Number of Publications	Abbreviation / Cell Line	Number of Publications	Difference
African Green Monkey Kidney	2106	Vero	15,002	12,896
Baby Hamster Kidney	2432	BHK-21	2778	346
Chinese Hamster Ovary	19,567	CHO	40,279	20,712
Human Embryonic Kidney	9788	HEK293	15,745	5957
Human Embryonic Retinal	8	PER.C6	83	75
Human Fetal Lung Fibroblasts	320	MRC-5	3164	2844
Mouse Myeloma	1931	NS0	262	1669

In most cases, the shorthand terms returned a higher number of results. This is largely because established cell lines, such as HEK293 and Vero, are typically cited using their standard abbreviations in the literature. These shorthand terms are standardised and widely adopted across protocols, regulatory documents and formal communications; this is especially common where using the origin name” fetal lung” may be ambiguous or sensitive^20,21^. However, relying solely on abbreviations introduced trade-offs. While they can improve recall, they can reduce search precision when a shorthand term is non-unique or has alternative meanings in other domains^22^. Scientific best practice still recommends defining full names at first use to ensure clarity, reproducibility, and broader accessibility particularly for interdisciplinary readers and indexing systems^23^. Not adhering to this practice creates inconsistencies that pose challenges for downstream applications such as text mining or database linking, and furthermore, they affect paper recall in bibliometric analysis.

Some mismatches between the full name and shorthand usage are due to semantic ambiguity. For example, the term “Mouse Myeloma” returned more results than “NS0” even though NS0 is a specific, widely used cell line. This is likely because “myeloma” is a general term frequently used in cancer research, including studies involving mouse models of multiple myeloma^24^. Furthermore, NS0 is just one derivative of the mouse myeloma lineage, and while it serves as a parental cell line in industrial applications, it is not an all-encompassing term for all related variants. Its more specific usage and inconsistent naming conventions across publications may contribute to its relatively lower retrieval count^20,25^.

The reverse retrieval pattern appears with Vero cells. The shorthand “Vero” returned just over 15,000 results compared to just more than 2000 results for “African Green Monkey Kidney”. When refined the search query by including [AND “cell*”], the output dropped to 14,508 publications, which is indicative that approximately 500 of the original records likely referred to unrelated uses of the term. Here, as per its standard use, the wildcard symbol “*” functions as a placeholder for any characters that follow the root word “cell”, allowing the query to match variations such as cell, cells, cellular, or cell line.

The issue is even more pronounced with CHO cells. While CHO returned over 40,000 records, its full name “Chinese Hamster Ovary” yielded fewer than 20,000 publications. In this case, the acronym CHO is also a chemical abbreviation for aldehyde functional groups, amongst other things, making it a common search query term in organic chemistry literature. Unlike the Vero example, refining the CHO query with “cell*” was less effective at filtering unrelated results, since aldehyde studies often involve cells that are not Chinese Hamster Ovary cells^26,27^. As a result, a more specific and context-aware query was deemed necessary when constructing accurate search dictionaries for CHO cells to avoid retrieving irrelevant literature.

Literature search on the PER.C6 cell highlighted a search query challenge associated with the variations in syntax, particularly concerning the use of hyphens, spaces, and quotation marks (Table 2). The official name of the cell line is *PER.C6*, however, in order to investigate the influence of syntactical variations on search query performance, this was deliberately altered. In Web of Science, PER C6 (unquoted) retrieved over 1,000 results, as the space is read as Boolean AND, and consequently captures many irrelevant hits. In contrast, “PER C6”, “*PER.C6”*, and “*PER-C6”* returned a smaller set of relevant studies. This underscores the importance of distinguishing between exact phrase searches or the exclusion of quotation marks in order to avoid false positives. It also highlights how punctuation, wildcards, and Boolean logic must be carefully managed in query design. The observations from this mini case study informed the next step in the analysis, which is building search strategies that are both syntax-aware and semantically precise.

**Table 2:** Effect of quotation marks on the retrieval of publications related to the PER.C6 cell line in database searches

Query 1	Number of Retrieved Outputs	Query 2	Number of Retrieved Outputs
“PER.C6”	83	PER.C6	83
“PER-C6”	83	PER-C6	83
“PERC6”	2	PERC6	2
“PER C6”	83	PER C6	1,079
“PER.C6 Cell*“	31	PER.C6 Cell*	82
“PER.C6” AND “Cell*”	82	PER.C6 AND Cell*	82

HEK293 and CHO cells represent the most prominent mammalian systems used in bioprocessing, being the most widely studied and published upon. To streamline analysis and to have a more focused perspective on the application of multi-omics in mammalian cell bioprocessing, the remainder of the analysis will focus on these two systems. Combined, they offer sufficient depth and variability in terminology to illustrate the challenges of literature retrieval, keyword sensitivity, and database indexing in a multi-omics context. CHO cells are the established gold standard in biologics production^28^. Therefore, they will serve as our benchmark system, supported by decades of optimisation, a full sequenced genome, and a wide range of open access tools, vectors, media, and protocols^29–31^. In contrast, while HEK293 cells are increasingly used for gene therapy and vaccine production, they remain less characterised from a systems biology perspective. By focusing on both cell lines, this work will highlight how to close the knowledge gap in emerging systems like HEK293, using CHO as a point of comparison, utilising an approach similar to Malm et al.^32^.

### Creating a controlled vocabulary for HEK293 and CHO cells

The literature landscape for both cell lines was accurately assessed by ensuring to start with basic search terms and then construct a robust and inclusive search query by expanding to include commonly used naming variants in a systematic way. HEK293 served as the model case for developing and validating this process. The aim was to design search strings that balance sensitivity for retrieving all relevant publications, with specificity that excludes irrelevant ones, to create a search string for robust bibliometric analysis.

**Fig. 1:**
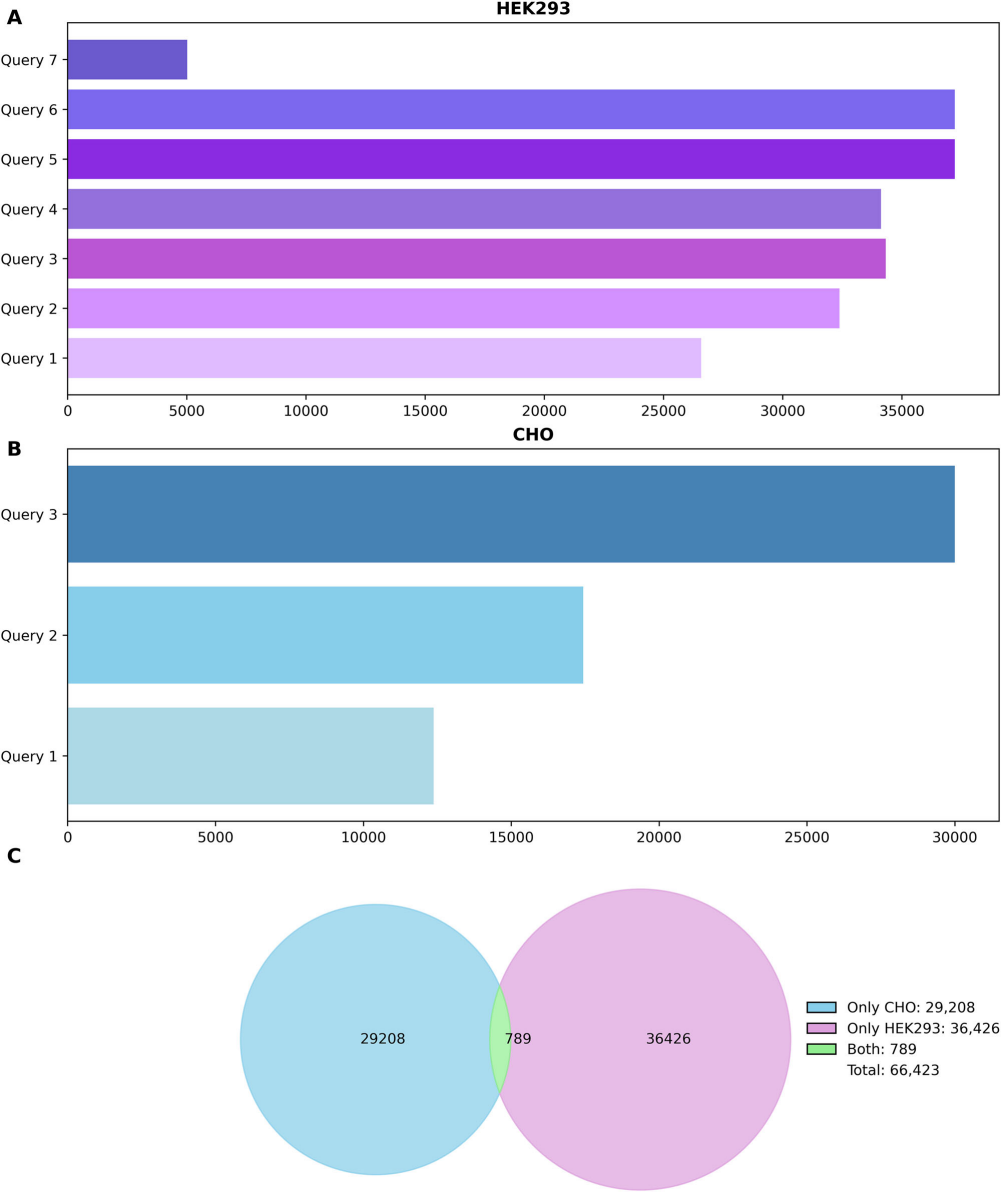
Impact of search query formulation on the number of publications retrieved for HEK293 AND CHO cells in WoS as of July 18th, 2025. **A** HEK293-related queries progressively refined the search terms by incorporating wildcards, Boolean operators and specificity constraints, resulting in a range of retrieved publications from 5016 for Query 7 to 37,215 for Query 5 & 6. **B** CHO related queries similarly expanded from a narrow formulation (“Chinese Hamster Ovary” AND “CHO” AND “cell*”) to broader combinations incorporating specific CHO line variants, increasing retrieval from 12,375 publications in Query 2, to 29,997, in Query 3. **C** Venn Diagram showing the overlap of finalised search terms (green) of 789 publications retrieved between CHO (blue) and HEK293 (purple).

HEK293-related terms occur in literature in multiple forms, including full name “Human Embryonic Kidney Cell”, abbreviated formats “HEK293” or “HEK 293” and their derivatives such as “HEK293T”, “HEK293S”. These variants were accounted for, by progressively refining the search query using combinations of exact phrases, wildcards to capture suffixes, Boolean operators to control logic and quotation marks to anchor phrases and ensure accurate matching (Fig. 1A, also see Supplementary Material Table S1). This strategy also included “Cell*” filter to ensure focussing on cellular studies. Seven search queries were developed progressively to capture HEK293-related publications in WoS. The strictest query was conducted requiring the co-occurrence of “Human Embryonic Kidney and a HEK293 variant and a cell-associated term. Each iteration reflected the trade-off between precision and recall: narrower queries risked missing more relevant studies while broader ones included unrelated content. After evaluation, Query 5 ( = ((“Human Embryonic Kidney” OR HEK293* OR “HEK 293*“) AND “cell*“)) offered the best balance of coverage and specificity, combining the name variants, wildcards, and cell specific terms effectively.

As highlighted earlier, “CHO” is a particularly ambiguous term in biological literature, frequently appearing in unrelated biological contexts such as carbohydrate chemistry, or acronyms for unrelated topics, even when combined with “cell*”. This ambiguity was observed to substantially reduce search precision if not addressed carefully.

It was necessary to adopt a stringent approach to query design to ensure that the bibliometric analysis accurately captured studies specifically related to Chinese Hamster Ovary cells. This included prioritising full and unambiguous terms, such as “Chinese Hamster Ovary” and “Chinese Hamster Ovary Cell*“, to reduce false positives and by explicitly incorporating abbreviations such as “CHO cell*“ within well-defined Boolean structures to ensure relevance, explicitly listing common CHO cell line derivatives such as CHO-K1, CHO-DG44, CHO-S, avoiding the use of wildcards, and carefully using quotation marks and parentheses to control phrase matching and logic. Given the broad usage of “CHO” across disciplines, the risk of irrelevant retrieval is higher than relatively uniquely named cell lines like HEK293. As such, the search strategy for CHO emphasised specificity over genericity, aiming to include only publications with a high likelihood of focusing on CHO-derived cell lines used in biomanufacturing, genetic engineering, and cell biology (Fig. 1B*,* also see Supplementary Material Table S2). The final refined Query 3 combined the full name, common abbreviations, and specific CHO derivatives with “cell*” to ensure biological relevance and explicit listing of derivatives to avoid wildcard ambiguity. It would be important to note that the list of CHO derivatives adopted in this search was not exhaustive.

Once the cell line search queries were finalised, we were able to capture relevant publications where each cell line was discussed in a cellular biology context, also quantifying their overlap and exclusive use in research. While both cell lines are heavily studied, co-mention in the same paper is relatively rare, occurring in only ~1.2% of the total dataset corresponding to 789 publications (Fig. 1C, see also Supplementary Table S3). This suggests that CHO and HEK293 are typically used in distinct research contexts, with limited overlap in experimental application.

### Bioprocessing terminology and search construction

For a specific analysis, commonly used bioprocessing terms linked with mammalian cells were incorporated into the search query dictionary. This list was compiled based on collective understanding of the field (Supplementary Material Table S4). While the search terms that were used aim to capture key concepts related to mammalian cell line development and bioprocessing, the query is not exhaustive. Terminology in the field is diverse, and we observed that it is frequently inconsistently applied across publications. For example, relevant studies were observed to use alternate phrasings, proprietary terms, or broader process descriptions not explicitly included in this string^33,34^. This variability in terminology creates challenges for automatic retrieval and categorisation, which are particularly relevant issues in machine learning or natural language processing (NLP) based literature mining^35^.

**Fig. 2:**
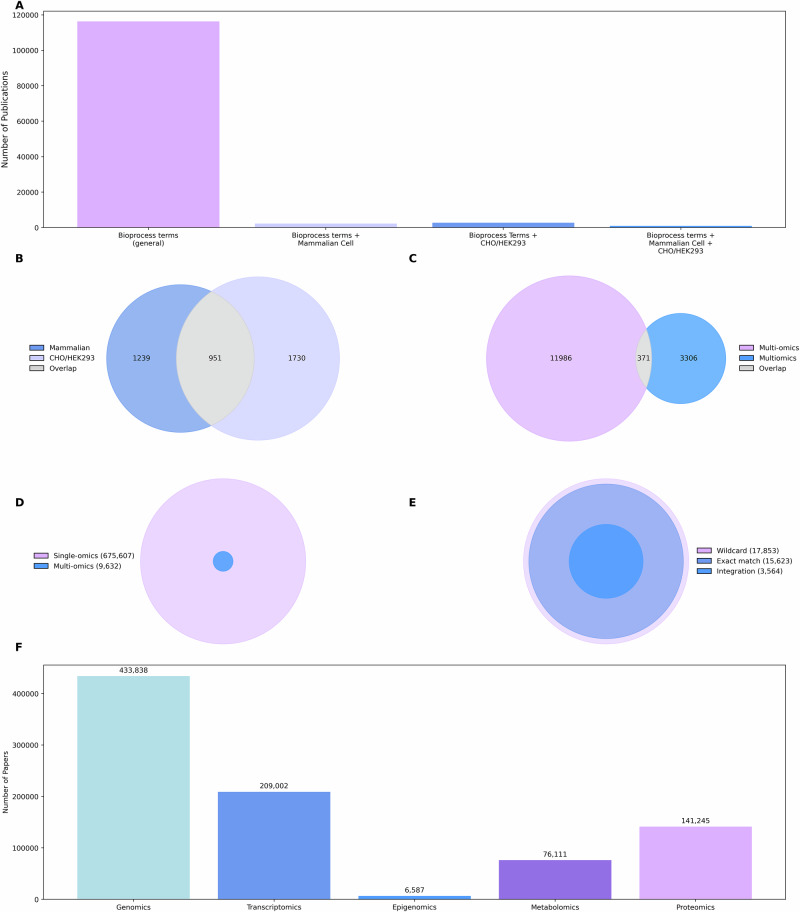
Impact of search query formulation on the number of publications retrieved in bioprocessing and omics research. **A** Number of publications retrieved using bioprocessing-related search terms in WoS. The first query (“Bioprocess terms (general)”) retrieved 116,260 publications containing at least one of a dictionary of bioprocessing-related terms without any cell-type constraint. Adding the requirement for the term “mammalian cell” reduced retrieval to 2190 publications. Restricting further to specific mammalian cell lines (CHO or HEK293) increased slightly to 2681. Restricting the search query to include both “mammalian cell* and CHO or HEK293 yielded only 951. **B** Venn Diagram showing the overlap (grey) of 951 bioprocessing publications retrieved with an additional term of “mammalian cell*” (blue) and specific annotation for CHO and HEK293 (purple). **C** Venn diagram showing the overlap between studies using the terms “multi-omics” and “multiomics”, with a total of 14,921 publications identified; areas are proportional to counts. **D** Proportion of publications mentioning at least one -omics term (purple, *n* = 675,607) versus research articles explicitly referring to multi-omics (blue, *n* = 9632); area is proportional to counts. **E** Query refinement results comparing three search strategies: exact phrase matching (light blue, *n* = 15,623), wildcard search (purple, *n* = 17,853), and wildcard search with integration (dark blue, *n* = 3564); areas are proportional to counts. **F** Literature counts by -omics domain based on optimal search queries. Bar plot showing the number of publications retrieved for each major -omics domain. Genomics has the highest representation with 433,838 publications, followed by transcriptomics (209,002), proteomics (141,421), metabolomics (76,111), and epigenomics (6587).

The number of publications retrieved from WoS from the bioprocessing search terms is 116,260, however the effect of more specific terms related to mammalian cell bioprocess greatly reduces the number of studies retrieved by about 98% (Fig. 2A). While this query offers a focused overview, it may underestimate the volume of relevant literature, for this reason, “mammalian cell*” was replaced by the joint search terms for both HEK293 and CHO, in the bioprocess search query which yielded marginally higher number of publications than before leading to more confidence in their relevance to our analysis. Combining the bioprocessing terms with “mammalian cell*” *and* search terms for *both* HEK293 and CHO resulted in less than 1000 publications returned (Fig. 2B).

### Multi-omics: search strategy development

The multi-omics search query development was the most complex step in this framework as it encompasses a wide range of scientific disciplines. Like previous approaches, an iterative method was used to build multi-omics search queries. The final curated framework can capture the combinations of omics types appearing most frequently in the literature, assess whether these were described as multi-omics, and determine whether integration of datasets was performed or mentioned.

### Multi-omics: umbrella terms and variants

Multi-omics is an umbrella term that should encompass all relevant literature for analysis; however, this is not necessarily the case as discussed earlier. This stems from various challenges. In literature, the term “multi-omics” has notable formatting variations (Fig. 2C, see also Supplementary Materials Table S5). With multi-omics yielding 11,986 results, the concatenated form “multiomics” returned fewer results, about 70% less than “multi-omics”, indicating that it is much less commonly used. When both forms were combined in a broader query (“multiomics” OR “multi-omics”), the total increased by nearly 25% of that of “multi-omics” records alone, confirming that each form of the terminology is frequently used independently across different articles. Meanwhile, 371 papers used both “multiomics” and “multi-omics” within their indexed fields. This intersection also highlights the inconsistency of terminology, not only across the field but occasionally even within a single publication, emphasising the importance of including all common variants in any comprehensive search strategy.

The literature coverage was expanded by adding related expressions such as “pan-omics,” “trans-omics,” and “cross-omics,” which are sometimes used interchangeably with “multi-omics,” especially in systems biology context (Supplementary Materials Table S6). A broader search using wildcards, “multiomic*”, and phrase variants including “multiple omics” retrieved 17,853 papers, capturing both established and emerging terms. As not all multi-omics approaches are integrative in nature, the query was further refined by including terms such as “integration,” “fusion,” and “integrative analysis.” This narrowed the results by 80%, improving specificity.

### Multi-omics: core omics domain coverage

The extent of omics research was mapped by generating a search query containing relevant single omics domains (Supplementary Materials Table S7), retrieving over 675,607 publications. Combining this query with the multi-omics search query reduced the size of the query output to approximately 1% (9632 publications) providing an estimate of the prevalence of multi-omics studies (Fig. 2D). Approximately 2.5% of these multi-omics studies are linked to single-cell omics, which is further elaborated in Supplementary Material Note S1. Considering the multi-omics query above, which returned over 17,000 records, “multi-omics” was often observed to be used without referencing specific omics types. The process of building the query for multi-omics from exact phrases to wildcards to publications on multi-omics integration (Fig. 2E) underlines the need for inclusive terminology in search strategies and highlights inconsistencies in how multi-omics is reported in the literature.

The focus of this review is centred around five standard omics technologies (Fig. 2F) for initial query generation: genomics, transcriptomics, epigenomics, proteomics and metabolomics. Other less common type of omics such as glycomics or fluxomics, relevant to mammalian cell culture were assessed through a separate expanded search query. These dictionaries were simpler given their lower frequency, relatively recent emergence, and restrictive terminology, which was in contrast to the standard omics technologies.

Recognising the limitations of blanket terms like “multi-omics,” we proceeded to explore how sequencing studies, starting with genomics, are indexed in WoS. Genomics, though well-established, was described in numerous ways across publications, necessitating a search strategy that accounts for terminological variability. Broad terms such as “sequencing” return extremely high results, over 2 million publications, but lack specificity and often retrieve unrelated content. An array of more targeted phrases was tested for refining the scope (Supplementary Material Note S2 and Table S8). Parallel strategies were developed for the retrieval of transcriptomics and epigenomics studies (Supplementary Material Table S9). For transcriptomics, “RNA-seq” was included alongside “transcriptomic*” and “transcriptom* sequencing”, as RNA-seq is widely used as both a technique and an effective label for transcriptome analysis. Many studies reference RNA-seq without explicitly using the term “transcriptomics,” so its inclusion ensured more comprehensive retrieval^36^. In contrast, genomics and epigenomics lack a single dominant technique and are studied using a broad array of methods. As such, no attempt was made to catalogue every platform-specific term, instead focusing on representative, commonly indexed keywords at the omics domain level. Metabolomics and proteomics are technologies that are inseparably mentioned along with the platforms used to generate their data. Consequently, discipline-specific and technique-based terms were combined in queries (Supplementary Material Note S3, Table S10).

### Multi-omics: pairwise and higher-order omics combinations of multi-omics

**Fig. 3:**
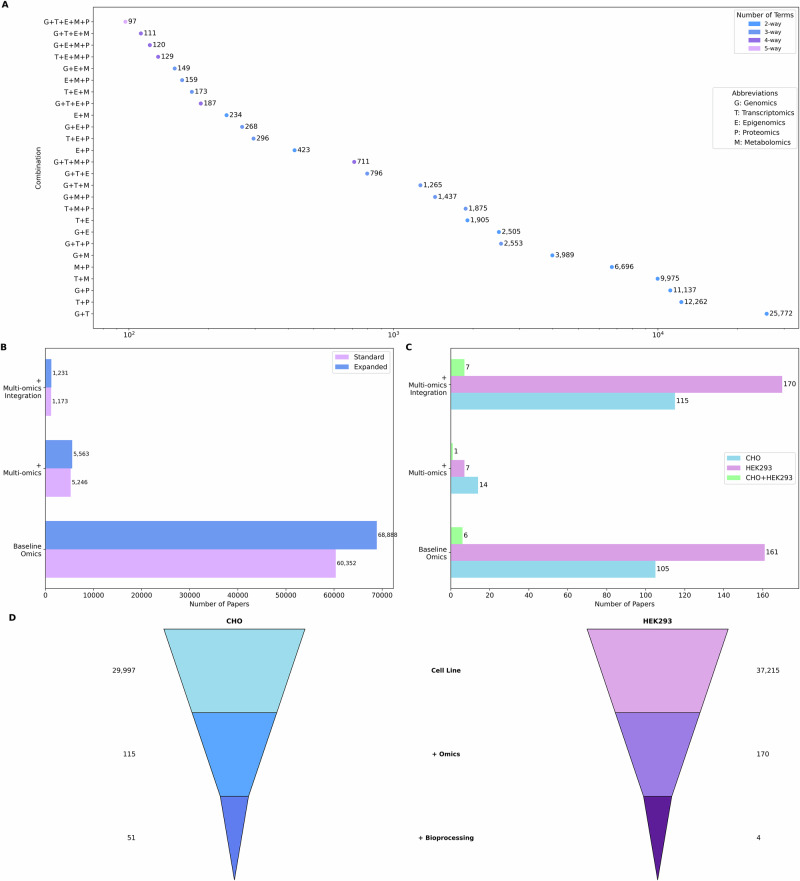
Literature landscape of multi-omics studies. **A** Dot plot of -omics domain combinations, sorted by count. Each point represents a unique combination of two or more -omics domains (Genomics [G], Transcriptomics [T], Epigenomics [E], Proteomics [P], Metabolomics [M]), with the number of publications on the abscissa. Dot colour indicates the number of terms combined (2-way to 5-way). The most frequent combinations are G + T (25,772), and T + P (12,262), and G + P (11,197) highlighting the prevalence of paired multi-omics approaches compared to higher-order combinations. Number of publications (log scale) on x-axis. **B** Horizontal bar plot comparing the number of publications retrieved using standard multi-omics search terms (purple) versus an expanded, more inclusive multi-omics term (blue). Results are shown for baseline omics-related searches, searches including explicit “multi-omics” terminology, and searches requiring both multi-omics and integration terms. The expanded multi-omics query yields consistently higher counts across all categories. **C** Comparison of the number of publications retrieved for CHO (blue), HEK293 (purple), and both (green) cell lines using expanded omics search terms, AND/OR multi-omics umbrella search queries. **D** Literature funnel plots for CHO (left-hand side) and HEK293 (right-hand side) cell lines showing the number of publications retrieved at each level of specificity of query refinement. Top section of each funnel shows the total number of papers mentioning the respective cell lines. The middle section shows the subset mentioning at least one pair of omics terms ( + omics) and the bottom section shows papers mentioning cell line and omics and bioprocessing search queries; the counts are indicated alongside each segment.

Search queries were systematically constructed to investigate the frequency of the co-occurrence of different standard omics practices in a single study. This involved an exhaustive iterative process whereby optimal search terms for each omics discipline were combined to generate queries for all possible pairings, subsequently expanding to combinations of three, four and then finally all five types of omics (Fig. 3A*,* see also Supplementary Material Table S11). The query with all five omics domains group yielded the lowest number of publications (97 publications in total). Contrary to the expectation that an increasing number of integrated omics layers would correlate with a consistent decrease in publication frequency, the data revealed a unexpected pattern based on the types of omics involved in a single study; the two-omics pairing of metabolomics and epigenomics (234 studies) was surprisingly low, as was that of epigenomics and proteomics (423 studies), yet these were dramatically overshadowed by that of transcriptomics and proteomics (25,772 studies), and even a four-omics combination of genomics, transcriptomics, metabolomics, and proteomics (711 studies). One important factor yielding this result is the combined utility and the insight that can be gained from different types of high-throughput molecular data. For example, transcriptomics and proteomic analysis are often conducted in tandem to assess to which extent the observed mRNA expression levels are reflected at the protein level and to elucidate the degree of concordance between transcription and translation.

A stepwise approach was taken to search term construction for refining our bibliometric analysis of multi-omics studies (Supplementary Material Table S12). Starting with all pairwise combinations of *at least* two omics terms occurring together in one publication, retrieved 60,352 papers. Of these, only 8.7% explicitly mentioned multi-omics or a related umbrella term, and fewer than 1% also referenced integration. This progressive narrowing, from co-occurrence to umbrella terms to integration, reduced the resulting set to 1573 studies, underscoring that many papers refer to multiple omics layers or utilise these omics technologies without conducting integrative analysis of the data. Expanding the search to include additional omics types such as fluxomics and glycomics increased the starting pool to 68,888 publications, which was subsequently reduced to 5563 and 1231 upon similar refinement (Fig. 3B). These findings reveal a semantic gap that yields a challenge in data and information retrieval; many studies conduct multi-omics research in practice, measuring and analysing two or more omics layers, but do not describe their work as such. Instead, they rely on domain-specific terms or only mention the individual omics types, leading to underrepresentation of relevant work in keyword-based queries. As a result, the term “multi-omics” is not only inconsistently applied but frequently absent, obscuring the true scope of integrative research and limiting the discoverability of such studies in automated analyses.

### Integrating cell line, bioprocessing, and multi-omics searches to streamline the final search query

The expanded dictionary of all pairwise omics combinations was then streamlined for the two specific cell lines of interest (Fig. 3C, see also Supplementary Material Table S13). A total of 105 publications were retrieved for CHO cells with the minimum pairwise omics terms. However, when this search query was expanded to explicitly include the multi-omics umbrella terms this reduced the number of publications retrieved to only 14, highlighting the discrepancy between studies conducting multi-omics and those explicitly acknowledging it. The relevant literature was comprehensively captured by employing a broader search query combining the pairwise omics search query with a disjunctive OR multi-omics umbrella search query, and this resulted in 115 publications retrieved. Similarly, for HEK293 cells, whereas only 7 studies mentioned multi-omics, the number of publications retrieved increased to 161 when that filter was removed and then to 170 with “OR” multi-omics search query. Finally, when analysing studies containing both cell lines only 1 study explicitly referenced both cell lines AND multi-omics approaches, while 6 studies were retrieved upon the omission of the multi-omics-specific terms and 7 publications for OR multi-omics specific terms.

The dataset was further refined for industrial relevance by incorporating the bioprocessing search query (Fig. 3D, see also Supplementary Material Table S14). Applying this final search strategy retrieved 51 omics studies for CHO cells, 4 for HEK293 cells, and 2 studies that explicitly referenced both cell lines in a bioprocessing context. Additional validation of the other mammalian cell lines from Table 1 supported the robustness of the final search structure (Supplementary Table S15).

### Bibliometric analysis

**Fig. 4:**
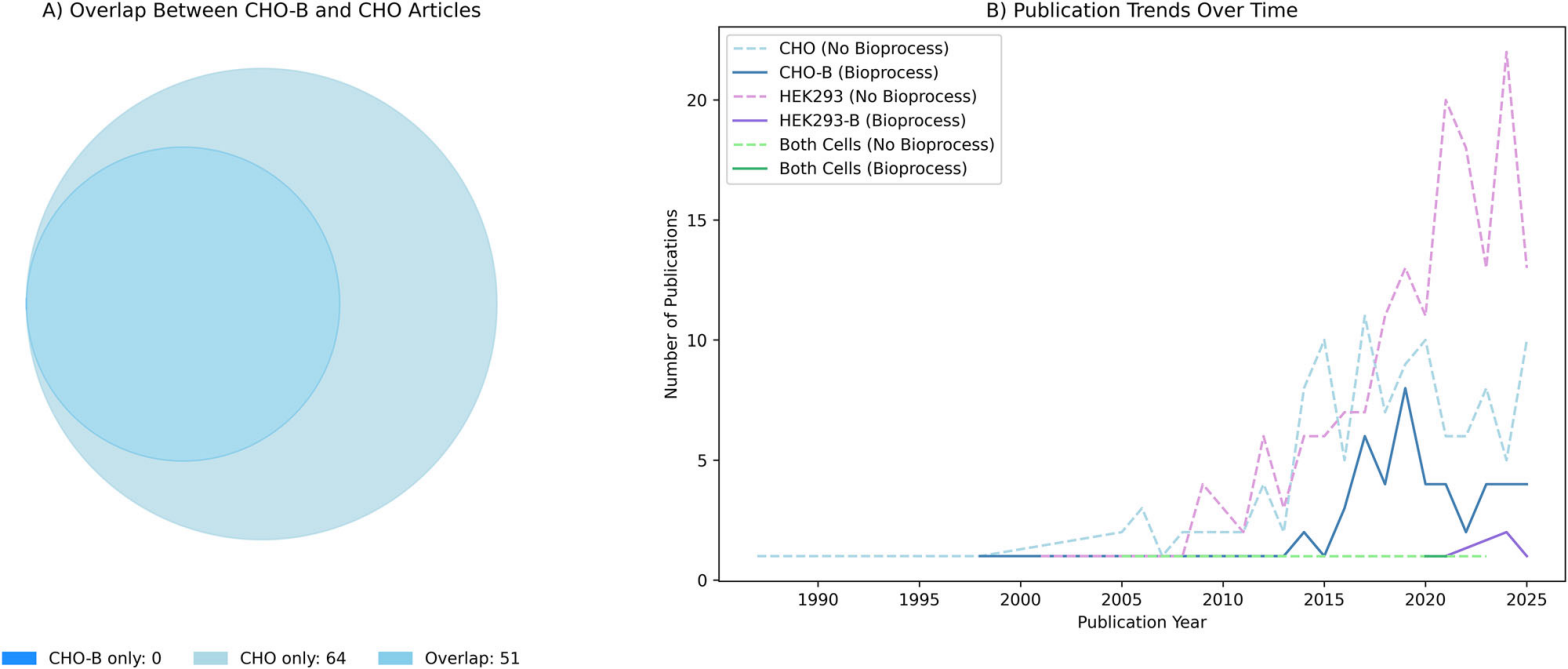
Impact of bioprocessing-specific search terms on CHO and HEK293 literature retrieval. **A** Venn diagram showing overlap between CHO studies retrieved with bioprocessing terms (CHO-B) and CHO studies without bioprocessing terms. 51 studies are shared between both searches, with 64 unique to CHO (no bioprocess) and no studies unique to CHO-B (with bioprocess). **B** Publication trends over time for CHO (blue), HEK293 (purple), and combined cell line studies (green), comparing results retrieved with and without bioprocessing terms.

The stringent search query that combined three filters including a multi-omics pairwise dictionary, a cell line dictionary, and a bioprocess dictionary yielded only a small number of publications indicating that such strict filtering might be excluding relevant studies. The trade-off between precision and recall was addressed by removing the bioprocess dictionary and allowing for broader inclusion of studies that may still provide valuable insights and consequently be relevant to bioprocessing despite not explicitly mentioning bioprocess-related terms (Table 3). Our analysis confirmed that the set of publications retrieved using the bioprocess-inclusive queries were strictly subsets of the queries that did not introduce the bioprocess filter for all cell lines (Fig. 4A).

**Table 3:** Different datasets used “-B” signifies the inclusion of the bioprocess filter

Dataset	Number of Publications	Bioprocess filter applied
CHO-B	51	Yes
CHO	115	No
HEK-B	4	Yes
HEK	170	No
CHO-HEK-B	2	Yes
CHO-HEK	7	No

The adoption of omics technologies in these two cell lines was observed to follow the timeline for the uptake and increasing utilisation of CHO and HEK293 cells; multi-omics literature for CHO cells date back to the 1990’s whereas HEK293 cells entered the research sphere in the early 2000’s with studies including both cell lines starting at around 2005. There is a notable increase in the number of publications using multi-omics and HEK293 cells around 2020, which coincides with the COVID-19 pandemic, a time when vaccine and therapeutic discovery research globally intensified for a specific target. The limited number of multi-omics studies on CHO cells that specifically focus on bioprocessing domain have been published over the course of a long term but those on HEK293 cells or those that focus on both cell lines, with bioprocessing, have only been reported since 2020. The inclusion of the bioprocessing dictionary in our search query was observed to limit the retrieval of studies on HEK293 or collectively on both cell lines the most (Fig. 4B).

**Fig. 5:**
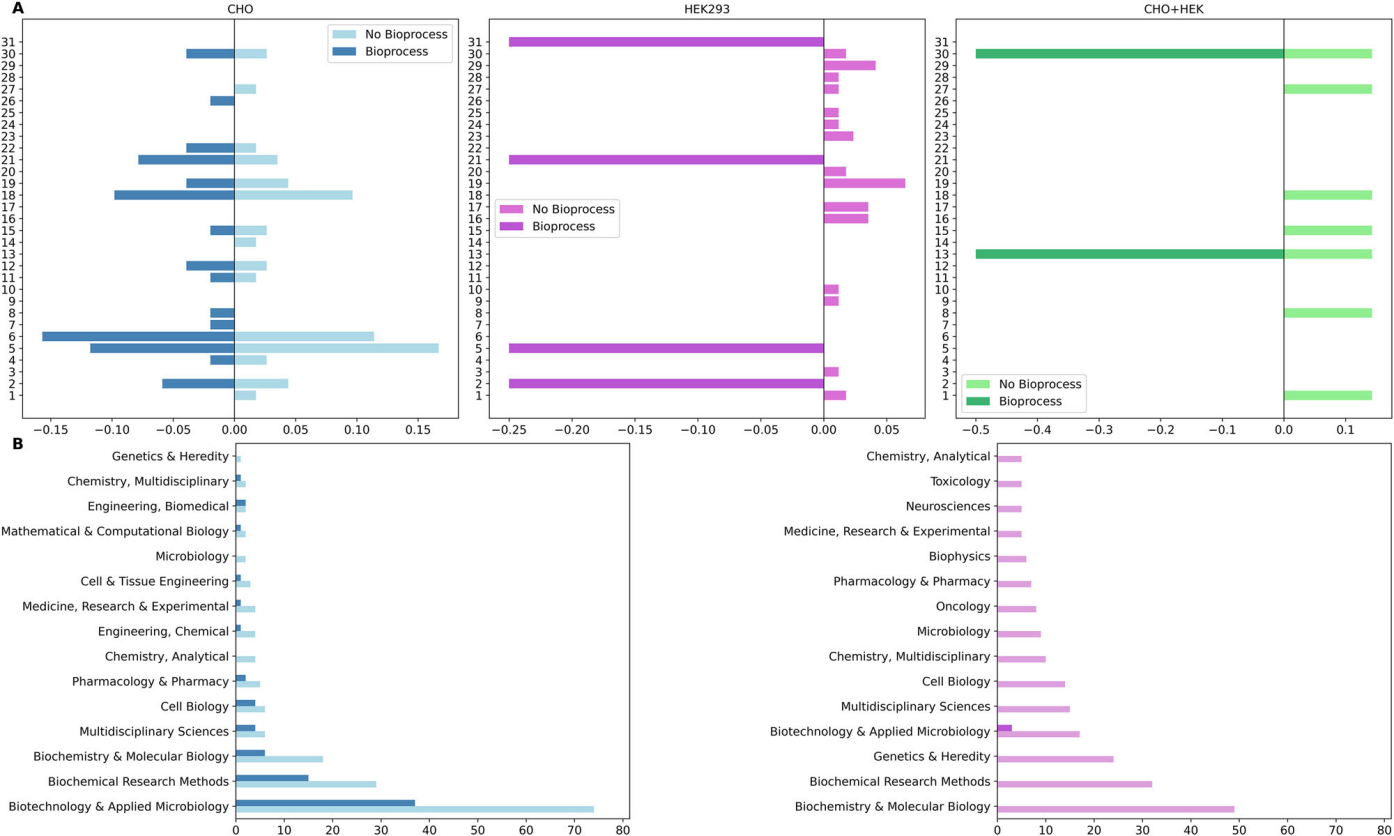
Influence of bioprocessing search filters on journal and subject category distribution in CHO and HEK293 studies. **A** Relative journal distribution for studies retrieved with and without bioprocess terms across CHO (blue), HEK293 (purple), and combined (green) cell line datasets. Each panel shows normalised frequencies of the top journals, comparing studies that include bioprocess search terms (right bars) versus those that do not (left bars). Journal frequencies were normalised by the total number of publications in each dataset to enable fair comparison across groups of different sizes. The CHO dataset shows a broader journal spread, while the HEK293 and combined cell line panels reveal narrower journal representation. This highlights how the inclusion of bioprocess filters influences not only the number of publications retrieved but also the types of journals where those studies appear. Journal names have been replaced with numerical identifiers. **B** Top WoS categories for CHO (blue) and HEK293 (purple) studies, comparing bioprocess-specific and broader multi-omics searches. CHO studies appear in both applied and diverse academic categories, with bioprocess-tagged records showing stronger alignment with Biochemical Research Methods and Biotechnology. HEK293 studies are more numerous overall but lack bioprocess-specific entries, remaining collected under general biomedical categories. Category distribution reflects both dataset size and research focus orientation.

The impact of bioprocess-related research on publication journal preferences was investigated by comparing the distribution of publications in different journals across data sets (Fig. 5A). Including bioprocess terms in search queries narrowed both the variety and the scope of journals in which these studies were published; these journals primarily focussed on featuring applied biotechnology and process-specific work.

The Web of Science categories of these journals indicated thematic differences between CHO and HEK293 studies and between bioprocessing specific and broad-scope multi-omics searches (Fig. 5B). Biotechnology & Applied Microbiology category was identified to dominate the journals publishing the multi-omics works of interest for both cell lines when the bioprocessing query applied, reflecting the central role of both these cells and the specific utility of omics technologies in the biotechnology industry. However, removing the bioprocess query results in Biochemistry & Molecular Biology being the most popular journal category for HEK293 indicating a shift towards the focus of molecular methods. For CHO multi-omics studies, the applied and industrial categories were also prominent, while broad-scope CHO multi-omics queries showed greater disciplinary diversity. In contrast, HEK293 studies were published in journals more focused on biomedical sciences. This difference in research focus suggested that CHO cells multi-omics are widely researched for an industrial focus in comparison to HEK293 multi-omics research.

Investigation of shared authorship amongst publications and affiliated countries in which the work had been conducted helps to uncover the research community and geographic regions driving the field, revealing whether bioprocess-related studies are concentrated in specific networks or more globally distributed compared to the broader field of multi-omics research. Author frequency analysis revealed distinct patterns across datasets. The analysis revealed a well-established and centralised research community in CHO multi-omics research with prominent and recurring contributions to publications across both the bioprocess-specific and the broad-scope datasets reflecting sustained leadership in the field. In contrast, the HEK293 dataset showed minimal author overlap and lower recurrence, with indications of leadership in the broad-scope multi-omics studies but not in the bioprocessing domain. The studies including both cell lines elucidated the manifestation of primarily one-off author contributions. These patterns point to a mature research network around CHO cell lines, while HEK293 and dual cell line studies are currently emergent areas.

Geographic distribution of author affiliations indicated a focalisation of the research when bioprocessing was included in the search query. In contrast, the removal of bioprocess query terms allowed the author affiliations to become geographically diverse. Our analyses showed that search terminology not only influenced the volume but also the geographic representation of retrieved literature. It was an intentional decision to avoid conventional bibliometric performance indicators such as citation impact or impact retrieval-based scores in this analysis. While these metrics are often considered as objective, it has been shown that these measurements reproduce the same visibility biases uncovered in search queries^37^. To circumvent this, the authorship and geographic analyses offer structured evidence of how search terminology shapes representation, with individual authors and countries omitted to maintain neutrality. This is an important consideration for bibliometrics and automated literature mining. The reliance on bioprocess-specific terms may bias Large Language Models and Natural Language Processing-based analyses by overrepresenting specific regions and excluding broader, geographically diverse contributions, which may distort extracted trends or knowledge graphs.

**Fig. 6:**
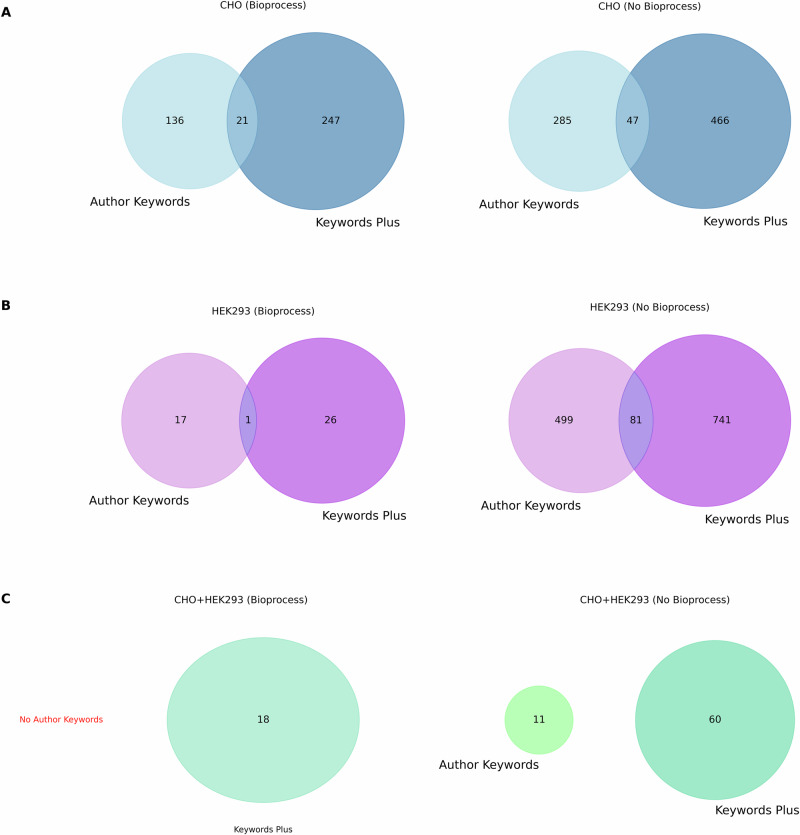
Congruency and diversity between Author Keywords and Keyword Plus keywords. This analysis is conducted within different search query contexts for bioprocess-related literature (left) and not specifically bioprocess related literature (right). All Venn diagrams are area proportional to number of keywords within the dataset but not across investigations. The left Venn in (**C**) is not proportional as there was no data for author keywords to determine proportion. **A** CHO Venn diagrams show there are more Keyword Plus keywords for CHO cells in both cases, with only a small overlap between Author keywords and Keyword Plus keywords; around 5.5% overlap was noted in the bioprocess dataset, and around 6% overlap in the dataset without bioprocess terms. **B** HEK293 Venn diagrams show that there are more Keyword Plus keywords for HEK293 cells in both cases with only a small overlap between Author and Keyword Plus keywords; around 2% overlap was observed in the bioprocess dataset, and around 6.5% overlap in the dataset without bioprocess terms. **C** Venn diagram shows that there are more Keyword Plus than Author keywords in non-bioprocess-specific literature, and there was no Author keyword for the bioprocess-specific data. No keywords were shared between Author keywords and Keywords Plus on the right clearly reflecting the divergences between how authors describe their work and how it is indexed in WoS.

The keyword metadata of retrieved publications was investigated focusing on two keyword types provided by Web of Science; Author Keywords, which are selected by the authors themselves, and Keywords Plus, which are algorithmically generated from the titles of cited references^38^. Across all cell search queries, the number of keywords returned with keyword plus was greater than those provided by the authors (Fig. 6). As expected, the publications without the bioprocessing filter demonstrate high diversity in the suggested keywords. In contrast, the bioprocess-specific subset has less diversity in keywords. The keywords plus pipeline were shown to contribute a more diverse subset of keywords than the authors, this could be linked to the fact that Keyword plus contributes 10 algorithmically generated keywords to each publication within WoS whereas the number of Author Keywords requested for journal submission varies between publishers, and therefore these two types of keywords are not proportionally comparable.

**Fig. 7:**
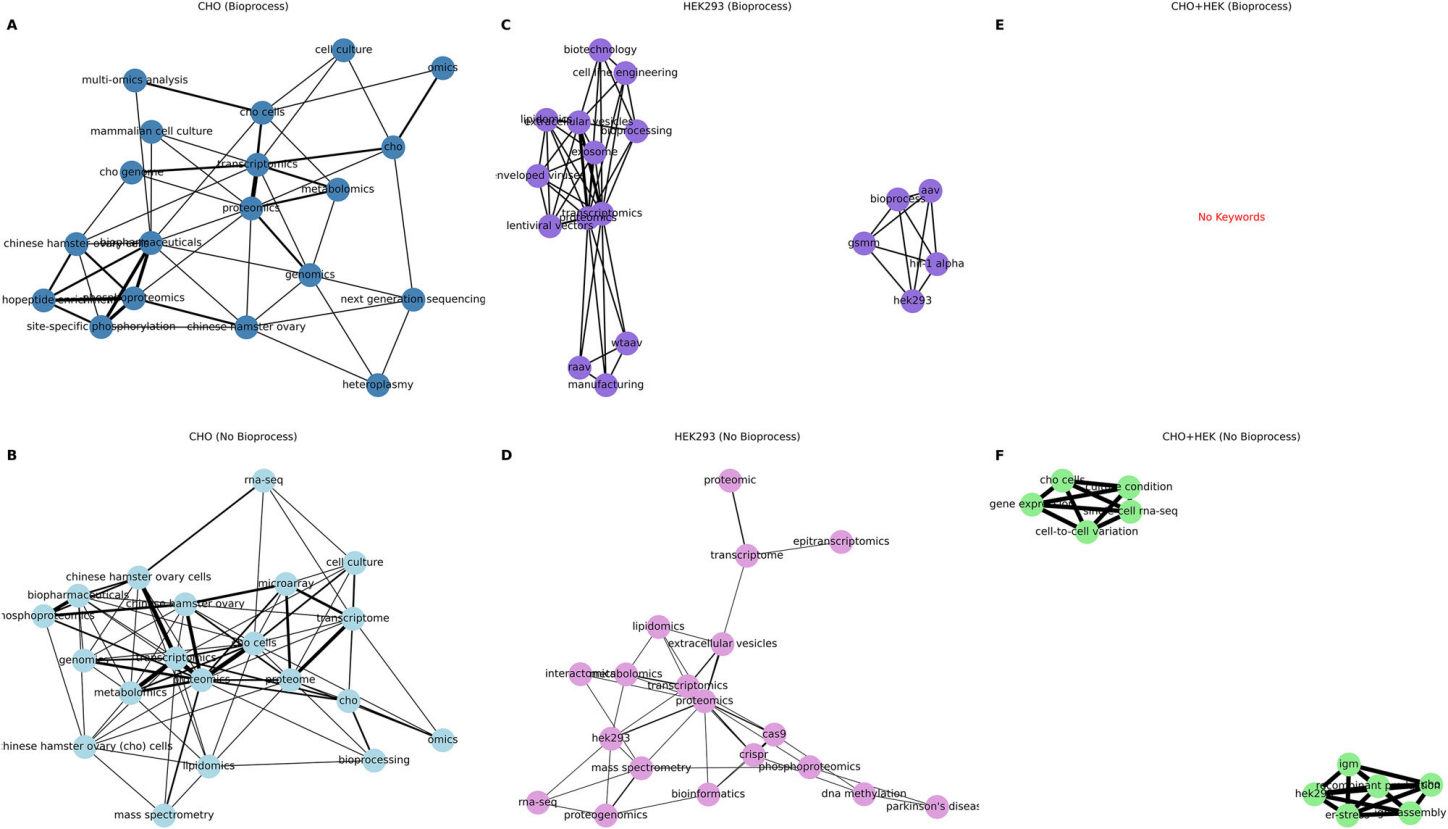
Keyword co-occurrence networks in CHO (blue), HEK293 (purple), and combined (green) cell line datasets. A co-occurrence network consists of edges and nodes where each note represents an author keyword, and the edges show how often those keywords reported to co-occur in the same publication. Thicker edges are indicative of stronger co-occurrence. The networks are represented in the following order: The co-occurrence network for CHO cell author keywords in publications retrieved using the bioprocess query (**A**) and without the bioprocess query (**B**) the co-occurrence network for HEK293 cells with the bioprocess query clearly separated into two clusters (**C**) and without the bioprocess query in a single connected network (**D**), the absence of a co-occurrence network for both cell line author keywords due to limited number of author keywords in the publications of this dataset (*n* = 2) (**E**), and the co-occurrence network for both cell line author keywords without the bioprocess query, showing two distinct networks (**F**).

Search term design not only impacted what was retrieved, but also how concepts relate to one another impacting our view on the research landscape. To understand this landscape better, co-occurrence networks were generated from the 20 most frequently occurring author keywords for all 6 datasets, visualising publication data for CHO, HEK293 and combined cell lines (Fig. 7). The studies used to construct these graphs were extracted, as indicated by their keywords, to biologically exploratory domains covering a broad range of methods and disease relevant mechanisms, then narrowed down further by the bioprocess query implementation demonstrating that terminology filters not only reduce study quantities but also restrict the conceptual research space.

The co-occurrence network for CHO-related keywords (Fig. 7A, B) were similar with most nodes representing high-throughput techniques and the cell line. These knowledge graphs are indicative that the utilisation of omics approaches for CHO cells is prevalent in bioprocessing with strong links to proteomics across both networks.

The co-occurrence graphs for HEK293 datasets contain island clusters. The keywords identified for the HEK293 dataset with bioprocess terms constitute a large distinct isolated cluster related to manufacturing, specifically of viral vectors, and another smaller cluster that also includes mention of viral vector bioprocess but also a specific protein, Hypoxia-inducible factor 1-alpha (HIF-1α), and genome-scale metabolic modelling. In the smaller cluster all of the edges appear to have equal contribution to the network indicating a balanced mention of these words, unlike the larger cluster. Once the bioprocessing query was removed, the keywords summarising the main themes of the publications were shown to be interconnected with a large cluster mostly consisting of nodes focussing on high-throughput molecular methods.

A co-occurrence network could not be constructed for the limited number of multi-omics publications where both HEK293 and CHO cells were reported in a bioprocess setting, due to limitations associated with keyword retrieval and data size. When the bioprocess dictionary was excluded, two distinct keyword co-occurrence networks could be visualised: one centralising around recombinant proteins, specifically immunoglobulin G assembly, in both HEK293 and CHO cells, and a second cluster focussing on the CHO cell variability in culture with no mention of HEK293.

### Review of the retrieved multi-omics literature of CHO and HEK293 cell lines

The refined queries defined above were used to identify the relevant literature to review. These publications included either or both cell lines, included two or more omics studies or specifically mentioned multi-omics or an umbrella multi-omics term, and were also specific to bioprocessing research (*n* = 57). Nearly half of these publications were classified as open access in WoS (*n* = 29) indicating the impact of more recent incentives towards compliance with Open Science initiatives. Unfortunately, eleven publications, ten on CHO-related research and one on both CHO and HEK293 cells, were further noted to require institutional access. This can occur due to reasons such as the nuanced definitions of open access, version control issues between different paper versions, publishers granting temporary open access, regional-specific access, and often as a result of metadata inconsistencies^39,40^.

The remaining twelve CHO publications were broadly categorised by their focus on omics technologies and data analysis, CHO cell biology, CHO cell line engineering, or cell culture and product optimisation. Lin et al.^41^ developed CHOmics a web-based platform to address the challenges of omics data for CHO cells specifically in a biopharmaceutical context. The research provided an integrative platform for processing CHO cell RNA-seq data as well as statistical modules, visualisation modules and data management for multi-omics CHO research. More recently Nguyen et al.^42^ gained insights into the mechanistic activity of S-sulfocysteine in CHO cells during culture. By leveraging metabolomics and proteomics during batch culture of CHO cells they demonstrated that S-sulfocysteine is crucial for maintaining homeostasis.

Studies exploring intrinsic CHO cell physiology delve into their fundamental biological characteristics, including clonal variations and their response to various cellular stresses that impact productivity. For example, Demirhan et al.^43^ conducted a comprehensive comparison between CHO and SP2/0 cells to identify physiological bottlenecks in CHO cells, particularly relating to protein secretion and calcium signalling through both proteomics and transcriptomics. Sulaj et al.^44^ reported the utilisation of label-free quantitative proteomics in their investigation into cellular responses to mAb expression level, meaning that all studies retrieved are not multi-omics in nature, but focussed on the transferability of information across different omics layers. This underscores a critical challenge in literature retrieval; despite targeted multi-omics search queries, such studies and similar approaches may inevitably get incorporated into the retrieval pool.

The genetic makeup of CHO cells were also investigated with the aim of identifying how their genetic components have evolved and can be leveraged for bioprocessing. A study published in 2023^45^ utilised genomics and transcriptomics to demonstrate the dynamic microevolution of extrachromosomal circular DNA in CHO cells during batch cell culture. The study identified this mechanism as a potential target for cell line optimisation as it contributes to genetic drift. Similarly, Huhn et al.^46^ investigated how chromosomal instability drives evolution towards advantageous inherited traits in CHO bioproduction lineages. They investigated this with multi-omics tools, specifically genomics and transcriptomics, and informed avenues for host cell engineering. Dahodwala et al. investigated the molecular mechanisms underlying hyper productivity in CHO cells leveraging transcriptomics, proteomics and phosphoproteomics. This work uncovered the association between increased monoclonal antibody production and the enhanced interaction of CREB1 and transgene promoter^47^.

CHO cell culture and bioprocessing optimisation strategies was another focus area of the retrieved CHO cell research. In these publications, the culture conditions were evaluated for their impact on cell performance and therapeutic product quality. CHO-K1 cell-derived exosomes were studied at the proteomic and the transcriptomic level, across different growth phases^48^. Several proteins and mRNAs were identified as key modulators of cell culture bioprocess changing over time. Zamani et al.^49^ investigated the impact of perfusion cell culture on metabolites and secreted proteins by leveraging proteomics and metabolomics. These omics data were used to demonstrate the stable extracellular environment and the metabolic profile of high cell density CHO perfusion cultures. Furthermore, it enabled the identification of signs of oxidative stress at peak densities to inform process optimisation. Global cellular adaptions in a recombinant CHO cell line were revealed by Yusufi et al.^50^. This multi-omics work leverage genomic, transcriptomic, metabolomics, lipidomic and glycomics analysis to reveal avenues for targeted mammalian cell engineering. Their analysis revealed extensive genomic rearrangements, transcriptional mechanisms as well as phenotypic adaptations in antibody-producing CHO cells. Sialylation is a key post-translational modification that has been extensively studied in CHO cells, specifically to improve the current understanding and controlling of it in cell cultures. Lewis et al.^51^ conducted a multi-omics study using metabolomics and transcriptomics at both pilot and laboratory scales to investigate the impact of oxygen limitation and identify the resulting oxygen stress as the root cause of sialylation variability in CHO bioprocessing, proposing strategies to control it. More recently, leveraging quantitative proteomics and transcriptomics, Sebastião et al.^52^ investigated the mispairing of omics signatures in CHO cells producing tri-specific antibodies, revealing cells producing misfolded protein chains. Their quantitative multi-omics approach enabled the investigation and identification of the cellular pathways, including endoplasmic reticulum (ER) stress and protein degradation that correlates with misalign level of CHO clones producing tri-specific antibodies. Clones with high mispairing showed activated ER stress, and those cells with lower mispairing have enhanced protein translation and degradation pathways, indicating a more efficient removal of misfolded proteins.

Although a smaller set of publications are available for HEK293 studies leveraging multi-omics to enhance bioprocessing than for CHO cells, this small subset of studies does provide informative insight into emergent fields. One such field is the use of HEK293 cells as platforms for viral vector and recombinant protein production. Studies leveraging multi-omics techniques aim to enhance productivity and product quality, often by characterising cellular processes and identifying engineering targets. For instance Do Minh et al.^53^ comprehensively characterised extra cellular vesicles (EVs) secreted by lentiviral-producing HEK293SF cells using proteomics, phospholipidomics and transcriptomics. This work highlighted the biophysical similarities between EVs and viral products, underscoring the need for EV characterisation in viral vaccine and vector preparations due to their co-purification and potential impact on therapeutic profiles. Similarly, Keysberg et al.^54^ employed proteomic and transcriptomic analysis to optimise small EV (sEV) production in HEK293F cells, identifying hyperthermic shifts as a strategy to increase sEV titres and linking this to the upregulation of exosome biogenesis and heat-shock related genes. Further, their work demonstrated that overexpression of single biogenesis components was sufficient to phenocopy improved sEV production.

Insights into Adeno-associated virus (AAV) production have also been advanced through multi-omics. Lin et al.^55^ conducted comparative transcriptomic and proteomic kinetic analyses of HEK293 recombinant AAV and wild-type AAV systems. Their work revealed critical viral and host cell factors for genetic and process intervention to enhance the productivity of recombinant AAV. Building on this, Zehetner et al.^56^. utilised multi-omics data, including transcriptomic, lipidomic, metabolomic, and fluxomic, to drive genome-scale metabolic models for HEK293 strains producing AAV. Their study identified significant metabolic differences between high- and low-producing strains, notably revealing that inhibiting HIF-1α in low-producing strains doubled AAV capsid productivity, despite resource allocation primarily towards cell growth rather than viral production.

The only open access research article retrieved at the intersect of multi-omics for CHO and HEK293 bioprocessing^57^ focused on HEK293 cells using CHO cells only to provide context for their work by highlighting CHO cells as the most prominent and successfully used mammalian platform, as well as emphasising the ever-increasing demand for an improved and more efficient bioproduction platform, especially one capable of generating human post-translational modifications. Similar to the apparent multi-omics studies, which presented primary data on only a single type of omics, which were discussed earlier, publications that focus on primary data generated on a single cell line utilising the other cell line for comparison or evaluation purposes do exist as exemplified by this work, requiring further vigilance in assessment of the retrieved publication sets.

This notion of prominence of the literature around CHO cells over that around HEK293 cells was clearly visible in our current assessment of the available work and the cognate review of the open-access literature. The application of multi-omics to CHO bioprocessing is comparatively mature and process-driven, routinely applied to optimise productivity, metabolism, stress responses, and product quality. In contrast, HEK293 multi-omics remains emergent and more exploratory, primarily focused on characterising platform-specific processes such as EV and viral vector production, with fewer studies translating findings into established bioprocess design or control strategies.

## Discussion

The challenges currently facing research discovery in interdisciplinary fields like multi-omics highlight an important opportunity to improve how science is described, indexed, and retrieved. We demonstrate here extensive semantic inconsistencies in the selection and use of terminology, implicating that literature indexing and retrieval are driven less by the presence of multi-omics data than by the language used to describe it. Search strategies that appear comprehensive may, in fact, be semantically narrow, skewing perceptions of the scale, maturity, and focus of a field.

Such semantic inconsistencies are not merely a bibliometric concern; they pose foundational challenges for natural language processing systems, including large language models. These models depend heavily on structured and well-labelled data to accurately learn domain concepts. When multi-omics studies are described inconsistently or without standardised terminology, even advanced systems may fail to retrieve or represent them correctly. This risks distorting downstream outputs such as topic models, literature reviews, or knowledge graphs. With natural language processing becoming increasingly integral to scientific analysis, the lack of terminological clarity emerges as an important bottleneck, not just for humans, but for machines as well. Addressing this gap will require more rigorous ontologies, community standards, and metadata practices^58^.

With these nuances in mind, it may be worthwhile to consider a coordinated framework for article keyword standardisation at the journal level to improve findability for both human and machines. Policies may require a fixed number of structured keywords organised by dimensions such as domain, technology, application, or mandate that authors select keywords from the abstract to enhance machine interoperability. Standardising the way article keyword metadata is structured and presented is a complex task, but one that warrants consideration as it could substantially enhance the visibility and retrieval of research.

While machine learning and NLP methods offer powerful tools for text mining and synthesis, they are still not entirely reliable^59–62^ Our findings emphasise that retrieval accuracy, especially in interdisciplinary domains such as multi-omics, depends critically on human domain knowledge and semantic intuition. Although presented here conceptually, this framework defines a human-in the-loop literature retrieval workflow with an algorithmic structure that embeds expert reasoning directly into the query refinement process. It consists of iterative stages: (1) the researcher defines and structures the query space by outlining key focal areas with keywords; (2) automated tools expand this space by identifying synonyms and related terms; (3) the researcher reviews and prunes the expanded space to remove misleading or unnecessary terms; (4) the refined vocabulary is synthesised into structured queries; (5) retrieval results are assessed for coverage and precision guiding subsequent iterations. Each stage performs a logical operation on the search space alternating between human interpretation and machine expansion, and could be developed as a modular function within information retrieval systems. This human-in-the-loop approach combines human insight with machine scalability providing practical groundwork for adaptive semantically aware search pipelines.

The disconnect between how researchers semantically describe their work and how algorithms interpret it emphasises the need to develop smarter, more flexible bibliometric tools. While often treated as objective indicators of disciplinary focus, WoS categories are shaped indirectly by the structure and semantics of search queries, particularly when these rely on domain-specific terminology. This underscores the need for critical engagement with category-based filtering and evaluation methods. Whether used to benchmark research areas, train classification models, or guide strategic literature reviews, these categories can reflect indexing biases introduced at the point of query design. As such, researchers and tool developers should approach WoS metadata as interpretive artifacts that require context-aware use. By embracing advances in natural language processing and fostering clearer, more consistent annotation practices, the research community can turn these challenges into a catalyst for building more inclusive, transparent, and machine-compatible pipelines, ensuring that relevant work is not only visible but also accessible and impactful.

We here demonstrate the value of well-constructed semantic queries and carefully curated multi-omics terminology in retrieving meaningful literature for bioprocess research. Despite the challenges posed by inconsistent indexing and terminology across databases, a refined approach, as proposed in this work, enabled the identification of studies that reflect the growing impact of multi-omics in mammalian cell biomanufacturing. While CHO cell research was identified as more mature, with established applications of multi-omics to optimise yield, quality, and process understanding than HEK293 applications, HEK293-based studies highlight exciting emerging opportunities in the utilisation of multi-omics in areas such as viral vector and extracellular vesicle production.

The growing application of multi-omics approaches reflects a broader shift in the field towards data-driven, systems-level bioprocess optimisation. This trajectory is promising as methodologies mature and datasets expand, both CHO and HEK293 platforms stand to benefit from increasingly sophisticated modelling, predictive control, and targeted engineering strategies. Continued efforts to harmonise terminology and improve data accessibility will further empower the research community to fully realise the potential of multi-omics in driving innovation, efficiency, and quality in biopharmaceutical production.

## Methods

### Materials

The study utilised a Dell Precision 7920 Tower workstation with dual Intel® Xeon® Gold 5217 processors, 64 GB DDR4 ECC RAM, and an NVIDIA® RTX™ A4500 graphics card. Operating on Windows 10 Pro for Workstations with a Windows 11 Pro license, it featured PCIe NVMe SSDs and SATA HDDs for storage within the Precision 7920 Tower Chassis, facilitating comprehensive scientific analyses and data processing. For reproducibility, data analysis and literature mining were performed using Python in Jupyter Notebook. A requirements.txt file with all packages and versions is openly accessible on UCL data repository (DOI: 10.5522/04/29652422).

### Literature database selection

The Web of Science (WoS) Core Collection was selected as the primary database for literature retrieval due to its broad interdisciplinary coverage, crucial for capturing research at the intersection of mammalian cell culture, multi-omics and bioprocessing as well as citation analysis capabilities^63,64^.

WoS offers wider disciplinary breadth than PubMed, which is focused primarily on biomedical literature. Bioprocessing, however, spans wider fields including biology, biotechnology, and chemical engineering, for which WoS provides stronger coverage of applied engineering-focused journals.

Scopus is a strong alternative to WoS and has several complementary strengths^65,66^. WoS was selected as the primary database for its deeper historical indexing and targeted coverage of bioprocessing disciplines. To reduce bias and reinforce the robustness of the search strategy Scopus and PubMed were utilised to validate the coverage of key terms and to check for potentially overlooked studies (Supplementary Materials Note S4 and Table S16)^67,68^. Previous studies have reported WoS to be more reliable than Scopus in terms of document type classification and indexing accuracy, making it a suitable foundation for the bibliometric analysis conducted in this work^69,70^.

All final versions of the retrievals were conducted on the 18^th^ July 2025 as the nearest possible timeframe to yield an up-to-date analysis.

### Development of domain-specific search term dictionaries

Three thematic dictionaries were created using Python, each representing a major conceptual axis of the review, and are discussed in detail above:

A. Mammalian Cell Lines

B. Industrial Bioprocessing and Cell Culture Terms

C. Multi-Omics Approaches

### Iterative term refinement in WoS

The dictionaries were refined through iterative testing within the WoS database. Terms were assessed both individually and in combination to optimise specificity and sensitivity. Searches were limited to Topic Searches (TS), which include titles, abstracts, author keywords, and Keywords Plus, to ensure relevance. Only English-language publications classified as “articles” were included.

### Combinatorial search strategy

Search terms were then combined across thematic domains to assess intersectional coverage, allowing for the targeted identification of papers at the convergence of cell line usage (A), multi-omics methodology (B), and bioprocessing applications (C). Combination strategy overview:

- Pairwise combinations: (A + B), (B + C), (A + C)
- Complete combination: (A + B + C)

### Search execution and data export

Finalised queries were executed in WoS, and resulting full record of bibliographic metadata was exported to worksheets and converted to CSV format for bibliometric analysis.

### Within domain validation

To evaluate the generalisability of the search strategy within the mammalian cell culture domain, the final search query structure was expanded to include the additional cell lines listed in Table 1. This within-domain validation was conducted on the 14^th^ of October 2025 and assessed whether the refined query logic was effective beyond CHO and HEK239 cell lines.

### Cross-database validation

To assess the robustness and completeness of search strategies, equivalent queries were tested in alternative databases, including Scopus and PubMed. All final versions of the retrievals were conducted on the 18^th^ July 2025 as the nearest possible timeframe to yield an up-to-date analysis and to ensure comparability with WoS results.

### Bibliometric analysis

The dataset of retrieved publications was analysed using bibliometric techniques, including:

- Keyword frequency analysis^71^
- Co-occurrence network mapping^72^
- Temporal trend analysis^73^

### Gap identification

By applying a refined and logic-aware search strategy across multiple databases, the cell lines, omics combinations, and bioprocessing contexts that were well-represented, and those where significant underrepresentation or absences were investigated to elucidate the gaps in the current research landscape.

## Supplementary information

Supplementary Information

## Data Availability

All data is contained within the manuscript and supplementary files. The full search and analysis pipeline is documented in the accompanying Jupyter notebook, available on UCL data repository with 10.5522/04/29652422.

## Code availability

The full search and analysis pipeline is documented in the accompanying Jupyter notebook, available on UCL data repository with 10.5522/04/29652422.
