# Supplementary material for: A strategic approach to multi-omics literature retrieval in next generation mammalian cell bioprocessing
Source: NPJ Syst Biol Appl. 2025 Nov 28;12:6. doi: 10.1038/s41540-025-00630-x (PMC12774885; doi:10.1038/s41540-025-00630-x)
Supplement: Supplementary file 1 — Supplementary Information [file 41540_2025_630_MOESM1_ESM.pdf]

Supplementary Material

Harnessing the Full Potential of Multi-omics: A Strategic Approach to Literature Retrieval in Next Generation Mammalian Cell Bioprocessing.

Eva Price and Duygu Dikicioglu*

Department of Biochemical Engineering, University College London, Gower Street, London, WC1E 6BT, UK

*Corresponding Author: d.dikicioglu@ucl.ac.uk

Table S1 Variations in search query structure and their impact on the number of retrieved publications related to HEK293 cell lines.

**Search term**		**Number of Publications**
1	TS=("Human Embryonic Kidney Cell*" OR "HEK293" OR "HEK 293")	26,576
2	TS=("Human Embryonic Kidney Cell*" OR “HEK293*” OR “HEK 293”)	32,373
3	TS=("Human Embryonic Kidney Cell*" OR “HEK293*" OR "HEK 293*")	34,315
4	TS=("Human Embryonic Kidney" OR HEK293* OR HEK 293* AND “Cell”*)	34,118
5	TS=(("Human Embryonic Kidney" OR HEK293* OR "HEK 293*") AND "cell*")	37,215
6	TS=((("Human Embryonic Kidney" AND (HEK293* OR "HEK 293*")) OR "Human Embryonic Kidney" OR HEK293* OR "HEK 293*") AND "cell*")	37,215
7	TS=("Human Embryonic Kidney" AND (HEK293* OR "HEK 293*") AND Cell*)	5,016

Table S2 Impact of search query formulation on the number of publications retrieved for Chinese Hamster Ovary (CHO) cell lines.

**Search term**		**Number of Publications**
1	TS=("Chinese Hamster Ovary" AND ("CHO*") AND "cell*")	12,375
2	TS=("CHO cell*")	17,430
3	TS=(("Chinese Hamster Ovary" OR "CHO cell*" OR "CHO-K1" OR "CHO-S" OR "CHO-DG44" OR "CHO-DUK" OR "CHOZN" OR "CHO-T" OR "CHO-Lec*" OR "CHO-H" OR "CHO-AT3-2" OR "CHO-C" OR "CHO-M" OR "CHO-AI") AND "cell*")	29,997

Table S3 Comparison of publication counts based on inclusion of CHO and HEK293 cell lines using Boolean logic in Web of Science.

**Category**	**Search Term**	**Number of Publications**
CHO only	TS=((("Chinese Hamster Ovary" OR "CHO cell*" OR "CHO-K1" OR "CHO-S" OR "CHO-DG44" OR "CHO-DUK" OR "CHOZN" OR "CHO-T" OR "CHO-Lec*" OR "CHO-H" OR "CHO-AT3-2" OR "CHO-C" OR "CHO-M" OR "CHO-AI") AND "cell*") NOT (("Human Embryonic Kidney" OR HEK293* OR "HEK 293*") AND "cell*"))	29,208
HEK293 only	TS=((("Human Embryonic Kidney" OR HEK293* OR "HEK 293*") AND "cell*") NOT (("Chinese Hamster Ovary" OR "CHO cell*" OR "CHO-K1" OR "CHO-S" OR "CHO-DG44" OR "CHO-DUK" OR "CHOZN" OR "CHO-T" OR "CHO-Lec*" OR "CHO-H" OR "CHO-AT3-2" OR "CHO-C" OR "CHO-M" OR "CHO-AI") AND "cell*"))	36,426

Both CHO & HEK293	TS=((("Chinese Hamster Ovary" OR "CHO cell*" OR "CHO-K1" OR "CHO-S" OR "CHO-DG44" OR "CHO-DUK" OR "CHOZN" OR "CHO-T" OR "CHO-Lec*" OR "CHO-H" OR "CHO-AT3-2" OR "CHO-C" OR "CHO-M" OR "CHO-AI") AND "cell*") AND (("Human Embryonic Kidney" OR HEK293* OR "HEK 293*") AND "cell*"))	789
CHO or HEK	TS=((("Chinese Hamster Ovary" OR "CHO cell*" OR "CHO-K1" OR "CHO-S" OR "CHO-DG44" OR "CHO-DUK" OR "CHOZN" OR "CHO-T" OR "CHO-Lec*" OR "CHO-H" OR "CHO-AT3-2" OR "CHO-C" OR "CHO-M" OR "CHO-AI") AND "cell*") OR (("Human Embryonic Kidney" OR HEK293* OR "HEK 293*") AND "cell*"))	66,423

Table S4 Showing the search query dictionary of bioprocessing related terms and how the use of mammalian cell specifically, or specific mammalian cell lines affect publication retrieval

Search term	Number of Publications
TS=((("mammalian cell*") AND ("producer cell line" OR "immortalized cell line" OR "immortalised cell line" OR "stable cell line" OR "cell line development" OR "cell line engineering" OR "suspension culture" OR "adherent culture" OR "defined media" OR "serum-free media" OR "fed-batch" OR "semi-perfusion" OR "perfusion culture" OR "media optimization" OR "media optimisation" OR "media components" OR "bioprocess" OR "bioreactor" OR "upstream processing" OR "downstream processing" OR "process scale-up" OR "scale-up" OR "continuous bioprocessing")))	2,190
TS=("producer cell line" OR "immortalized cell line" OR "immortalised cell line" OR "stable cell line" OR "cell line development" OR "cell line engineering" OR "suspension culture" OR "adherent culture" OR "defined media" OR "serum-free media" OR "fed-batch" OR "semi-perfusion" OR "perfusion culture" OR "media optimization" OR "media optimisation" OR "media components" OR "bioprocess" OR "bioreactor" OR "upstream processing" OR "downstream processing" OR "process scale-up" OR "scale-up" OR "continuous bioprocessing")	116,260
TS=(("producer cell line" OR "immortalized cell line" OR "immortalised cell line" OR "stable cell line" OR "cell line development" OR "cell line engineering" OR "suspension culture" OR "adherent culture" OR "defined media" OR "serum-free media" OR "fed-batch" OR "semi-perfusion" OR "perfusion culture" OR "media optimization" OR "media optimisation" OR "media components" OR "bioprocess" OR "bioreactor" OR "upstream processing" OR "downstream processing" OR "process scale-up" OR "scale-up" OR "continuous bioprocessing") AND (("Chinese Hamster Ovary" OR "CHO cell*" OR "CHO-K1" OR "CHO-S" OR "CHO-DG44" OR "CHO-DUK" OR "CHOZN" OR "CHO-T" OR "CHO-Lec*" OR "CHO-H" OR "CHO-AT3-2" OR "CHO-C" OR "CHO-M" OR "CHO-AI") AND	2,681

"cell*"OR ("Human Embryonic Kidney" OR HEK293* OR "HEK 293*")AND "cell*"))	
TS=("mammalian cell*" AND (("Chinese Hamster Ovary" OR "CHO cell*" OR "CHO-K1" OR "CHO-S" OR "CHO-DG44" OR "CHO-DUK" OR "CHOZN" OR "CHO-T" OR "CHO-Lec*" OR "CHO-H" OR "CHO-AT3-2" OR "CHO-C" OR "CHO-M" OR "CHO-AI") AND "cell*" OR ("Human Embryonic Kidney" OR "HEK293*" OR "HEK 293*") AND "cell*") AND ("producer cell line" OR "immortalized cell line" OR "immortalised cell line" OR "stable cell line" OR "cell line development" OR "cell line engineering" OR "suspension culture" OR "adherent culture" OR "defined media" OR "serum-free media" OR "fed-batch" OR "semi-perfusion" OR "perfusion culture" OR "media optimization" OR "media optimisation" OR "media components" OR "bioprocess" OR "bioreactor" OR "upstream processing" OR "downstream processing" OR "process scale-up" OR "scale-up" OR "continuous bioprocessing"))	951

Table S5 Understanding Multi-omics search queries

**Search term**	**Number of Publications**
“multi-omics”	11,986
“multiomics”	3,306
(“multiomics” OR "multi-omics")	14,921
("multiomics" AND "multi-omics")	371

Table S6 The umbrella term query development

**Search term**	**Number of Publications**
TS=("multiomics" OR "multi-omics" OR “cross-omics” or “cross omics” OR "panomics "OR "pan-omics" OR "transomics" OR "trans-omics" OR “multiple omics”)	15,623
TS=("multiomic*" OR "multi-omic*" OR "cross-omic*" OR "cross omic*" OR "panomic*" OR "pan-omic*" OR "transomic*" OR "trans-omic*" OR "multiple omic*")	17,853
TS=(("multiomic*" OR "multi-omic*" OR "cross-omic*" OR "cross omic*" OR "panomic*" OR "pan-omic*" OR "transomic*" OR "trans-omic*" OR "multiple omic*")AND (integration OR fusion OR "integrative analysis"))	3,564

Table S7 Multi-omic Query expansion

**Search term**	**Number of Publications**
TS=(“genomic*” OR “transcriptomic*” OR “proteomic*” OR “phosphoproteomic*” OR “epigenomic*” OR “epitranscriptomic*” OR “metabolomic*” OR“lipidomic*” OR “glycomic*” OR “pharmacogenomic*” OR “fluxomic*” OR “interactomic*”)	675,607

TS=(("singlecell" OR "single-cell")AND (“genomic*” OR “transcriptomic*”OR “proteomic*” OR “phosphoproteomic*” OR “epigenomic*” OR “epitranscriptomic*” OR “metabolomic*” OR “lipidomic*” OR “glycomic*” OR “pharmacogenomic*” OR “fluxomic*” OR “interactomic*”))	16,706
TS=(("multiomic*" OR "multi-omic*" OR "cross-omic*" OR "cross omic*" OR "panomic*" OR "pan-omic*" OR "transomic*" OR "trans-omic*" OR "multiple omic*") AND("genomic*" OR "transcriptomic*" OR "proteomic*" OR "phosphoproteomic*" OR "epigenomic*" OR "epitranscriptomic*" OR "metabolomic*" OR "lipidomic*" OR "glycomic*" OR "pharmacogenomic*" OR "fluxomic*" OR "interactomic*"))	9,632

Supplementary Note S1

Single-cell omics is a subcategory of omics data that enables the study of various type of omics including genomics, epigenomics and transcriptomics at an individual single cell level (*Figure S1)*. While traditional omics have been around for over 50 years with the first paper in Web of Science (WoS) published in 1941, according to the first search term in supplementary table S7, the technology to conduct high-throughput omics at single cell level has only recently been developed. In 2009 single-cell RNA sequencing was developed by Tang, et al. [1] and later in 2013 it was awarded method of the year by Nature Methods [2]. Since then, numerous technologies for single-cell omics have been developed for characterising the different molecular levels of a single cell [3,4]. Although single-cell methods take up less than 2.5% of all omics studies in WoS according to the search query, it is a field of research that it steadily increasing (Figure S2). The single-cell omics market is estimated to grow on average by 15% per year between 2024 and 2031 [5].

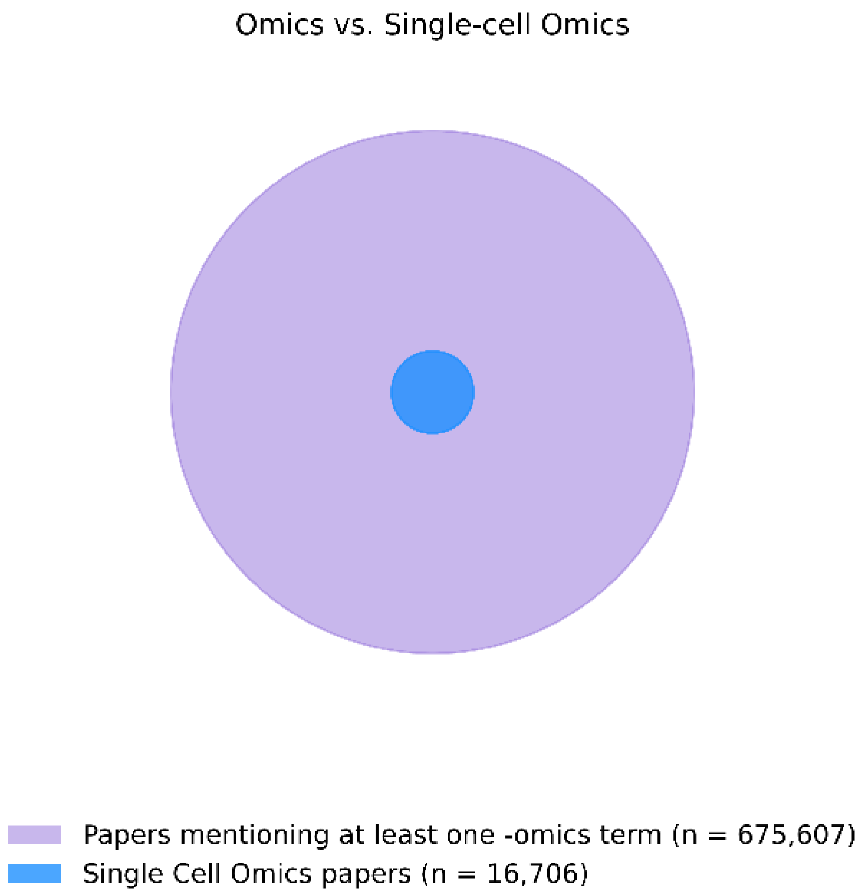

Figure S1 Proportional-area nested circle plot comparing the number of scientific papers mentioning at least one -omics term (outer circle, n=675,607)versus papers specifically focused on single-cell omics (inner circle, n=16,706). The area of each circle is proportional to the respective count of papers.

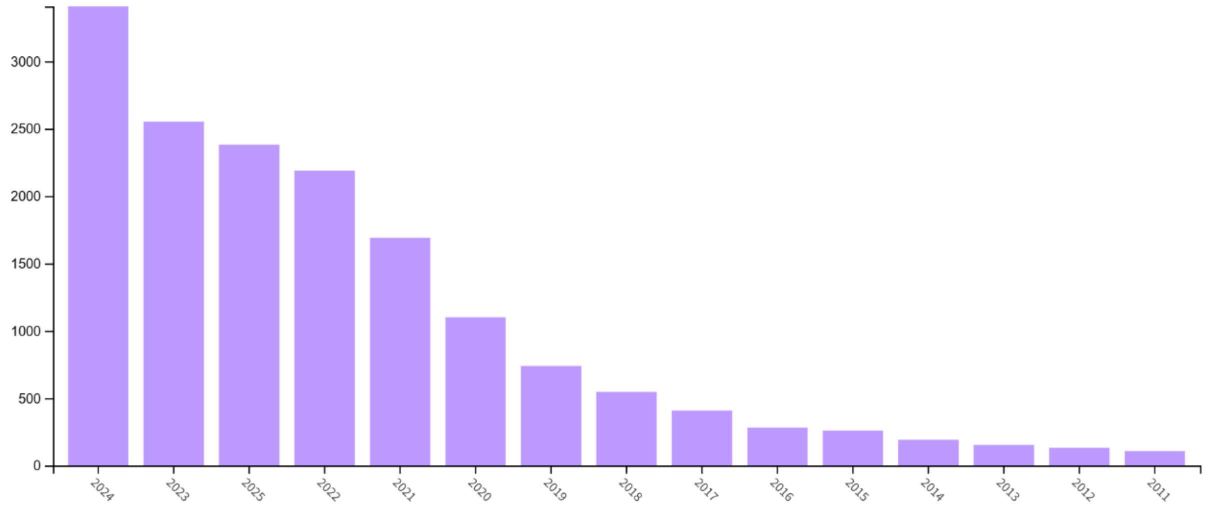

Figure S2 Annual counts of publications indexed in Web of Science mentioning single-cell omics terms between 2011 and 2024. The data were downloaded directly from the WoS database and plotted as yearly totals.

Supplementary Note S2

To refine the scope of genomic search terms, we tested more targeted phrases like "genome sequencing," "genomic sequencing," "next-generation sequencing" (NGS), and "high-throughput sequencing". These terms are used interchangeably across studies and often overlap across omics types including transcriptomics and epigenomics. To ensure accurate classification, we excluded terms that do not directly indicate the omics layer being studied. The final query development prioritised biological specificity over sequencing technology to better capture true genomic studies.

Table S8 Investigating the Genomics landscape through varied WoS search queries

**Search term**	**Number of Publications**
TS=("genomic*" OR "genome sequencing" OR "genomic sequencing" OR "next-generation sequencing" OR "high-throughput sequencing "OR "massively parallel sequencing" OR "sequencing" OR "NGS")	862,594
TS=genomic*	406,235
TS="genome sequencing"	44,971
TS="genomic sequencing"	4,696
TS="next-generation sequencing"	59,783
TS="high-throughput sequencing"	31,042
TS="massively parallel sequencing"	2,418
TS=sequencing	2,215,621
TS=NGS	25,748
TS=(genomic* AND "genomic sequencing")	4,696
TS=(genomic* AND "next-generation sequencing")	13,710
TS=(genomic* AND "high-throughput sequencing")	3,657
TS=(genomic* AND "massively parallel sequencing")	680
TS=(genomic* AND sequencing)	204,925
TS=(genomic* AND NGS)	5,813
TS=("genome sequencing" AND "genomic sequencing")	512
TS=("genome sequencing" AND "next-generation sequencing")	2899
TS=("genome sequencing" AND "high-throughput sequencing")	616
TS=("genome sequencing" AND "massively parallel sequencing")	154
TS=("genome sequencing" AND sequencing)	44,971
TS=("genome sequencing" AND NGS)	1,231
TS=("genomic sequencing" AND "next-generation sequencing")	309
TS=("genomic sequencing" AND "high-throughput sequencing")	49
TS=("genomic sequencing" AND "massively parallel sequencing")	14
TS=("genomic sequencing" AND NGS)	142
TS=("next-generation sequencing" AND "high-throughput sequencing")	1,548

TS=("next-generation sequencing" AND "massively parallel sequencing")	406
TS=("next-generation sequencing" AND sequencing)	59,783
TS=("next-generation sequencing" AND NGS)	19,625
TS=("high-throughput sequencing" AND "massively parallel sequencing")	74
TS=("high-throughput sequencing" AND sequencing)	31,042
TS=("high-throughput sequencing" AND NGS)	624
TS=("massively parallel sequencing" AND sequencing)	2418
TS=("massively parallel sequencing" AND NGS)	186
TS=(sequencing AND NGS)	22,362
TS=(“genomic*” OR "genom* sequencing” OR "next-generation sequencing" OR "high-throughput sequencing"OR NGS)	509,338
TS=(genomic* OR "genom* sequencing")	433,838

Table S9 Development of Transcriptomics and Epigenomic Search Query

**Search term**	**Number of Papers**
TS=(“transcriptomic*” OR "transcriptom* sequencing” OR "RNA seq*”)	209,002
TS=(“epigenomic*” OR "epigenom* sequencing")	6,587

Supplementary Note S3

To capture studies using mass spectrometry and NMR in metabolomics and proteomics, we combined discipline-specific and technique-based terms *Table S10* While “NMR” alone retrieved over 500,000 records and “nuclear magnetic resonance” over 146,187, combining both terms refined results to 94,348, reflecting more context-specific usage. For metabolomics, pairing “metabolo*” with “mass spectrometry” or NMR improved precision by focusing on platform-based studies. In proteomics, we tested variations combining “proteomic*”, “protein*”, and “mass spectrometry”. A search using “protein*” AND “mass spectrometry” returned 223,490 results, while broadening to “proteo* AND mass spectrometry” increased coverage to 141,245 , as “proteo*” includes related terms like proteome and protein.

Table S10 Development of Metabolomics and Proteomics Seach Query

**Search term**	**Number of Publications**
TS= “NMR”	555,786
TS="nuclear magnetic resonance"	146,187

TS=(“NMR” AND "nuclear magnetic resonance")	94,348
TS=(“metabolomic*”OR ("metabol*" AND "mass spectrometry") OR ("metabol*" AND “NMR”))	188,724
TS=(“metabolomic*”OR ("metabolo*" AND "mass spectrometry") OR ("metabolo*" AND “NMR”))	76,111
TS=(“proteomic*”OR ("protein*" AND "mass spectrometry"))	223,490
TS=(“proteomic*” OR ("proteo*" AND "mass spectrometry"))	141,245
TS=(“proteomic*” OR ("proteo*" AND "mass spectrometry") OR ("protein*" AND "mass spectrometry"))	225,491

Table S11 Interactive building of multi-omics search query dictionary

**Combination**	**Search Query**	**Number of Studies**
Genomics + Transcriptomics	TS=((“genomic*” OR "genom* sequencing") AND ("transcriptomic*" OR "transcriptom* sequencing" OR "RNA seq*"))	25,772
Genomics + Epigenomics	TS=((genomic* OR "genom* sequencing") AND ("epigenomic*" OR "epigenom* sequencing"))	2,505
Genomics + Metabolomics	TS=(("genomic*" OR "genom* sequencing") AND ("metabolomic*" OR ("metabolo*" AND "mass spectrometry") OR ("metabolo*" AND "NMR")))	3,989
Genomics + Proteomics	TS=(("genomic*" OR "genom* sequencing") AND ("proteomic*" OR ("proteo*" AND "mass spectrometry")))	11,137
Transcriptomics + Epigenomics	TS=(("transcriptomic*" OR "transcriptom* sequencing" OR "RNA seq*") AND ("epigenomic*" OR "epigenom* sequencing"))	1,905
Transcriptomics + Metabolomics	TS=(("transcriptomic*" OR "transcriptom* sequencing" OR "RNA seq*") AND ("metabolomic*" OR ("metabolo*" AND "mass spectrometry") OR ("metabolo*" AND "NMR")))	9,975
Transcriptomics + Proteomics	TS=(("transcriptomic*" OR "transcriptom* sequencing" OR "RNA seq*") AND ("proteomic*" OR ("proteo*" AND "mass spectrometry")))	12,262
Epigenomics + Metabolomics	TS=(("epigenomic*" OR "epigenom* sequencing") AND ("metabolomic*" OR ("metabolo*" AND "mass spectrometry") OR ("metabolo*" AND "NMR")))	234
Epigenomics + Proteomics	TS=(("epigenomic*" OR "epigenom* sequencing") AND ("proteomic*" OR ("proteo*" AND "mass spectrometry")))	423
Metabolomics + Proteomics	TS=(("metabolomic*" OR ("metabolo*" AND "mass spectrometry") OR ("metabolo*" AND "NMR")) AND ("proteomic*" OR ("proteo*" AND "mass spectrometry")))	6,696

Genomics + Transcriptomics + Epigenomics	TS=(("genomic*" OR "genom* sequencing") AND ("transcriptomic*" OR "transcriptom* sequencing" OR "RNA seq*") AND ("epigenomic*" OR "epigenom* sequencing"))	796
Genomics + Transcriptomics + Metabolomics	TS=(("genomic*" OR "genom* sequencing") AND ("transcriptomic*" OR "transcriptom* sequencing" OR "RNA seq*") AND ("metabolomic*" OR ("metabolo*" AND "mass spectrometry") OR ("metabolo*" AND "NMR")))	1,265
Genomics + Transcriptomics + Proteomics	TS=(("genomic*" OR "genom* sequencing") AND ("transcriptomic*" OR "transcriptom* sequencing" OR "RNA seq*") AND ("proteomic*" OR ("proteo*" AND "mass spectrometry")))	2,553
Genomics + Epigenomics + Metabolomics	TS=(("genomic*" OR "genom* sequencing") AND ("epigenomic*" OR "epigenom* sequencing") AND ("metabolomic*" OR ("metabolo*" AND "mass spectrometry") OR ("metabolo*" AND "NMR")))	149
Genomics + Epigenomics + Proteomics	TS=(("genomic*" OR "genom* sequencing") AND ("epigenomic*" OR "epigenom* sequencing") AND ("proteomic*" OR ("proteo*" AND "mass spectrometry")))	268
Genomics + Metabolomics + Proteomics	TS=(("genomic*" OR "genom* sequencing") AND ("metabolomic*" OR ("metabolo*" AND "mass spectrometry") OR ("metabolo*" AND "NMR")) AND ("proteomic*" OR ("proteo*" AND "mass spectrometry")))	1,437
Transcriptomics + Epigenomics + Metabolomics	TS=(("transcriptomic*" OR "transcriptom* sequencing" OR "RNA seq*") AND ("epigenomic*" OR "epigenom* sequencing") AND ("metabolomic*" OR ("metabolo*" AND "mass spectrometry") OR ("metabolo*" AND "NMR")))	173
Transcriptomics + Epigenomics + Proteomics	TS=(("transcriptomic*" OR "transcriptom* sequencing" OR "RNA seq*") AND ("epigenomic*" OR "epigenom* sequencing") AND ("proteomic*" OR ("proteo*" AND "mass spectrometry")))	296
Transcriptomics + Metabolomics + Proteomics	TS=(("transcriptomic*" OR "transcriptom* sequencing" OR "RNA seq*") AND ("metabolomic*" OR ("metabolo*" AND "mass spectrometry") OR ("metabolo*" AND "NMR")) AND ("proteomic*" OR ("proteo*" AND "mass spectrometry")))	1,875
Epigenomics + Metabolomics + Proteomics	TS=(("epigenomic*" OR "epigenom* sequencing") AND ("metabolomic*" OR ("metabolo*" AND "mass spectrometry") OR ("metabolo*" AND "NMR")) AND ("proteomic*" OR ("proteo*" AND "mass spectrometry")))	159
Genomics + Transcriptomics + Epigenomics + Metabolomics	TS=(("genomic*" OR "genom* sequencing") AND ("transcriptomic*" OR "transcriptom* sequencing" OR "RNA seq*") AND ("epigenomic*" OR "epigenom* sequencing") AND ("metabolomic*" OR ("metabolo*"	111

	AND "mass spectrometry") OR ("metabolo*" AND "NMR")))	
Genomics + Transcriptomics + Epigenomics + Proteomics	TS=(("genomic*" OR "genom* sequencing") AND ("transcriptomic*" OR "transcriptom* sequencing" OR "RNA seq*") AND ("epigenomic*" OR "epigenom* sequencing") AND ("proteomic*" OR ("proteo*" AND "mass spectrometry")))	187
Genomics + Transcriptomics + Metabolomics + Proteomics	TS=(("genomic*" OR "genom* sequencing") AND ("transcriptomic*" OR "transcriptom* sequencing" OR "RNA seq*") AND ("metabolomic*" OR ("metabolo*" AND "mass spectrometry") OR ("metabolo*" AND "NMR")) AND ("proteomic*" OR ("proteo*" AND "mass spectrometry")))	711
Genomics + Epigenomics + Metabolomics + Proteomics	TS=(("genomic*" OR "genom* sequencing") AND ("epigenomic*" OR "epigenom* sequencing") AND ("metabolomic*" OR ("metabolo*" AND "mass spectrometry") OR ("metabolo*" AND "NMR")) AND ("proteomic*" OR ("proteo*" AND "mass spectrometry")))	120
Transcriptomics + Epigenomics + Metabolomics + Proteomics	TS=(("transcriptomic*" OR "transcriptom* sequencing" OR "RNA seq*") AND ("epigenomic*" OR "epigenom* sequencing") AND ("metabolomic*" OR ("metabolo*" AND "mass spectrometry") OR ("metabolo*" AND "NMR")) AND ("proteomic*" OR ("proteo*" AND "mass spectrometry")))	129
Genomics + Transcriptomics + Epigenomics + Metabolomics + Proteomics	TS=(("genomic*" OR "genom* sequencing") AND ("transcriptomic*" OR "transcriptom* sequencing" OR "RNA seq*") AND ("epigenomic*" OR "epigenom* sequencing") AND ("metabolomic*" OR ("metabolo*" AND "mass spectrometry") OR ("metabolo*" AND "NMR")) AND ("proteomic*" OR ("proteo*" AND "mass spectrometry")))	97

Table S12 Stepwise Generation of search query

**Search**	**Number of Publications**
TS=(((("genomic*" OR "genom* sequencing") AND ("transcriptomic*" OR "transcriptom* sequencing" OR "RNA seq*")) OR (("genomic*" OR "genom* sequencing") AND ("epigenomic*" OR "epigenom* sequencing")) OR (("genomic*" OR "genom* sequencing") AND ("metabolomic*" OR ("metabolo*" AND "mass spectrometry") OR ("metabolo*" AND "NMR"))) OR (("genomic*" OR "genom* sequencing") AND ("proteomic*" OR ("proteo*" AND "mass spectrometry"))) OR (("transcriptomic*" OR "transcriptom* sequencing" OR "RNA seq*") AND ("epigenomic*" OR "epigenom* sequencing")) OR (("transcriptomic*" OR "transcriptom* sequencing" OR "RNA seq*") AND ("metabolomic*" OR ("metabolo*" AND "mass spectrometry") OR	60,352

("metabolo*" AND "NMR"))) OR (("transcriptomic*" OR "transcriptom* sequencing" OR "RNA seq*") AND ("proteomic*" OR ("proteo*" AND "mass spectrometry"))) OR (("epigenomic*" OR "epigenom* sequencing") AND ("metabolomic*" OR ("metabolo*" AND "mass spectrometry") OR ("metabolo*" AND "NMR"))) OR (("epigenomic*" OR "epigenom* sequencing") AND ("proteomic*" OR ("proteo*" AND "mass spectrometry"))) OR (("metabolomic*" OR ("metabolo*" AND "mass spectrometry") OR ("metabolo*" AND "NMR")) AND ("proteomic*" OR ("proteo*" AND "mass spectrometry")))))	
TS=(((("genomic*" OR "genom* sequencing") AND ("transcriptomic*" OR "transcriptom* sequencing" OR "RNA seq*")) OR (("genomic*" OR "genom* sequencing") AND ("epigenomic*" OR "epigenom* sequencing")) OR (("genomic*" OR "genom* sequencing") AND ("metabolomic*" OR ("metabolo*" AND "mass spectrometry") OR ("metabolo*" AND "NMR"))) OR (("genomic*" OR "genom* sequencing") AND ("proteomic*" OR ("proteo*" AND "mass spectrometry"))) OR (("transcriptomic*" OR "transcriptom* sequencing" OR "RNA seq*") AND ("epigenomic*" OR "epigenom* sequencing")) OR (("transcriptomic*" OR "transcriptom* sequencing" OR "RNA seq*") AND ("metabolomic*" OR ("metabolo*" AND "mass spectrometry") OR ("metabolo*" AND "NMR"))) OR (("transcriptomic*" OR "transcriptom* sequencing" OR "RNA seq*") AND ("proteomic*" OR ("proteo*" AND "mass spectrometry"))) OR (("epigenomic*" OR "epigenom* sequencing") AND ("metabolomic*" OR ("metabolo*" AND "mass spectrometry") OR ("metabolo*" AND "NMR"))) OR (("epigenomic*" OR "epigenom* sequencing") AND ("proteomic*" OR ("proteo*" AND "mass spectrometry"))) OR (("metabolomic*" OR ("metabolo*" AND "mass spectrometry") OR ("metabolo*" AND "NMR")) AND ("proteomic*" OR ("proteo*" AND "mass spectrometry")))) AND ("multiomic*" OR "multi-omic*" OR "crossomic*" OR "cross-omic*" OR "cross omic*" OR "panomic*" OR "pan-omic*" OR "transomic*" OR "trans-omic*" OR "multiple omic*"))	5,246
TS=(((("genomic*" OR "genom* sequencing") AND ("transcriptomic*" OR "transcriptom* sequencing" OR "RNA seq*")) OR (("genomic*" OR "genom* sequencing") AND ("epigenomic*" OR "epigenom* sequencing")) OR (("genomic*" OR "genom* sequencing") AND ("metabolomic*" OR ("metabolo*" AND "mass spectrometry") OR ("metabolo*" AND "NMR"))) OR (("genomic*" OR "genom* sequencing") AND ("proteomic*" OR ("proteo*" AND "mass spectrometry"))) OR (("transcriptomic*" OR "transcriptom* sequencing" OR "RNA seq*") AND ("epigenomic*" OR "epigenom* sequencing")) OR (("transcriptomic*" OR "transcriptom* sequencing" OR "RNA seq*") AND ("metabolomic*" OR ("metabolo*" AND "mass spectrometry") OR ("metabolo*" AND "NMR"))) OR (("transcriptomic*" OR "transcriptom* sequencing" OR "RNA seq*") AND ("proteomic*" OR ("proteo*" AND "mass spectrometry"))) OR (("epigenomic*" OR "epigenom* sequencing") AND ("metabolomic*" OR ("metabolo*" AND "mass spectrometry") OR ("metabolo*" AND "NMR"))) OR (("epigenomic*" OR "epigenom* sequencing") AND ("proteomic*" OR ("proteo*" AND "mass spectrometry"))) OR (("metabolomic*" OR ("metabolo*" AND "mass	1,173

spectrometry") OR ("metabolo*" AND "NMR")) AND ("proteomic*" OR ("proteo*" AND "mass spectrometry")))) AND ("multiomic*" OR "multi-omic*" OR "crossomic*" OR "cross-omic*" OR "cross omic*" OR "panomic*" OR "pan-omic*" OR "transomic*" OR "trans-omic*" OR "multiple omic*") AND ("integration" OR "fusion" OR "integrative analysis"))	
TS=(((("genomic*" OR "genom* sequencing") AND ("transcriptomic*" OR "transcriptom* sequencing" OR "RNA seq*")) OR (("genomic*" OR "genom* sequencing") AND ("epigenomic*" OR "epigenom* sequencing")) OR (("genomic*" OR "genom* sequencing") AND ("metabolomic*" OR ("metabolo*" AND "mass spectrometry") OR ("metabolo*" AND "NMR"))) OR (("genomic*" OR "genom* sequencing") AND ("proteomic*" OR ("proteo*" AND "mass spectrometry"))) OR (("transcriptomic*" OR "transcriptom* sequencing" OR "RNA seq*") AND ("epigenomic*" OR "epigenom* sequencing")) OR (("transcriptomic*" OR "transcriptom* sequencing" OR "RNA seq*") AND ("metabolomic*" OR ("metabolo*" AND "mass spectrometry") OR ("metabolo*" AND "NMR"))) OR (("transcriptomic*" OR "transcriptom* sequencing" OR "RNA seq*") AND ("proteomic*" OR ("proteo*" AND "mass spectrometry"))) OR (("epigenomic*" OR "epigenom* sequencing") AND ("metabolomic*" OR ("metabolo*" AND "mass spectrometry") OR ("metabolo*" AND "NMR"))) OR (("epigenomic*" OR "epigenom* sequencing") AND ("proteomic*" OR ("proteo*" AND "mass spectrometry"))) OR (("metabolomic*" OR ("metabolo*" AND "mass spectrometry") OR ("metabolo*" AND "NMR")) AND ("proteomic*" OR ("proteo*" AND "mass spectrometry"))) OR (("genomic*" OR "genom* sequencing") AND "phosphoproteomic*") OR (("genomic*" OR "genom* sequencing") AND "epitranscriptomic*") OR (("genomic*" OR "genom* sequencing") AND "lipidomic*") OR (("genomic*" OR "genom* sequencing") AND "glycomic*") OR (("genomic*" OR "genom* sequencing") AND "pharmacogenomic*") OR (("genomic*" OR "genom* sequencing") AND "fluxomic*") OR (("genomic*" OR "genom* sequencing") AND "interactomic*") OR (("transcriptomic*" OR "transcriptom* sequencing" OR "RNA seq*") AND "phosphoproteomic*") OR (("transcriptomic*" OR "transcriptom* sequencing" OR "RNA seq*") AND "epitranscriptomic*") OR (("transcriptomic*" OR "transcriptom* sequencing" OR "RNA seq*") AND "lipidomic*") OR (("transcriptomic*" OR "transcriptom* sequencing" OR "RNA seq*") AND "glycomic*") OR (("transcriptomic*" OR "transcriptom* sequencing" OR "RNA seq*") AND "pharmacogenomic*") OR (("transcriptomic*" OR "transcriptom* sequencing" OR "RNA seq*") AND "fluxomic*") OR (("transcriptomic*" OR "transcriptom* sequencing" OR "RNA seq*") AND "interactomic*") OR (("epigenomic*" OR "epigenom* sequencing") AND "phosphoproteomic*") OR (("epigenomic*" OR "epigenom* sequencing") AND "epitranscriptomic*") OR (("epigenomic*" OR "epigenom* sequencing") AND "lipidomic*") OR (("epigenomic*" OR "epigenom* sequencing") AND "glycomic*") OR (("epigenomic*" OR "epigenom* sequencing") AND "pharmacogenomic*") OR (("epigenomic*" OR "epigenom* sequencing") AND "fluxomic*") OR	68,888

(("epigenomic*" OR "epigenom* sequencing") AND "interactomic*") OR (("metabolomic*" OR ("metabolo*" AND "mass spectrometry") OR ("metabolo*" AND "NMR")) AND "phosphoproteomic*") OR (("metabolomic*" OR ("metabolo*" AND "mass spectrometry") OR ("metabolo*" AND "NMR")) AND "epitranscriptomic*") OR (("metabolomic*" OR ("metabolo*" AND "mass spectrometry") OR ("metabolo*" AND "NMR")) AND "lipidomic*") OR (("metabolomic*" OR ("metabolo*" AND "mass spectrometry") OR ("metabolo*" AND "NMR")) AND "glycomic*") OR (("metabolomic*" OR ("metabolo*" AND "mass spectrometry") OR ("metabolo*" AND "NMR")) AND "pharmacogenomic*") OR (("metabolomic*" OR ("metabolo*" AND "mass spectrometry") OR ("metabolo*" AND "NMR")) AND "fluxomic*") OR (("metabolomic*" OR ("metabolo*" AND "mass spectrometry") OR ("metabolo*" AND "NMR")) AND "interactomic*") OR (("proteomic*" OR ("proteo*" AND "mass spectrometry")) AND "phosphoproteomic*") OR (("proteomic*" OR ("proteo*" AND "mass spectrometry")) AND "epitranscriptomic*") OR (("proteomic*" OR ("proteo*" AND "mass spectrometry")) AND "lipidomic*") OR (("proteomic*" OR ("proteo*" AND "mass spectrometry")) AND "glycomic*") OR (("proteomic*" OR ("proteo*" AND "mass spectrometry")) AND "pharmacogenomic*") OR (("proteomic*" OR ("proteo*" AND "mass spectrometry")) AND "fluxomic*") OR (("proteomic*" OR ("proteo*" AND "mass spectrometry")) AND "interactomic*")))	
TS=(((("genomic*" OR "genom* sequencing") AND ("transcriptomic*" OR "transcriptom* sequencing" OR "RNA seq*")) OR (("genomic*" OR "genom* sequencing") AND ("epigenomic*" OR "epigenom* sequencing")) OR (("genomic*" OR "genom* sequencing") AND ("metabolomic*" OR ("metabolo*" AND "mass spectrometry") OR ("metabolo*" AND "NMR"))) OR (("genomic*" OR "genom* sequencing") AND ("proteomic*" OR ("proteo*" AND "mass spectrometry"))) OR (("transcriptomic*" OR "transcriptom* sequencing" OR "RNA seq*") AND ("epigenomic*" OR "epigenom* sequencing")) OR (("transcriptomic*" OR "transcriptom* sequencing" OR "RNA seq*") AND ("metabolomic*" OR ("metabolo*" AND "mass spectrometry") OR ("metabolo*" AND "NMR"))) OR (("transcriptomic*" OR "transcriptom* sequencing" OR "RNA seq*") AND ("proteomic*" OR ("proteo*" AND "mass spectrometry"))) OR (("epigenomic*" OR "epigenom* sequencing") AND ("metabolomic*" OR ("metabolo*" AND "mass spectrometry") OR ("metabolo*" AND "NMR"))) OR (("epigenomic*" OR "epigenom* sequencing") AND ("proteomic*" OR ("proteo*" AND "mass spectrometry"))) OR (("metabolomic*" OR ("metabolo*" AND "mass spectrometry") OR ("metabolo*" AND "NMR")) AND ("proteomic*" OR ("proteo*" AND "mass spectrometry"))) OR (("genomic*" OR "genom* sequencing") AND "phosphoproteomic*") OR (("genomic*" OR "genom* sequencing") AND "epitranscriptomic*") OR (("genomic*" OR "genom* sequencing") AND "lipidomic*") OR (("genomic*" OR "genom* sequencing") AND "glycomic*") OR (("genomic*" OR "genom* sequencing") AND "pharmacogenomic*") OR (("genomic*" OR "genom* sequencing") AND "fluxomic*") OR (("genomic*" OR "genom* sequencing") AND "interactomic*") OR (("transcriptomic*" OR	5,563

"transcriptom* sequencing" OR "RNA seq*") AND "phosphoproteomic*") OR (("transcriptomic*" OR "transcriptom* sequencing" OR "RNA seq*") AND "epitranscriptomic*") OR (("transcriptomic*" OR "transcriptom* sequencing" OR "RNA seq*") AND "lipidomic*") OR (("transcriptomic*" OR "transcriptom* sequencing" OR "RNA seq*") AND "glycomic*") OR (("transcriptomic*" OR "transcriptom* sequencing" OR "RNA seq*") AND "pharmacogenomic*") OR (("transcriptomic*" OR "transcriptom* sequencing" OR "RNA seq*") AND "fluxomic*") OR (("transcriptomic*" OR "transcriptom* sequencing" OR "RNA seq*") AND "interactomic*") OR (("epigenomic*" OR "epigenom* sequencing") AND "phosphoproteomic*") OR (("epigenomic*" OR "epigenom* sequencing") AND "epitranscriptomic*") OR (("epigenomic*" OR "epigenom* sequencing") AND "lipidomic*") OR (("epigenomic*" OR "epigenom* sequencing") AND "glycomic*") OR (("epigenomic*" OR "epigenom* sequencing") AND "pharmacogenomic*") OR (("epigenomic*" OR "epigenom* sequencing") AND "fluxomic*") OR (("epigenomic*" OR "epigenom* sequencing") AND "interactomic*") OR (("metabolomic*" OR ("metabolo*" AND "mass spectrometry") OR ("metabolo*" AND "NMR")) AND "phosphoproteomic*") OR (("metabolomic*" OR ("metabolo*" AND "mass spectrometry") OR ("metabolo*" AND "NMR")) AND "epitranscriptomic*") OR (("metabolomic*" OR ("metabolo*" AND "mass spectrometry") OR ("metabolo*" AND "NMR")) AND "lipidomic*") OR (("metabolomic*" OR ("metabolo*" AND "mass spectrometry") OR ("metabolo*" AND "NMR")) AND "glycomic*") OR (("metabolomic*" OR ("metabolo*" AND "mass spectrometry") OR ("metabolo*" AND "NMR")) AND "pharmacogenomic*") OR (("metabolomic*" OR ("metabolo*" AND "mass spectrometry") OR ("metabolo*" AND "NMR")) AND "fluxomic*") OR (("metabolomic*" OR ("metabolo*" AND "mass spectrometry") OR ("metabolo*" AND "NMR")) AND "interactomic*") OR (("proteomic*" OR ("proteo*" AND "mass spectrometry")) AND "phosphoproteomic*") OR (("proteomic*" OR ("proteo*" AND "mass spectrometry")) AND "epitranscriptomic*") OR (("proteomic*" OR ("proteo*" AND "mass spectrometry")) AND "lipidomic*") OR (("proteomic*" OR ("proteo*" AND "mass spectrometry")) AND "glycomic*") OR (("proteomic*" OR ("proteo*" AND "mass spectrometry")) AND "pharmacogenomic*") OR (("proteomic*" OR ("proteo*" AND "mass spectrometry")) AND "fluxomic*") OR (("proteomic*" OR ("proteo*" AND "mass spectrometry")) AND "interactomic*")) AND ("multiomic*" OR "multi-omic*" OR "crossomic*" OR "cross-omic*" OR "cross omic*" OR "panomic*" OR "pan-omic*" OR "transomic*" OR "trans-omic*" OR "multiple omic*"))	
TS=(((("genomic*" OR "genom* sequencing") AND ("transcriptomic*" OR "transcriptom* sequencing" OR "RNA seq*")) OR (("genomic*" OR "genom* sequencing") AND ("epigenomic*" OR "epigenom* sequencing")) OR (("genomic*" OR "genom* sequencing") AND ("metabolomic*" OR ("metabolo*" AND "mass spectrometry") OR ("metabolo*" AND "NMR"))) OR (("genomic*" OR "genom* sequencing") AND ("proteomic*" OR ("proteo*" AND "mass	1,231

spectrometry"))) OR (("transcriptomic*" OR "transcriptom* sequencing" OR "RNA seq*") AND ("epigenomic*" OR "epigenom* sequencing")) OR (("transcriptomic*" OR "transcriptom* sequencing" OR "RNA seq*") AND ("metabolomic*" OR ("metabolo*" AND "mass spectrometry") OR ("metabolo*" AND "NMR"))) OR (("transcriptomic*" OR "transcriptom* sequencing" OR "RNA seq*") AND ("proteomic*" OR ("proteo*" AND "mass spectrometry"))) OR (("epigenomic*" OR "epigenom* sequencing") AND ("metabolomic*" OR ("metabolo*" AND "mass spectrometry") OR ("metabolo*" AND "NMR"))) OR (("epigenomic*" OR "epigenom* sequencing") AND ("proteomic*" OR ("proteo*" AND "mass spectrometry"))) OR (("metabolomic*" OR ("metabolo*" AND "mass spectrometry") OR ("metabolo*" AND "NMR")) AND ("proteomic*" OR ("proteo*" AND "mass spectrometry"))) OR (("genomic*" OR "genom* sequencing") AND "phosphoproteomic*") OR (("genomic*" OR "genom* sequencing") AND "epitranscriptomic*") OR (("genomic*" OR "genom* sequencing") AND "lipidomic*") OR (("genomic*" OR "genom* sequencing") AND "glycomic*") OR (("genomic*" OR "genom* sequencing") AND "pharmacogenomic*") OR (("genomic*" OR "genom* sequencing") AND "fluxomic*") OR (("genomic*" OR "genom* sequencing") AND "interactomic*") OR (("transcriptomic*" OR "transcriptom* sequencing" OR "RNA seq*") AND "phosphoproteomic*") OR (("transcriptomic*" OR "transcriptom* sequencing" OR "RNA seq*") AND "epitranscriptomic*") OR (("transcriptomic*" OR "transcriptom* sequencing" OR "RNA seq*") AND "lipidomic*") OR (("transcriptomic*" OR "transcriptom* sequencing" OR "RNA seq*") AND "glycomic*") OR (("transcriptomic*" OR "transcriptom* sequencing" OR "RNA seq*") AND "pharmacogenomic*") OR (("transcriptomic*" OR "transcriptom* sequencing" OR "RNA seq*") AND "fluxomic*") OR (("transcriptomic*" OR "transcriptom* sequencing" OR "RNA seq*") AND "interactomic*") OR (("epigenomic*" OR "epigenom* sequencing") AND "phosphoproteomic*") OR (("epigenomic*" OR "epigenom* sequencing") AND "epitranscriptomic*") OR (("epigenomic*" OR "epigenom* sequencing") AND "lipidomic*") OR (("epigenomic*" OR "epigenom* sequencing") AND "glycomic*") OR (("epigenomic*" OR "epigenom* sequencing") AND "pharmacogenomic*") OR (("epigenomic*" OR "epigenom* sequencing") AND "fluxomic*") OR (("epigenomic*" OR "epigenom* sequencing") AND "interactomic*") OR (("metabolomic*" OR ("metabolo*" AND "mass spectrometry") OR ("metabolo*" AND "NMR")) AND "phosphoproteomic*") OR (("metabolomic*" OR ("metabolo*" AND "mass spectrometry") OR ("metabolo*" AND "NMR")) AND "epitranscriptomic*") OR (("metabolomic*" OR ("metabolo*" AND "mass spectrometry") OR ("metabolo*" AND "NMR")) AND "lipidomic*") OR (("metabolomic*" OR ("metabolo*" AND "mass spectrometry") OR ("metabolo*" AND "NMR")) AND "glycomic*") OR (("metabolomic*" OR ("metabolo*" AND "mass spectrometry") OR ("metabolo*" AND "NMR")) AND "pharmacogenomic*") OR (("metabolomic*" OR ("metabolo*" AND "mass spectrometry") OR ("metabolo*" AND "NMR")) AND "fluxomic*") OR (("metabolomic*" OR ("metabolo*" AND "mass spectrometry") OR	

("metabolo*" AND "NMR")) AND "interactomic*") OR (("proteomic*" OR ("proteo*" AND "mass spectrometry")) AND "phosphoproteomic*") OR (("proteomic*" OR ("proteo*" AND "mass spectrometry")) AND "epitranscriptomic*") OR (("proteomic*" OR ("proteo*" AND "mass spectrometry")) AND "lipidomic*") OR (("proteomic*" OR ("proteo*" AND "mass spectrometry")) AND "glycomic*") OR (("proteomic*" OR ("proteo*" AND "mass spectrometry")) AND "pharmacogenomic*") OR (("proteomic*" OR ("proteo*" AND "mass spectrometry")) AND "fluxomic*") OR (("proteomic*" OR ("proteo*" AND "mass spectrometry")) AND "interactomic*")) AND ("multiomic*" OR "multi-omic*" OR "crossomic*" OR "cross-omic*" OR "cross omic*" OR "panomic*" OR "pan-omic*" OR "transomic*" OR "trans-omic*" OR "multiple omic*") AND ("integration" OR "fusion" OR "integrative analysis"))	

.

Table S13 Combining Cell Line search query with Multi-omics search query

Search Term	Number of publications
TS=((("Chinese Hamster Ovary" OR "CHO cell*" OR "CHO-K1" OR "CHO-S" OR "CHO-DG44" OR "CHO-DUK" OR "CHOZN" OR "CHO-T" OR "CHO-Lec*" OR "CHO-H" OR "CHO-AT3-2" OR "CHO-C" OR "CHO-M" OR "CHO-AI") AND "cell*" NOT ("Human Embryonic Kidney" OR "HEK293*" OR "HEK 293*") AND "cell*") AND ((((("genomic*" OR "genom* sequencing") AND ("transcriptomic*" OR "transcriptom* sequencing" OR "RNA seq*")) OR (("genomic*" OR "genom* sequencing") AND ("epigenomic*" OR "epigenom* sequencing")) OR (("genomic*" OR "genom* sequencing") AND ("metabolomic*" OR ("metabolo*" AND "mass spectrometry") OR ("metabolo*" AND "NMR"))) OR (("genomic*" OR "genom* sequencing") AND ("proteomic*" OR ("proteo*" AND "mass spectrometry"))) OR (("transcriptomic*" OR "transcriptom* sequencing" OR "RNA seq*") AND ("epigenomic*" OR "epigenom* sequencing")) OR (("transcriptomic*" OR "transcriptom* sequencing" OR "RNA seq*") AND ("metabolomic*" OR ("metabolo*" AND "mass spectrometry") OR ("metabolo*" AND "NMR"))) OR (("transcriptomic*" OR "transcriptom* sequencing" OR "RNA seq*") AND ("proteomic*" OR ("proteo*" AND "mass spectrometry"))) OR (("epigenomic*" OR "epigenom* sequencing") AND ("metabolomic*" OR ("metabolo*" AND "mass spectrometry") OR ("metabolo*" AND "NMR"))) OR (("epigenomic*" OR "epigenom* sequencing") AND ("proteomic*" OR ("proteo*" AND "mass spectrometry"))) OR (("metabolomic*" OR ("metabolo*" AND "mass spectrometry") OR ("metabolo*" AND "NMR")) AND ("proteomic*" OR ("proteo*" AND "mass spectrometry"))) OR (("genomic*" OR "genom* sequencing") AND "phosphoproteomic*") OR (("genomic*" OR "genom* sequencing") AND "epitranscriptomic*") OR (("genomic*" OR "genom* sequencing") AND "lipidomic*") OR (("genomic*" OR "genom* sequencing") AND "glycomic*") OR (("genomic*" OR "genom*	105

sequencing") AND "pharmacogenomic*") OR (("genomic*" OR "genom* sequencing") AND "fluxomic*") OR (("genomic*" OR "genom* sequencing") AND "interactomic*") OR (("transcriptomic*" OR "transcriptom* sequencing" OR "RNA seq*") AND "phosphoproteomic*") OR (("transcriptomic*" OR "transcriptom* sequencing" OR "RNA seq*") AND "epitranscriptomic*") OR (("transcriptomic*" OR "transcriptom* sequencing" OR "RNA seq*") AND "lipidomic*") OR (("transcriptomic*" OR "transcriptom* sequencing" OR "RNA seq*") AND "glycomic*") OR (("transcriptomic*" OR "transcriptom* sequencing" OR "RNA seq*") AND "pharmacogenomic*") OR (("transcriptomic*" OR "transcriptom* sequencing" OR "RNA seq*") AND "fluxomic*") OR (("transcriptomic*" OR "transcriptom* sequencing" OR "RNA seq*") AND "interactomic*") OR (("epigenomic*" OR "epigenom* sequencing") AND "phosphoproteomic*") OR (("epigenomic*" OR "epigenom* sequencing") AND "epitranscriptomic*") OR (("epigenomic*" OR "epigenom* sequencing") AND "lipidomic*") OR (("epigenomic*" OR "epigenom* sequencing") AND "glycomic*") OR (("epigenomic*" OR "epigenom* sequencing") AND "pharmacogenomic*") OR (("epigenomic*" OR "epigenom* sequencing") AND "fluxomic*") OR (("epigenomic*" OR "epigenom* sequencing") AND "interactomic*") OR (("metabolomic*" OR ("metabolo*" AND "mass spectrometry") OR ("metabolo*" AND "NMR")) AND "phosphoproteomic*") OR (("metabolomic*" OR ("metabolo*" AND "mass spectrometry") OR ("metabolo*" AND "NMR")) AND "epitranscriptomic*") OR (("metabolomic*" OR ("metabolo*" AND "mass spectrometry") OR ("metabolo*" AND "NMR")) AND "lipidomic*") OR (("metabolomic*" OR ("metabolo*" AND "mass spectrometry") OR ("metabolo*" AND "NMR")) AND "glycomic*") OR (("metabolomic*" OR ("metabolo*" AND "mass spectrometry") OR ("metabolo*" AND "NMR")) AND "pharmacogenomic*") OR (("metabolomic*" OR ("metabolo*" AND "mass spectrometry") OR ("metabolo*" AND "NMR")) AND "fluxomic*") OR (("metabolomic*" OR ("metabolo*" AND "mass spectrometry") OR ("metabolo*" AND "NMR")) AND "interactomic*") OR (("proteomic*" OR ("proteo*" AND "mass spectrometry")) AND "phosphoproteomic*") OR (("proteomic*" OR ("proteo*" AND "mass spectrometry")) AND "epitranscriptomic*") OR (("proteomic*" OR ("proteo*" AND "mass spectrometry")) AND "lipidomic*") OR (("proteomic*" OR ("proteo*" AND "mass spectrometry")) AND "glycomic*") OR (("proteomic*" OR ("proteo*" AND "mass spectrometry")) AND "pharmacogenomic*") OR (("proteomic*" OR ("proteo*" AND "mass spectrometry")) AND "fluxomic*") OR (("proteomic*" OR ("proteo*" AND "mass spectrometry")) AND "interactomic*")))))	
TS=((("Chinese Hamster Ovary" OR "CHO cell*" OR "CHO-K1" OR "CHO-S" OR "CHO-DG44" OR "CHO-DUK" OR "CHOZN" OR "CHO-T" OR "CHO-Lec*" OR "CHO-H" OR "CHO-AT3-2" OR "CHO-C" OR "CHO-M" OR "CHO-AI") AND "cell*" NOT ("Human Embryonic Kidney" OR "HEK293*" OR "HEK 293*") AND "cell*") AND ((((("genomic*" OR "genom* sequencing") AND ("transcriptomic*" OR "transcriptom*	14

sequencing" OR "RNA seq*")) OR (("genomic*" OR "genom* sequencing") AND ("epigenomic*" OR "epigenom* sequencing")) OR (("genomic*" OR "genom* sequencing") AND ("metabolomic*" OR ("metabolo*" AND "mass spectrometry") OR ("metabolo*" AND "NMR"))) OR (("genomic*" OR "genom* sequencing") AND ("proteomic*" OR ("proteo*" AND "mass spectrometry"))) OR (("transcriptomic*" OR "transcriptom* sequencing" OR "RNA seq*") AND ("epigenomic*" OR "epigenom* sequencing")) OR (("transcriptomic*" OR "transcriptom* sequencing" OR "RNA seq*") AND ("metabolomic*" OR ("metabolo*" AND "mass spectrometry") OR ("metabolo*" AND "NMR"))) OR (("transcriptomic*" OR "transcriptom* sequencing" OR "RNA seq*") AND ("proteomic*" OR ("proteo*" AND "mass spectrometry"))) OR (("epigenomic*" OR "epigenom* sequencing") AND ("metabolomic*" OR ("metabolo*" AND "mass spectrometry") OR ("metabolo*" AND "NMR"))) OR (("epigenomic*" OR "epigenom* sequencing") AND ("proteomic*" OR ("proteo*" AND "mass spectrometry"))) OR (("metabolomic*" OR ("metabolo*" AND "mass spectrometry") OR ("metabolo*" AND "NMR")) AND ("proteomic*" OR ("proteo*" AND "mass spectrometry"))) OR (("genomic*" OR "genom* sequencing") AND "phosphoproteomic*") OR (("genomic*" OR "genom* sequencing") AND "epitranscriptomic*") OR (("genomic*" OR "genom* sequencing") AND "lipidomic*") OR (("genomic*" OR "genom* sequencing") AND "glycomic*") OR (("genomic*" OR "genom* sequencing") AND "pharmacogenomic*") OR (("genomic*" OR "genom* sequencing") AND "fluxomic*") OR (("genomic*" OR "genom* sequencing") AND "interactomic*") OR (("transcriptomic*" OR "transcriptom* sequencing" OR "RNA seq*") AND "phosphoproteomic*") OR (("transcriptomic*" OR "transcriptom* sequencing" OR "RNA seq*") AND "epitranscriptomic*") OR (("transcriptomic*" OR "transcriptom* sequencing" OR "RNA seq*") AND "lipidomic*") OR (("transcriptomic*" OR "transcriptom* sequencing" OR "RNA seq*") AND "glycomic*") OR (("transcriptomic*" OR "transcriptom* sequencing" OR "RNA seq*") AND "pharmacogenomic*") OR (("transcriptomic*" OR "transcriptom* sequencing" OR "RNA seq*") AND "fluxomic*") OR (("transcriptomic*" OR "transcriptom* sequencing" OR "RNA seq*") AND "interactomic*") OR (("epigenomic*" OR "epigenom* sequencing") AND "phosphoproteomic*") OR (("epigenomic*" OR "epigenom* sequencing") AND "epitranscriptomic*") OR (("epigenomic*" OR "epigenom* sequencing") AND "lipidomic*") OR (("epigenomic*" OR "epigenom* sequencing") AND "glycomic*") OR (("epigenomic*" OR "epigenom* sequencing") AND "pharmacogenomic*") OR (("epigenomic*" OR "epigenom* sequencing") AND "fluxomic*") OR (("epigenomic*" OR "epigenom* sequencing") AND "interactomic*") OR (("metabolomic*" OR ("metabolo*" AND "mass spectrometry") OR ("metabolo*" AND "NMR")) AND "phosphoproteomic*") OR (("metabolomic*" OR ("metabolo*" AND "mass spectrometry") OR ("metabolo*" AND "NMR")) AND "epitranscriptomic*") OR (("metabolomic*" OR ("metabolo*" AND "mass spectrometry") OR ("metabolo*" AND "NMR")) AND "lipidomic*") OR (("metabolomic*" OR	

("metabolo*" AND "mass spectrometry") OR ("metabolo*" AND "NMR")) AND "glycomic*") OR (("metabolomic*" OR ("metabolo*" AND "mass spectrometry") OR ("metabolo*" AND "NMR")) AND "pharmacogenomic*") OR (("metabolomic*" OR ("metabolo*" AND "mass spectrometry") OR ("metabolo*" AND "NMR")) AND "fluxomic*") OR (("metabolomic*" OR ("metabolo*" AND "mass spectrometry") OR ("metabolo*" AND "NMR")) AND "interactomic*") OR (("proteomic*" OR ("proteo*" AND "mass spectrometry")) AND "phosphoproteomic*") OR (("proteomic*" OR ("proteo*" AND "mass spectrometry")) AND "epitranscriptomic*") OR (("proteomic*" OR ("proteo*" AND "mass spectrometry")) AND "lipidomic*") OR (("proteomic*" OR ("proteo*" AND "mass spectrometry")) AND "glycomic*") OR (("proteomic*" OR ("proteo*" AND "mass spectrometry")) AND "pharmacogenomic*") OR (("proteomic*" OR ("proteo*" AND "mass spectrometry")) AND "fluxomic*") OR (("proteomic*" OR ("proteo*" AND "mass spectrometry")) AND "interactomic*")))) AND ("multiomic*" OR "multi-omic*" OR "crossomic*" OR "cross-omic*" OR "cross omic*" OR "panomic*" OR "pan-omic*" OR "transomic*" OR "trans-omic*" OR "multiple omic*"))	
TS=((("Chinese Hamster Ovary" OR "CHO cell*" OR "CHO-K1" OR "CHO-S" OR "CHO-DG44" OR "CHO-DUK" OR "CHOZN" OR "CHO-T" OR "CHO-Lec*" OR "CHO-H" OR "CHO-AT3-2" OR "CHO-C" OR "CHO-M" OR "CHO-AI") AND "cell*" NOT ("Human Embryonic Kidney" OR "HEK293*" OR "HEK 293*") AND "cell*") AND ((((("genomic*" OR "genom* sequencing") AND ("transcriptomic*" OR "transcriptom* sequencing" OR "RNA seq*")) OR (("genomic*" OR "genom* sequencing") AND ("epigenomic*" OR "epigenom* sequencing")) OR (("genomic*" OR "genom* sequencing") AND ("metabolomic*" OR ("metabolo*" AND "mass spectrometry") OR ("metabolo*" AND "NMR"))) OR (("genomic*" OR "genom* sequencing") AND ("proteomic*" OR ("proteo*" AND "mass spectrometry"))) OR (("transcriptomic*" OR "transcriptom* sequencing" OR "RNA seq*") AND ("epigenomic*" OR "epigenom* sequencing")) OR (("transcriptomic*" OR "transcriptom* sequencing" OR "RNA seq*") AND ("metabolomic*" OR ("metabolo*" AND "mass spectrometry") OR ("metabolo*" AND "NMR"))) OR (("transcriptomic*" OR "transcriptom* sequencing" OR "RNA seq*") AND ("proteomic*" OR ("proteo*" AND "mass spectrometry"))) OR (("epigenomic*" OR "epigenom* sequencing") AND ("metabolomic*" OR ("metabolo*" AND "mass spectrometry") OR ("metabolo*" AND "NMR"))) OR (("epigenomic*" OR "epigenom* sequencing") AND ("proteomic*" OR ("proteo*" AND "mass spectrometry"))) OR (("metabolomic*" OR ("metabolo*" AND "mass spectrometry") OR ("metabolo*" AND "NMR")) AND ("proteomic*" OR ("proteo*" AND "mass spectrometry"))) OR (("genomic*" OR "genom* sequencing") AND "phosphoproteomic*") OR (("genomic*" OR "genom* sequencing") AND "epitranscriptomic*") OR (("genomic*" OR "genom* sequencing") AND "lipidomic*") OR (("genomic*" OR "genom* sequencing") AND "glycomic*") OR (("genomic*" OR "genom* sequencing") AND "pharmacogenomic*") OR (("genomic*" OR	115

"genom* sequencing") AND "fluxomic*") OR (("genomic*" OR "genom* sequencing") AND "interactomic*") OR (("transcriptomic*" OR "transcriptom* sequencing" OR "RNA seq*") AND "phosphoproteomic*") OR (("transcriptomic*" OR "transcriptom* sequencing" OR "RNA seq*") AND "epitranscriptomic*") OR (("transcriptomic*" OR "transcriptom* sequencing" OR "RNA seq*") AND "lipidomic*") OR (("transcriptomic*" OR "transcriptom* sequencing" OR "RNA seq*") AND "glycomic*") OR (("transcriptomic*" OR "transcriptom* sequencing" OR "RNA seq*") AND "pharmacogenomic*") OR (("transcriptomic*" OR "transcriptom* sequencing" OR "RNA seq*") AND "fluxomic*") OR (("transcriptomic*" OR "transcriptom* sequencing" OR "RNA seq*") AND "interactomic*") OR (("epigenomic*" OR "epigenom* sequencing") AND "phosphoproteomic*") OR (("epigenomic*" OR "epigenom* sequencing") AND "epitranscriptomic*") OR (("epigenomic*" OR "epigenom* sequencing") AND "lipidomic*") OR (("epigenomic*" OR "epigenom* sequencing") AND "glycomic*") OR (("epigenomic*" OR "epigenom* sequencing") AND "pharmacogenomic*") OR (("epigenomic*" OR "epigenom* sequencing") AND "fluxomic*") OR (("epigenomic*" OR "epigenom* sequencing") AND "interactomic*") OR (("metabolomic*" OR ("metabolo*" AND "mass spectrometry") OR ("metabolo*" AND "NMR")) AND "phosphoproteomic*") OR (("metabolomic*" OR ("metabolo*" AND "mass spectrometry") OR ("metabolo*" AND "NMR")) AND "epitranscriptomic*") OR (("metabolomic*" OR ("metabolo*" AND "mass spectrometry") OR ("metabolo*" AND "NMR")) AND "lipidomic*") OR (("metabolomic*" OR ("metabolo*" AND "mass spectrometry") OR ("metabolo*" AND "NMR")) AND "glycomic*") OR (("metabolomic*" OR ("metabolo*" AND "mass spectrometry") OR ("metabolo*" AND "NMR")) AND "pharmacogenomic*") OR (("metabolomic*" OR ("metabolo*" AND "mass spectrometry") OR ("metabolo*" AND "NMR")) AND "fluxomic*") OR (("metabolomic*" OR ("metabolo*" AND "mass spectrometry") OR ("metabolo*" AND "NMR")) AND "interactomic*") OR (("proteomic*" OR ("proteo*" AND "mass spectrometry")) AND "phosphoproteomic*") OR (("proteomic*" OR ("proteo*" AND "mass spectrometry")) AND "epitranscriptomic*") OR (("proteomic*" OR ("proteo*" AND "mass spectrometry")) AND "lipidomic*") OR (("proteomic*" OR ("proteo*" AND "mass spectrometry")) AND "glycomic*") OR (("proteomic*" OR ("proteo*" AND "mass spectrometry")) AND "pharmacogenomic*") OR (("proteomic*" OR ("proteo*" AND "mass spectrometry")) AND "fluxomic*") OR (("proteomic*" OR ("proteo*" AND "mass spectrometry")) AND "interactomic*"))) OR ("multiomic*" OR "multi-omic*" OR "crossomic*" OR "cross-omic*" OR "cross omic*" OR "panomic*" OR "pan-omic*" OR "transomic*" OR "trans-omic*" OR "multiple omic*")))	
TS=((("Human Embryonic Kidney" OR "HEK293*" OR "HEK 293*") AND "cell*" NOT ("Chinese Hamster Ovary" OR "CHO cell*" OR "CHO-K1" OR "CHO-S" OR "CHO-DG44" OR "CHO-DUK" OR "CHOZN" OR "CHO-T" OR "CHO-Lec*" OR "CHO-H" OR "CHO-AT3-2" OR "CHO-C" OR "CHO-M" OR "CHO-AI") AND "cell*") AND ((((("genomic*" OR	161

"genom* sequencing") AND ("transcriptomic*" OR "transcriptom* sequencing" OR "RNA seq*")) OR (("genomic*" OR "genom* sequencing") AND ("epigenomic*" OR "epigenom* sequencing")) OR (("genomic*" OR "genom* sequencing") AND ("metabolomic*" OR ("metabolo*" AND "mass spectrometry") OR ("metabolo*" AND "NMR"))) OR (("genomic*" OR "genom* sequencing") AND ("proteomic*" OR ("proteo*" AND "mass spectrometry"))) OR (("transcriptomic*" OR "transcriptom* sequencing" OR "RNA seq*") AND ("epigenomic*" OR "epigenom* sequencing")) OR (("transcriptomic*" OR "transcriptom* sequencing" OR "RNA seq*") AND ("metabolomic*" OR ("metabolo*" AND "mass spectrometry") OR ("metabolo*" AND "NMR"))) OR (("transcriptomic*" OR "transcriptom* sequencing" OR "RNA seq*") AND ("proteomic*" OR ("proteo*" AND "mass spectrometry"))) OR (("epigenomic*" OR "epigenom* sequencing") AND ("metabolomic*" OR ("metabolo*" AND "mass spectrometry") OR ("metabolo*" AND "NMR"))) OR (("epigenomic*" OR "epigenom* sequencing") AND ("proteomic*" OR ("proteo*" AND "mass spectrometry"))) OR (("metabolomic*" OR ("metabolo*" AND "mass spectrometry") OR ("metabolo*" AND "NMR")) AND ("proteomic*" OR ("proteo*" AND "mass spectrometry"))) OR (("genomic*" OR "genom* sequencing") AND "phosphoproteomic*") OR (("genomic*" OR "genom* sequencing") AND "epitranscriptomic*") OR (("genomic*" OR "genom* sequencing") AND "lipidomic*") OR (("genomic*" OR "genom* sequencing") AND "glycomic*") OR (("genomic*" OR "genom* sequencing") AND "pharmacogenomic*") OR (("genomic*" OR "genom* sequencing") AND "fluxomic*") OR (("genomic*" OR "genom* sequencing") AND "interactomic*") OR (("transcriptomic*" OR "transcriptom* sequencing" OR "RNA seq*") AND "phosphoproteomic*") OR (("transcriptomic*" OR "transcriptom* sequencing" OR "RNA seq*") AND "epitranscriptomic*") OR (("transcriptomic*" OR "transcriptom* sequencing" OR "RNA seq*") AND "lipidomic*") OR (("transcriptomic*" OR "transcriptom* sequencing" OR "RNA seq*") AND "glycomic*") OR (("transcriptomic*" OR "transcriptom* sequencing" OR "RNA seq*") AND "pharmacogenomic*") OR (("transcriptomic*" OR "transcriptom* sequencing" OR "RNA seq*") AND "fluxomic*") OR (("transcriptomic*" OR "transcriptom* sequencing" OR "RNA seq*") AND "interactomic*") OR (("epigenomic*" OR "epigenom* sequencing") AND "phosphoproteomic*") OR (("epigenomic*" OR "epigenom* sequencing") AND "epitranscriptomic*") OR (("epigenomic*" OR "epigenom* sequencing") AND "lipidomic*") OR (("epigenomic*" OR "epigenom* sequencing") AND "glycomic*") OR (("epigenomic*" OR "epigenom* sequencing") AND "pharmacogenomic*") OR (("epigenomic*" OR "epigenom* sequencing") AND "fluxomic*") OR (("epigenomic*" OR "epigenom* sequencing") AND "interactomic*") OR (("metabolomic*" OR ("metabolo*" AND "mass spectrometry") OR ("metabolo*" AND "NMR")) AND "phosphoproteomic*") OR (("metabolomic*" OR ("metabolo*" AND "mass spectrometry") OR ("metabolo*" AND "NMR")) AND "epitranscriptomic*") OR (("metabolomic*" OR ("metabolo*" AND "mass spectrometry") OR	

("metabolo*" AND "NMR")) AND "lipidomic*") OR (("metabolomic*" OR ("metabolo*" AND "mass spectrometry") OR ("metabolo*" AND "NMR")) AND "glycomic*") OR (("metabolomic*" OR ("metabolo*" AND "mass spectrometry") OR ("metabolo*" AND "NMR")) AND "pharmacogenomic*") OR (("metabolomic*" OR ("metabolo*" AND "mass spectrometry") OR ("metabolo*" AND "NMR")) AND "fluxomic*") OR (("metabolomic*" OR ("metabolo*" AND "mass spectrometry") OR ("metabolo*" AND "NMR")) AND "interactomic*") OR (("proteomic*" OR ("proteo*" AND "mass spectrometry")) AND "phosphoproteomic*") OR (("proteomic*" OR ("proteo*" AND "mass spectrometry")) AND "epitranscriptomic*") OR (("proteomic*" OR ("proteo*" AND "mass spectrometry")) AND "lipidomic*") OR (("proteomic*" OR ("proteo*" AND "mass spectrometry")) AND "glycomic*") OR (("proteomic*" OR ("proteo*" AND "mass spectrometry")) AND "pharmacogenomic*") OR (("proteomic*" OR ("proteo*" AND "mass spectrometry")) AND "fluxomic*") OR (("proteomic*" OR ("proteo*" AND "mass spectrometry")) AND "interactomic*")))))	
TS=((("Human Embryonic Kidney" OR "HEK293*" OR "HEK 293*") AND "cell*" NOT ("Chinese Hamster Ovary" OR "CHO cell*" OR "CHO-K1" OR "CHO-S" OR "CHO-DG44" OR "CHO-DUK" OR "CHOZN" OR "CHO-T" OR "CHO-Lec*" OR "CHO-H" OR "CHO-AT3-2" OR "CHO-C" OR "CHO-M" OR "CHO-AI") AND "cell*") AND ((((("genomic*" OR "genom* sequencing") AND ("transcriptomic*" OR "transcriptom* sequencing" OR "RNA seq*")) OR (("genomic*" OR "genom* sequencing") AND ("epigenomic*" OR "epigenom* sequencing")) OR (("genomic*" OR "genom* sequencing") AND ("metabolomic*" OR ("metabolo*" AND "mass spectrometry") OR ("metabolo*" AND "NMR"))) OR (("genomic*" OR "genom* sequencing") AND ("proteomic*" OR ("proteo*" AND "mass spectrometry"))) OR (("transcriptomic*" OR "transcriptom* sequencing" OR "RNA seq*") AND ("epigenomic*" OR "epigenom* sequencing")) OR (("transcriptomic*" OR "transcriptom* sequencing" OR "RNA seq*") AND ("metabolomic*" OR ("metabolo*" AND "mass spectrometry") OR ("metabolo*" AND "NMR"))) OR (("transcriptomic*" OR "transcriptom* sequencing" OR "RNA seq*") AND ("proteomic*" OR ("proteo*" AND "mass spectrometry"))) OR (("epigenomic*" OR "epigenom* sequencing") AND ("metabolomic*" OR ("metabolo*" AND "mass spectrometry") OR ("metabolo*" AND "NMR"))) OR (("epigenomic*" OR "epigenom* sequencing") AND ("proteomic*" OR ("proteo*" AND "mass spectrometry"))) OR (("metabolomic*" OR ("metabolo*" AND "mass spectrometry") OR ("metabolo*" AND "NMR")) AND ("proteomic*" OR ("proteo*" AND "mass spectrometry"))) OR (("genomic*" OR "genom* sequencing") AND "phosphoproteomic*") OR (("genomic*" OR "genom* sequencing") AND "epitranscriptomic*") OR (("genomic*" OR "genom* sequencing") AND "lipidomic*") OR (("genomic*" OR "genom* sequencing") AND "glycomic*") OR (("genomic*" OR "genom* sequencing") AND "pharmacogenomic*") OR (("genomic*" OR "genom* sequencing") AND "fluxomic*") OR (("genomic*" OR "genom* sequencing") AND "interactomic*") OR (("transcriptomic*" OR "transcriptom* sequencing" OR "RNA seq*") AND	7

"phosphoproteomic*") OR (("transcriptomic*" OR "transcriptom* sequencing" OR "RNA seq*") AND "epitranscriptomic*") OR (("transcriptomic*" OR "transcriptom* sequencing" OR "RNA seq*") AND "lipidomic*") OR (("transcriptomic*" OR "transcriptom* sequencing" OR "RNA seq*") AND "glycomic*") OR (("transcriptomic*" OR "transcriptom* sequencing" OR "RNA seq*") AND "pharmacogenomic*") OR (("transcriptomic*" OR "transcriptom* sequencing" OR "RNA seq*") AND "fluxomic*") OR (("transcriptomic*" OR "transcriptom* sequencing" OR "RNA seq*") AND "interactomic*") OR (("epigenomic*" OR "epigenom* sequencing") AND "phosphoproteomic*") OR (("epigenomic*" OR "epigenom* sequencing") AND "epitranscriptomic*") OR (("epigenomic*" OR "epigenom* sequencing") AND "lipidomic*") OR (("epigenomic*" OR "epigenom* sequencing") AND "glycomic*") OR (("epigenomic*" OR "epigenom* sequencing") AND "pharmacogenomic*") OR (("epigenomic*" OR "epigenom* sequencing") AND "fluxomic*") OR (("epigenomic*" OR "epigenom* sequencing") AND "interactomic*") OR (("metabolomic*" OR ("metabolo*" AND "mass spectrometry") OR ("metabolo*" AND "NMR")) AND "phosphoproteomic*") OR (("metabolomic*" OR ("metabolo*" AND "mass spectrometry") OR ("metabolo*" AND "NMR")) AND "epitranscriptomic*") OR (("metabolomic*" OR ("metabolo*" AND "mass spectrometry") OR ("metabolo*" AND "NMR")) AND "lipidomic*") OR (("metabolomic*" OR ("metabolo*" AND "mass spectrometry") OR ("metabolo*" AND "NMR")) AND "glycomic*") OR (("metabolomic*" OR ("metabolo*" AND "mass spectrometry") OR ("metabolo*" AND "NMR")) AND "pharmacogenomic*") OR (("metabolomic*" OR ("metabolo*" AND "mass spectrometry") OR ("metabolo*" AND "NMR")) AND "fluxomic*") OR (("metabolomic*" OR ("metabolo*" AND "mass spectrometry") OR ("metabolo*" AND "NMR")) AND "interactomic*") OR (("proteomic*" OR ("proteo*" AND "mass spectrometry")) AND "phosphoproteomic*") OR (("proteomic*" OR ("proteo*" AND "mass spectrometry")) AND "epitranscriptomic*") OR (("proteomic*" OR ("proteo*" AND "mass spectrometry")) AND "lipidomic*") OR (("proteomic*" OR ("proteo*" AND "mass spectrometry")) AND "glycomic*") OR (("proteomic*" OR ("proteo*" AND "mass spectrometry")) AND "pharmacogenomic*") OR (("proteomic*" OR ("proteo*" AND "mass spectrometry")) AND "fluxomic*") OR (("proteomic*" OR ("proteo*" AND "mass spectrometry")) AND "interactomic*")))) AND ("multiomic*" OR "multi-omic*" OR "crossomic*" OR "cross-omic*" OR "cross omic*" OR "panomic*" OR "pan-omic*" OR "transomic*" OR "trans-omic*" OR "multiple omic*"))	
TS=((("Human Embryonic Kidney" OR "HEK293*" OR "HEK 293*") AND "cell*" NOT ("Chinese Hamster Ovary" OR "CHO cell*" OR "CHO-K1" OR "CHO-S" OR "CHO-DG44" OR "CHO-DUK" OR "CHOZN" OR "CHO-T" OR "CHO-Lec*" OR "CHO-H" OR "CHO-AT3-2" OR "CHO-C" OR "CHO-M" OR "CHO-AI") AND "cell*") AND ((((("genomic*" OR "genom* sequencing") AND ("transcriptomic*" OR "transcriptom* sequencing" OR "RNA seq*")) OR (("genomic*" OR "genom* sequencing") AND ("epigenomic*" OR "epigenom* sequencing")) OR	170

(("genomic*" OR "genom* sequencing") AND ("metabolomic*" OR ("metabolo*" AND "mass spectrometry") OR ("metabolo*" AND "NMR"))) OR (("genomic*" OR "genom* sequencing") AND ("proteomic*" OR ("proteo*" AND "mass spectrometry"))) OR (("transcriptomic*" OR "transcriptom* sequencing" OR "RNA seq*") AND ("epigenomic*" OR "epigenom* sequencing")) OR (("transcriptomic*" OR "transcriptom* sequencing" OR "RNA seq*") AND ("metabolomic*" OR ("metabolo*" AND "mass spectrometry") OR ("metabolo*" AND "NMR"))) OR (("transcriptomic*" OR "transcriptom* sequencing" OR "RNA seq*") AND ("proteomic*" OR ("proteo*" AND "mass spectrometry"))) OR (("epigenomic*" OR "epigenom* sequencing") AND ("metabolomic*" OR ("metabolo*" AND "mass spectrometry") OR ("metabolo*" AND "NMR"))) OR (("epigenomic*" OR "epigenom* sequencing") AND ("proteomic*" OR ("proteo*" AND "mass spectrometry"))) OR (("metabolomic*" OR ("metabolo*" AND "mass spectrometry") OR ("metabolo*" AND "NMR")) AND ("proteomic*" OR ("proteo*" AND "mass spectrometry"))) OR (("genomic*" OR "genom* sequencing") AND "phosphoproteomic*") OR (("genomic*" OR "genom* sequencing") AND "epitranscriptomic*") OR (("genomic*" OR "genom* sequencing") AND "lipidomic*") OR (("genomic*" OR "genom* sequencing") AND "glycomic*") OR (("genomic*" OR "genom* sequencing") AND "pharmacogenomic*") OR (("genomic*" OR "genom* sequencing") AND "fluxomic*") OR (("genomic*" OR "genom* sequencing") AND "interactomic*") OR (("transcriptomic*" OR "transcriptom* sequencing" OR "RNA seq*") AND "phosphoproteomic*") OR (("transcriptomic*" OR "transcriptom* sequencing" OR "RNA seq*") AND "epitranscriptomic*") OR (("transcriptomic*" OR "transcriptom* sequencing" OR "RNA seq*") AND "lipidomic*") OR (("transcriptomic*" OR "transcriptom* sequencing" OR "RNA seq*") AND "glycomic*") OR (("transcriptomic*" OR "transcriptom* sequencing" OR "RNA seq*") AND "pharmacogenomic*") OR (("transcriptomic*" OR "transcriptom* sequencing" OR "RNA seq*") AND "fluxomic*") OR (("transcriptomic*" OR "transcriptom* sequencing" OR "RNA seq*") AND "interactomic*") OR (("epigenomic*" OR "epigenom* sequencing") AND "phosphoproteomic*") OR (("epigenomic*" OR "epigenom* sequencing") AND "epitranscriptomic*") OR (("epigenomic*" OR "epigenom* sequencing") AND "lipidomic*") OR (("epigenomic*" OR "epigenom* sequencing") AND "glycomic*") OR (("epigenomic*" OR "epigenom* sequencing") AND "pharmacogenomic*") OR (("epigenomic*" OR "epigenom* sequencing") AND "fluxomic*") OR (("epigenomic*" OR "epigenom* sequencing") AND "interactomic*") OR (("metabolomic*" OR ("metabolo*" AND "mass spectrometry") OR ("metabolo*" AND "NMR")) AND "phosphoproteomic*") OR (("metabolomic*" OR ("metabolo*" AND "mass spectrometry") OR ("metabolo*" AND "NMR")) AND "epitranscriptomic*") OR (("metabolomic*" OR ("metabolo*" AND "mass spectrometry") OR ("metabolo*" AND "NMR")) AND "lipidomic*") OR (("metabolomic*" OR ("metabolo*" AND "mass spectrometry") OR ("metabolo*" AND "NMR")) AND "glycomic*") OR (("metabolomic*" OR ("metabolo*" AND	

"mass spectrometry") OR ("metabolo*" AND "NMR")) AND "pharmacogenomic*") OR (("metabolomic*" OR ("metabolo*" AND "mass spectrometry") OR ("metabolo*" AND "NMR")) AND "fluxomic*") OR (("metabolomic*" OR ("metabolo*" AND "mass spectrometry") OR ("metabolo*" AND "NMR")) AND "interactomic*") OR (("proteomic*" OR ("proteo*" AND "mass spectrometry")) AND "phosphoproteomic*") OR (("proteomic*" OR ("proteo*" AND "mass spectrometry")) AND "epitranscriptomic*") OR (("proteomic*" OR ("proteo*" AND "mass spectrometry")) AND "lipidomic*") OR (("proteomic*" OR ("proteo*" AND "mass spectrometry")) AND "glycomic*") OR (("proteomic*" OR ("proteo*" AND "mass spectrometry")) AND "pharmacogenomic*") OR (("proteomic*" OR ("proteo*" AND "mass spectrometry")) AND "fluxomic*") OR (("proteomic*" OR ("proteo*" AND "mass spectrometry")) AND "interactomic*"))) OR ("multiomic*" OR "multi-omic*" OR "crossomic*" OR "cross-omic*" OR "cross omic*" OR "panomic*" OR "pan-omic*" OR "transomic*" OR "trans-omic*" OR "multiple omic*")))	
TS=((("Chinese Hamster Ovary" OR "CHO cell*" OR "CHO-K1" OR "CHO-S" OR "CHO-DG44" OR "CHO-DUK" OR "CHOZN" OR "CHO-T" OR "CHO-Lec*" OR "CHO-H" OR "CHO-AT3-2" OR "CHO-C" OR "CHO-M" OR "CHO-AI") AND "cell*" AND ("Human Embryonic Kidney" OR "HEK293*" OR "HEK 293*") AND "cell*") AND ((((("genomic*" OR "genom* sequencing") AND ("transcriptomic*" OR "transcriptom* sequencing" OR "RNA seq*")) OR (("genomic*" OR "genom* sequencing") AND ("epigenomic*" OR "epigenom* sequencing")) OR (("genomic*" OR "genom* sequencing") AND ("metabolomic*" OR ("metabolo*" AND "mass spectrometry") OR ("metabolo*" AND "NMR"))) OR (("genomic*" OR "genom* sequencing") AND ("proteomic*" OR ("proteo*" AND "mass spectrometry"))) OR (("transcriptomic*" OR "transcriptom* sequencing" OR "RNA seq*") AND ("epigenomic*" OR "epigenom* sequencing")) OR (("transcriptomic*" OR "transcriptom* sequencing" OR "RNA seq*") AND ("metabolomic*" OR ("metabolo*" AND "mass spectrometry") OR ("metabolo*" AND "NMR"))) OR (("transcriptomic*" OR "transcriptom* sequencing" OR "RNA seq*") AND ("proteomic*" OR ("proteo*" AND "mass spectrometry"))) OR (("epigenomic*" OR "epigenom* sequencing") AND ("metabolomic*" OR ("metabolo*" AND "mass spectrometry") OR ("metabolo*" AND "NMR"))) OR (("epigenomic*" OR "epigenom* sequencing") AND ("proteomic*" OR ("proteo*" AND "mass spectrometry"))) OR (("metabolomic*" OR ("metabolo*" AND "mass spectrometry") OR ("metabolo*" AND "NMR")) AND ("proteomic*" OR ("proteo*" AND "mass spectrometry"))) OR (("genomic*" OR "genom* sequencing") AND "phosphoproteomic*") OR (("genomic*" OR "genom* sequencing") AND "epitranscriptomic*") OR (("genomic*" OR "genom* sequencing") AND "lipidomic*") OR (("genomic*" OR "genom* sequencing") AND "glycomic*") OR (("genomic*" OR "genom* sequencing") AND "pharmacogenomic*") OR (("genomic*" OR "genom* sequencing") AND "fluxomic*") OR (("genomic*" OR "genom* sequencing") AND "interactomic*") OR (("transcriptomic*" OR "transcriptom* sequencing" OR "RNA seq*") AND	6

"phosphoproteomic*") OR (("transcriptomic*" OR "transcriptom* sequencing" OR "RNA seq*") AND "epitranscriptomic*") OR (("transcriptomic*" OR "transcriptom* sequencing" OR "RNA seq*") AND "lipidomic*") OR (("transcriptomic*" OR "transcriptom* sequencing" OR "RNA seq*") AND "glycomic*") OR (("transcriptomic*" OR "transcriptom* sequencing" OR "RNA seq*") AND "pharmacogenomic*") OR (("transcriptomic*" OR "transcriptom* sequencing" OR "RNA seq*") AND "fluxomic*") OR (("transcriptomic*" OR "transcriptom* sequencing" OR "RNA seq*") AND "interactomic*") OR (("epigenomic*" OR "epigenom* sequencing") AND "phosphoproteomic*") OR (("epigenomic*" OR "epigenom* sequencing") AND "epitranscriptomic*") OR (("epigenomic*" OR "epigenom* sequencing") AND "lipidomic*") OR (("epigenomic*" OR "epigenom* sequencing") AND "glycomic*") OR (("epigenomic*" OR "epigenom* sequencing") AND "pharmacogenomic*") OR (("epigenomic*" OR "epigenom* sequencing") AND "fluxomic*") OR (("epigenomic*" OR "epigenom* sequencing") AND "interactomic*") OR (("metabolomic*" OR ("metabolo*" AND "mass spectrometry") OR ("metabolo*" AND "NMR")) AND "phosphoproteomic*") OR (("metabolomic*" OR ("metabolo*" AND "mass spectrometry") OR ("metabolo*" AND "NMR")) AND "epitranscriptomic*") OR (("metabolomic*" OR ("metabolo*" AND "mass spectrometry") OR ("metabolo*" AND "NMR")) AND "lipidomic*") OR (("metabolomic*" OR ("metabolo*" AND "mass spectrometry") OR ("metabolo*" AND "NMR")) AND "glycomic*") OR (("metabolomic*" OR ("metabolo*" AND "mass spectrometry") OR ("metabolo*" AND "NMR")) AND "pharmacogenomic*") OR (("metabolomic*" OR ("metabolo*" AND "mass spectrometry") OR ("metabolo*" AND "NMR")) AND "fluxomic*") OR (("metabolomic*" OR ("metabolo*" AND "mass spectrometry") OR ("metabolo*" AND "NMR")) AND "interactomic*") OR (("proteomic*" OR ("proteo*" AND "mass spectrometry")) AND "phosphoproteomic*") OR (("proteomic*" OR ("proteo*" AND "mass spectrometry")) AND "epitranscriptomic*") OR (("proteomic*" OR ("proteo*" AND "mass spectrometry")) AND "lipidomic*") OR (("proteomic*" OR ("proteo*" AND "mass spectrometry")) AND "glycomic*") OR (("proteomic*" OR ("proteo*" AND "mass spectrometry")) AND "pharmacogenomic*") OR (("proteomic*" OR ("proteo*" AND "mass spectrometry")) AND "fluxomic*") OR (("proteomic*" OR ("proteo*" AND "mass spectrometry")) AND "interactomic*")))))	
TS=((("Chinese Hamster Ovary" OR "CHO cell*" OR "CHO-K1" OR "CHO-S" OR "CHO-DG44" OR "CHO-DUK" OR "CHOZN" OR "CHO-T" OR "CHO-Lec*" OR "CHO-H" OR "CHO-AT3-2" OR "CHO-C" OR "CHO-M" OR "CHO-AI") AND "cell*" AND ("Human Embryonic Kidney" OR "HEK293*" OR "HEK 293*") AND "cell*") AND ((((("genomic*" OR "genom* sequencing") AND ("transcriptomic*" OR "transcriptom* sequencing" OR "RNA seq*")) OR (("genomic*" OR "genom* sequencing") AND ("epigenomic*" OR "epigenom* sequencing")) OR (("genomic*" OR "genom* sequencing") AND ("metabolomic*" OR ("metabolo*" AND "mass spectrometry") OR ("metabolo*" AND "NMR"))) OR (("genomic*" OR "genom* sequencing") AND	1

("proteomic*" OR ("proteo*" AND "mass spectrometry"))) OR (("transcriptomic*" OR "transcriptom* sequencing" OR "RNA seq*") AND ("epigenomic*" OR "epigenom* sequencing")) OR (("transcriptomic*" OR "transcriptom* sequencing" OR "RNA seq*") AND ("metabolomic*" OR ("metabolo*" AND "mass spectrometry") OR ("metabolo*" AND "NMR"))) OR (("transcriptomic*" OR "transcriptom* sequencing" OR "RNA seq*") AND ("proteomic*" OR ("proteo*" AND "mass spectrometry"))) OR (("epigenomic*" OR "epigenom* sequencing") AND ("metabolomic*" OR ("metabolo*" AND "mass spectrometry") OR ("metabolo*" AND "NMR"))) OR (("epigenomic*" OR "epigenom* sequencing") AND ("proteomic*" OR ("proteo*" AND "mass spectrometry"))) OR (("metabolomic*" OR ("metabolo*" AND "mass spectrometry") OR ("metabolo*" AND "NMR")) AND ("proteomic*" OR ("proteo*" AND "mass spectrometry"))) OR (("genomic*" OR "genom* sequencing") AND "phosphoproteomic*") OR (("genomic*" OR "genom* sequencing") AND "epitranscriptomic*") OR (("genomic*" OR "genom* sequencing") AND "lipidomic*") OR (("genomic*" OR "genom* sequencing") AND "glycomic*") OR (("genomic*" OR "genom* sequencing") AND "pharmacogenomic*") OR (("genomic*" OR "genom* sequencing") AND "fluxomic*") OR (("genomic*" OR "genom* sequencing") AND "interactomic*") OR (("transcriptomic*" OR "transcriptom* sequencing" OR "RNA seq*") AND "phosphoproteomic*") OR (("transcriptomic*" OR "transcriptom* sequencing" OR "RNA seq*") AND "epitranscriptomic*") OR (("transcriptomic*" OR "transcriptom* sequencing" OR "RNA seq*") AND "lipidomic*") OR (("transcriptomic*" OR "transcriptom* sequencing" OR "RNA seq*") AND "glycomic*") OR (("transcriptomic*" OR "transcriptom* sequencing" OR "RNA seq*") AND "pharmacogenomic*") OR (("transcriptomic*" OR "transcriptom* sequencing" OR "RNA seq*") AND "fluxomic*") OR (("transcriptomic*" OR "transcriptom* sequencing" OR "RNA seq*") AND "interactomic*") OR (("epigenomic*" OR "epigenom* sequencing") AND "phosphoproteomic*") OR (("epigenomic*" OR "epigenom* sequencing") AND "epitranscriptomic*") OR (("epigenomic*" OR "epigenom* sequencing") AND "lipidomic*") OR (("epigenomic*" OR "epigenom* sequencing") AND "glycomic*") OR (("epigenomic*" OR "epigenom* sequencing") AND "pharmacogenomic*") OR (("epigenomic*" OR "epigenom* sequencing") AND "fluxomic*") OR (("epigenomic*" OR "epigenom* sequencing") AND "interactomic*") OR (("metabolomic*" OR ("metabolo*" AND "mass spectrometry") OR ("metabolo*" AND "NMR")) AND "phosphoproteomic*") OR (("metabolomic*" OR ("metabolo*" AND "mass spectrometry") OR ("metabolo*" AND "NMR")) AND "epitranscriptomic*") OR (("metabolomic*" OR ("metabolo*" AND "mass spectrometry") OR ("metabolo*" AND "NMR")) AND "lipidomic*") OR (("metabolomic*" OR ("metabolo*" AND "mass spectrometry") OR ("metabolo*" AND "NMR")) AND "glycomic*") OR (("metabolomic*" OR ("metabolo*" AND "mass spectrometry") OR ("metabolo*" AND "NMR")) AND "pharmacogenomic*") OR (("metabolomic*" OR ("metabolo*" AND "mass spectrometry") OR ("metabolo*" AND "NMR")) AND "fluxomic*")	

OR (("metabolomic*" OR ("metabolo*" AND "mass spectrometry") OR ("metabolo*" AND "NMR")) AND "interactomic*") OR (("proteomic*" OR ("proteo*" AND "mass spectrometry")) AND "phosphoproteomic*") OR (("proteomic*" OR ("proteo*" AND "mass spectrometry")) AND "epitranscriptomic*") OR (("proteomic*" OR ("proteo*" AND "mass spectrometry")) AND "lipidomic*") OR (("proteomic*" OR ("proteo*" AND "mass spectrometry")) AND "glycomic*") OR (("proteomic*" OR ("proteo*" AND "mass spectrometry")) AND "pharmacogenomic*") OR (("proteomic*" OR ("proteo*" AND "mass spectrometry")) AND "fluxomic*") OR (("proteomic*" OR ("proteo*" AND "mass spectrometry")) AND "interactomic*")))) AND ("multiomic*" OR "multi-omic*" OR "crossomic*" OR "cross-omic*" OR "cross omic*" OR "panomic*" OR "pan-omic*" OR "transomic*" OR "trans-omic*" OR "multiple omic*"))	
TS=((("Chinese Hamster Ovary" OR "CHO cell*" OR "CHO-K1" OR "CHO-S" OR "CHO-DG44" OR "CHO-DUK" OR "CHOZN" OR "CHO-T" OR "CHO-Lec*" OR "CHO-H" OR "CHO-AT3-2" OR "CHO-C" OR "CHO-M" OR "CHO-AI") AND "cell*" AND ("Human Embryonic Kidney" OR "HEK293*" OR "HEK 293*") AND "cell*") AND ((((("genomic*" OR "genom* sequencing") AND ("transcriptomic*" OR "transcriptom* sequencing" OR "RNA seq*")) OR (("genomic*" OR "genom* sequencing") AND ("epigenomic*" OR "epigenom* sequencing")) OR (("genomic*" OR "genom* sequencing") AND ("metabolomic*" OR ("metabolo*" AND "mass spectrometry") OR ("metabolo*" AND "NMR"))) OR (("genomic*" OR "genom* sequencing") AND ("proteomic*" OR ("proteo*" AND "mass spectrometry"))) OR (("transcriptomic*" OR "transcriptom* sequencing" OR "RNA seq*") AND ("epigenomic*" OR "epigenom* sequencing")) OR (("transcriptomic*" OR "transcriptom* sequencing" OR "RNA seq*") AND ("metabolomic*" OR ("metabolo*" AND "mass spectrometry") OR ("metabolo*" AND "NMR"))) OR (("transcriptomic*" OR "transcriptom* sequencing" OR "RNA seq*") AND ("proteomic*" OR ("proteo*" AND "mass spectrometry"))) OR (("epigenomic*" OR "epigenom* sequencing") AND ("metabolomic*" OR ("metabolo*" AND "mass spectrometry") OR ("metabolo*" AND "NMR"))) OR (("epigenomic*" OR "epigenom* sequencing") AND ("proteomic*" OR ("proteo*" AND "mass spectrometry"))) OR (("metabolomic*" OR ("metabolo*" AND "mass spectrometry") OR ("metabolo*" AND "NMR")) AND ("proteomic*" OR ("proteo*" AND "mass spectrometry"))) OR (("genomic*" OR "genom* sequencing") AND "phosphoproteomic*") OR (("genomic*" OR "genom* sequencing") AND "epitranscriptomic*") OR (("genomic*" OR "genom* sequencing") AND "lipidomic*") OR (("genomic*" OR "genom* sequencing") AND "glycomic*") OR (("genomic*" OR "genom* sequencing") AND "pharmacogenomic*") OR (("genomic*" OR "genom* sequencing") AND "fluxomic*") OR (("genomic*" OR "genom* sequencing") AND "interactomic*") OR (("transcriptomic*" OR "transcriptom* sequencing" OR "RNA seq*") AND "phosphoproteomic*") OR (("transcriptomic*" OR "transcriptom* sequencing" OR "RNA seq*") AND "epitranscriptomic*") OR (("transcriptomic*" OR "transcriptom* sequencing" OR "RNA seq*")	7

AND "lipidomic*") OR (("transcriptomic*" OR "transcriptom* sequencing" OR "RNA seq*") AND "glycomic*") OR (("transcriptomic*" OR "transcriptom* sequencing" OR "RNA seq*") AND "pharmacogenomic*") OR (("transcriptomic*" OR "transcriptom* sequencing" OR "RNA seq*") AND "fluxomic*") OR (("transcriptomic*" OR "transcriptom* sequencing" OR "RNA seq*") AND "interactomic*") OR (("epigenomic*" OR "epigenom* sequencing") AND "phosphoproteomic*") OR (("epigenomic*" OR "epigenom* sequencing") AND "epitranscriptomic*") OR (("epigenomic*" OR "epigenom* sequencing") AND "lipidomic*") OR (("epigenomic*" OR "epigenom* sequencing") AND "glycomic*") OR (("epigenomic*" OR "epigenom* sequencing") AND "pharmacogenomic*") OR (("epigenomic*" OR "epigenom* sequencing") AND "fluxomic*") OR (("epigenomic*" OR "epigenom* sequencing") AND "interactomic*") OR (("metabolomic*" OR ("metabolo*" AND "mass spectrometry") OR ("metabolo*" AND "NMR")) AND "phosphoproteomic*") OR (("metabolomic*" OR ("metabolo*" AND "mass spectrometry") OR ("metabolo*" AND "NMR")) AND "epitranscriptomic*") OR (("metabolomic*" OR ("metabolo*" AND "mass spectrometry") OR ("metabolo*" AND "NMR")) AND "lipidomic*") OR (("metabolomic*" OR ("metabolo*" AND "mass spectrometry") OR ("metabolo*" AND "NMR")) AND "glycomic*") OR (("metabolomic*" OR ("metabolo*" AND "mass spectrometry") OR ("metabolo*" AND "NMR")) AND "pharmacogenomic*") OR (("metabolomic*" OR ("metabolo*" AND "mass spectrometry") OR ("metabolo*" AND "NMR")) AND "fluxomic*") OR (("metabolomic*" OR ("metabolo*" AND "mass spectrometry") OR ("metabolo*" AND "NMR")) AND "interactomic*") OR (("proteomic*" OR ("proteo*" AND "mass spectrometry")) AND "phosphoproteomic*") OR (("proteomic*" OR ("proteo*" AND "mass spectrometry")) AND "epitranscriptomic*") OR (("proteomic*" OR ("proteo*" AND "mass spectrometry")) AND "lipidomic*") OR (("proteomic*" OR ("proteo*" AND "mass spectrometry")) AND "glycomic*") OR (("proteomic*" OR ("proteo*" AND "mass spectrometry")) AND "pharmacogenomic*") OR (("proteomic*" OR ("proteo*" AND "mass spectrometry")) AND "fluxomic*") OR (("proteomic*" OR ("proteo*" AND "mass spectrometry")) AND "interactomic*"))) OR ("multiomic*" OR "multi-omic*" OR "crossomic*" OR "cross-omic*" OR "cross omic*" OR "panomic*" OR "pan-omic*" OR "transomic*" OR "trans-omic*" OR "multiple omic*")))	

Table S14 Search Query Cell lines, multi-omics and bioprocessing terms.

Search Query	Number of Publications
TS=((("Chinese Hamster Ovary" OR "CHO cell*" OR "CHO-K1" OR "CHO-S" OR "CHO-DG44" OR "CHO-DUK" OR "CHOZN" OR "CHO-T" OR "CHO-Lec*" OR "CHO-H" OR "CHO-AT3-2" OR "CHO-C" OR "CHO-M" OR "CHO-AI") AND "cell*" NOT	19

("Human Embryonic Kidney" OR "HEK293*" OR "HEK 293*") AND "cell*") AND (((("genomic*" OR "genom* sequencing") AND ("transcriptomic*" OR "transcriptom* sequencing" OR "RNA seq*")) OR (("genomic*" OR "genom* sequencing") AND ("epigenomic*" OR "epigenom* sequencing")) OR (("genomic*" OR "genom* sequencing") AND ("metabolomic*" OR ("metabolo*" AND "mass spectrometry") OR ("metabolo*" AND "NMR"))) OR (("genomic*" OR "genom* sequencing") AND ("proteomic*" OR ("proteo*" AND "mass spectrometry"))) OR (("transcriptomic*" OR "transcriptom* sequencing" OR "RNA seq*") AND ("epigenomic*" OR "epigenom* sequencing")) OR (("transcriptomic*" OR "transcriptom* sequencing" OR "RNA seq*") AND ("metabolomic*" OR ("metabolo*" AND "mass spectrometry") OR ("metabolo*" AND "NMR"))) OR (("transcriptomic*" OR "transcriptom* sequencing" OR "RNA seq*") AND ("proteomic*" OR ("proteo*" AND "mass spectrometry"))) OR (("epigenomic*" OR "epigenom* sequencing") AND ("metabolomic*" OR ("metabolo*" AND "mass spectrometry") OR ("metabolo*" AND "NMR"))) OR (("epigenomic*" OR "epigenom* sequencing") AND ("proteomic*" OR ("proteo*" AND "mass spectrometry"))) OR (("metabolomic*" OR ("metabolo*" AND "mass spectrometry") OR ("metabolo*" AND "NMR")) AND ("proteomic*" OR ("proteo*" AND "mass spectrometry"))) OR (("genomic*" OR "genom* sequencing") AND "phosphoproteomic*") OR (("genomic*" OR "genom* sequencing") AND "epitranscriptomic*") OR (("genomic*" OR "genom* sequencing") AND "lipidomic*") OR (("genomic*" OR "genom* sequencing") AND "glycomic*") OR (("genomic*" OR "genom* sequencing") AND "pharmacogenomic*") OR (("genomic*" OR "genom* sequencing") AND "fluxomic*") OR (("genomic*" OR "genom* sequencing") AND "interactomic*") OR (("transcriptomic*" OR "transcriptom* sequencing" OR "RNA seq*") AND "phosphoproteomic*") OR (("transcriptomic*" OR "transcriptom* sequencing" OR "RNA seq*") AND "epitranscriptomic*") OR (("transcriptomic*" OR "transcriptom* sequencing" OR "RNA seq*") AND "lipidomic*") OR (("transcriptomic*" OR "transcriptom* sequencing" OR "RNA seq*") AND "glycomic*") OR (("transcriptomic*" OR "transcriptom* sequencing" OR "RNA seq*") AND "pharmacogenomic*") OR (("transcriptomic*" OR "transcriptom* sequencing" OR "RNA seq*") AND "fluxomic*") OR (("transcriptomic*" OR "transcriptom* sequencing" OR "RNA seq*") AND "interactomic*") OR (("epigenomic*" OR "epigenom* sequencing") AND "phosphoproteomic*") OR (("epigenomic*" OR "epigenom* sequencing") AND "epitranscriptomic*") OR (("epigenomic*" OR "epigenom* sequencing") AND "lipidomic*") OR (("epigenomic*" OR "epigenom* sequencing") AND "glycomic*") OR (("epigenomic*" OR "epigenom* sequencing") AND "pharmacogenomic*") OR (("epigenomic*" OR "epigenom* sequencing") AND "fluxomic*") OR (("epigenomic*" OR	

"epigenom* sequencing") AND "interactomic*") OR (("metabolomic*" OR ("metabolo*" AND "mass spectrometry") OR ("metabolo*" AND "NMR")) AND "phosphoproteomic*") OR (("metabolomic*" OR ("metabolo*" AND "mass spectrometry") OR ("metabolo*" AND "NMR")) AND "epitranscriptomic*") OR (("metabolomic*" OR ("metabolo*" AND "mass spectrometry") OR ("metabolo*" AND "NMR")) AND "lipidomic*") OR (("metabolomic*" OR ("metabolo*" AND "mass spectrometry") OR ("metabolo*" AND "NMR")) AND "glycomic*") OR (("metabolomic*" OR ("metabolo*" AND "mass spectrometry") OR ("metabolo*" AND "NMR")) AND "pharmacogenomic*") OR (("metabolomic*" OR ("metabolo*" AND "mass spectrometry") OR ("metabolo*" AND "NMR")) AND "fluxomic*") OR (("metabolomic*" OR ("metabolo*" AND "mass spectrometry") OR ("metabolo*" AND "NMR")) AND "interactomic*") OR (("proteomic*" OR ("proteo*" AND "mass spectrometry")) AND "phosphoproteomic*") OR (("proteomic*" OR ("proteo*" AND "mass spectrometry")) AND "epitranscriptomic*") OR (("proteomic*" OR ("proteo*" AND "mass spectrometry")) AND "lipidomic*") OR (("proteomic*" OR ("proteo*" AND "mass spectrometry")) AND "glycomic*") OR (("proteomic*" OR ("proteo*" AND "mass spectrometry")) AND "pharmacogenomic*") OR (("proteomic*" OR ("proteo*" AND "mass spectrometry")) AND "fluxomic*") OR (("proteomic*" OR ("proteo*" AND "mass spectrometry")) AND "interactomic*"))) AND ("producer cell line" OR "immortalized cell line" OR "immortalised cell line" OR "stable cell line" OR "cell line development" OR "cell line engineering" OR "suspension culture" OR "adherent culture" OR "defined media" OR "serum-free media" OR "fed-batch" OR "semi-perfusion" OR "perfusion culture" OR "media optimization" OR "media optimisation" OR "media components" OR "bioprocess" OR "bioreactor" OR "upstream processing" OR "downstream processing" OR "process scale-up" OR "scale-up" OR "continuous bioprocessing"))	
TS=((("Chinese Hamster Ovary" OR "CHO cell*" OR "CHO-K1" OR "CHO-S" OR "CHO-DG44" OR "CHO-DUK" OR "CHOZN" OR "CHO-T" OR "CHO-Lec*" OR "CHO-H" OR "CHO-AT3-2" OR "CHO-C" OR "CHO-M" OR "CHO-AI") AND "cell*" NOT ("Human Embryonic Kidney" OR "HEK293*" OR "HEK 293*") AND "cell*") AND (((("genomic*" OR "genom* sequencing") AND ("transcriptomic*" OR "transcriptom* sequencing" OR "RNA seq*")) OR (("genomic*" OR "genom* sequencing") AND ("epigenomic*" OR "epigenom* sequencing")) OR (("genomic*" OR "genom* sequencing") AND ("metabolomic*" OR ("metabolo*" AND "mass spectrometry") OR ("metabolo*" AND "NMR"))) OR (("genomic*" OR "genom* sequencing") AND ("proteomic*" OR ("proteo*" AND "mass spectrometry"))) OR (("transcriptomic*" OR "transcriptom* sequencing" OR "RNA seq*") AND ("epigenomic*" OR "epigenom* sequencing")) OR	7

(("transcriptomic*" OR "transcriptom* sequencing" OR "RNA seq*") AND ("metabolomic*" OR ("metabolo*" AND "mass spectrometry") OR ("metabolo*" AND "NMR"))) OR (("transcriptomic*" OR "transcriptom* sequencing" OR "RNA seq*") AND ("proteomic*" OR ("proteo*" AND "mass spectrometry"))) OR (("epigenomic*" OR "epigenom* sequencing") AND ("metabolomic*" OR ("metabolo*" AND "mass spectrometry") OR ("metabolo*" AND "NMR"))) OR (("epigenomic*" OR "epigenom* sequencing") AND ("proteomic*" OR ("proteo*" AND "mass spectrometry"))) OR (("metabolomic*" OR ("metabolo*" AND "mass spectrometry") OR ("metabolo*" AND "NMR")) AND ("proteomic*" OR ("proteo*" AND "mass spectrometry"))) OR (("genomic*" OR "genom* sequencing") AND "phosphoproteomic*") OR (("genomic*" OR "genom* sequencing") AND "epitranscriptomic*") OR (("genomic*" OR "genom* sequencing") AND "lipidomic*") OR (("genomic*" OR "genom* sequencing") AND "glycomic*") OR (("genomic*" OR "genom* sequencing") AND "pharmacogenomic*") OR (("genomic*" OR "genom* sequencing") AND "fluxomic*") OR (("genomic*" OR "genom* sequencing") AND "interactomic*") OR (("transcriptomic*" OR "transcriptom* sequencing" OR "RNA seq*") AND "phosphoproteomic*") OR (("transcriptomic*" OR "transcriptom* sequencing" OR "RNA seq*") AND "epitranscriptomic*") OR (("transcriptomic*" OR "transcriptom* sequencing" OR "RNA seq*") AND "lipidomic*") OR (("transcriptomic*" OR "transcriptom* sequencing" OR "RNA seq*") AND "glycomic*") OR (("transcriptomic*" OR "transcriptom* sequencing" OR "RNA seq*") AND "pharmacogenomic*") OR (("transcriptomic*" OR "transcriptom* sequencing" OR "RNA seq*") AND "fluxomic*") OR (("transcriptomic*" OR "transcriptom* sequencing" OR "RNA seq*") AND "interactomic*") OR (("epigenomic*" OR "epigenom* sequencing") AND "phosphoproteomic*") OR (("epigenomic*" OR "epigenom* sequencing") AND "epitranscriptomic*") OR (("epigenomic*" OR "epigenom* sequencing") AND "lipidomic*") OR (("epigenomic*" OR "epigenom* sequencing") AND "glycomic*") OR (("epigenomic*" OR "epigenom* sequencing") AND "pharmacogenomic*") OR (("epigenomic*" OR "epigenom* sequencing") AND "fluxomic*") OR (("epigenomic*" OR "epigenom* sequencing") AND "interactomic*") OR (("metabolomic*" OR ("metabolo*" AND "mass spectrometry") OR ("metabolo*" AND "NMR")) AND "phosphoproteomic*") OR (("metabolomic*" OR ("metabolo*" AND "mass spectrometry") OR ("metabolo*" AND "NMR")) AND "epitranscriptomic*") OR (("metabolomic*" OR ("metabolo*" AND "mass spectrometry") OR ("metabolo*" AND "NMR")) AND "lipidomic*") OR (("metabolomic*" OR ("metabolo*" AND "mass spectrometry") OR ("metabolo*" AND "NMR")) AND "glycomic*") OR (("metabolomic*" OR ("metabolo*" AND "mass spectrometry") OR ("metabolo*" AND "NMR")) AND "pharmacogenomic*") OR	

(("metabolomic*" OR ("metabolo*" AND "mass spectrometry") OR ("metabolo*" AND "NMR")) AND "fluxomic*") OR (("metabolomic*" OR ("metabolo*" AND "mass spectrometry") OR ("metabolo*" AND "NMR")) AND "interactomic*") OR (("proteomic*" OR ("proteo*" AND "mass spectrometry")) AND "phosphoproteomic*") OR (("proteomic*" OR ("proteo*" AND "mass spectrometry")) AND "epitranscriptomic*") OR (("proteomic*" OR ("proteo*" AND "mass spectrometry")) AND "lipidomic*") OR (("proteomic*" OR ("proteo*" AND "mass spectrometry")) AND "glycomic*") OR (("proteomic*" OR ("proteo*" AND "mass spectrometry")) AND "pharmacogenomic*") OR (("proteomic*" OR ("proteo*" AND "mass spectrometry")) AND "fluxomic*") OR (("proteomic*" OR ("proteo*" AND "mass spectrometry")) AND "interactomic*"))) AND ("multiomic*" OR "multi-omic*" OR "crossomic*" OR "cross-omic*" OR "cross omic*" OR "panomic*" OR "pan-omic*" OR "transomic*" OR "trans-omic*" OR "multiple omic*") AND ("producer cell line" OR "immortalized cell line" OR "immortalised cell line" OR "stable cell line" OR "cell line development" OR "cell line engineering" OR "suspension culture" OR "adherent culture" OR "defined media" OR "serum-free media" OR "fed-batch" OR "semi-perfusion" OR "perfusion culture" OR "media optimization" OR "media optimisation" OR "media components" OR "bioprocess" OR "bioreactor" OR "upstream processing" OR "downstream processing" OR "process scale-up" OR "scale-up" OR "continuous bioprocessing"))	
TS=((("Chinese Hamster Ovary" OR "CHO cell*" OR "CHO-K1" OR "CHO-S" OR "CHO-DG44" OR "CHO-DUK" OR "CHOZN" OR "CHO-T" OR "CHO-Lec*" OR "CHO-H" OR "CHO-AT3-2" OR "CHO-C" OR "CHO-M" OR "CHO-AI") AND "cell*" NOT ("Human Embryonic Kidney" OR "HEK293*" OR "HEK 293*") AND "cell*") AND ((((("genomic*" OR "genom* sequencing") AND ("transcriptomic*" OR "transcriptom* sequencing" OR "RNA seq*")) OR (("genomic*" OR "genom* sequencing") AND ("epigenomic*" OR "epigenom* sequencing")) OR (("genomic*" OR "genom* sequencing") AND ("metabolomic*" OR ("metabolo*" AND "mass spectrometry") OR ("metabolo*" AND "NMR"))) OR (("genomic*" OR "genom* sequencing") AND ("proteomic*" OR ("proteo*" AND "mass spectrometry"))) OR (("transcriptomic*" OR "transcriptom* sequencing" OR "RNA seq*") AND ("epigenomic*" OR "epigenom* sequencing")) OR (("transcriptomic*" OR "transcriptom* sequencing" OR "RNA seq*") AND ("metabolomic*" OR ("metabolo*" AND "mass spectrometry") OR ("metabolo*" AND "NMR"))) OR (("transcriptomic*" OR "transcriptom* sequencing" OR "RNA seq*") AND ("proteomic*" OR ("proteo*" AND "mass spectrometry"))) OR (("epigenomic*" OR "epigenom* sequencing") AND ("metabolomic*" OR ("metabolo*" AND "mass spectrometry") OR ("metabolo*" AND "NMR"))) OR	51

(("epigenomic*" OR "epigenom* sequencing") AND ("proteomic*" OR ("proteo*" AND "mass spectrometry"))) OR (("metabolomic*" OR ("metabolo*" AND "mass spectrometry") OR ("metabolo*" AND "NMR")) AND ("proteomic*" OR ("proteo*" AND "mass spectrometry"))) OR (("genomic*" OR "genom* sequencing") AND "phosphoproteomic*") OR (("genomic*" OR "genom* sequencing") AND "epitranscriptomic*") OR (("genomic*" OR "genom* sequencing") AND "lipidomic*") OR (("genomic*" OR "genom* sequencing") AND "glycomic*") OR (("genomic*" OR "genom* sequencing") AND "pharmacogenomic*") OR (("genomic*" OR "genom* sequencing") AND "fluxomic*") OR (("genomic*" OR "genom* sequencing") AND "interactomic*") OR (("transcriptomic*" OR "transcriptom* sequencing" OR "RNA seq*") AND "phosphoproteomic*") OR (("transcriptomic*" OR "transcriptom* sequencing" OR "RNA seq*") AND "epitranscriptomic*") OR (("transcriptomic*" OR "transcriptom* sequencing" OR "RNA seq*") AND "lipidomic*") OR (("transcriptomic*" OR "transcriptom* sequencing" OR "RNA seq*") AND "glycomic*") OR (("transcriptomic*" OR "transcriptom* sequencing" OR "RNA seq*") AND "pharmacogenomic*") OR (("transcriptomic*" OR "transcriptom* sequencing" OR "RNA seq*") AND "fluxomic*") OR (("transcriptomic*" OR "transcriptom* sequencing" OR "RNA seq*") AND "interactomic*") OR (("epigenomic*" OR "epigenom* sequencing") AND "phosphoproteomic*") OR (("epigenomic*" OR "epigenom* sequencing") AND "epitranscriptomic*") OR (("epigenomic*" OR "epigenom* sequencing") AND "lipidomic*") OR (("epigenomic*" OR "epigenom* sequencing") AND "glycomic*") OR (("epigenomic*" OR "epigenom* sequencing") AND "pharmacogenomic*") OR (("epigenomic*" OR "epigenom* sequencing") AND "fluxomic*") OR (("epigenomic*" OR "epigenom* sequencing") AND "interactomic*") OR (("metabolomic*" OR ("metabolo*" AND "mass spectrometry") OR ("metabolo*" AND "NMR")) AND "phosphoproteomic*") OR (("metabolomic*" OR ("metabolo*" AND "mass spectrometry") OR ("metabolo*" AND "NMR")) AND "epitranscriptomic*") OR (("metabolomic*" OR ("metabolo*" AND "mass spectrometry") OR ("metabolo*" AND "NMR")) AND "lipidomic*") OR (("metabolomic*" OR ("metabolo*" AND "mass spectrometry") OR ("metabolo*" AND "NMR")) AND "glycomic*") OR (("metabolomic*" OR ("metabolo*" AND "mass spectrometry") OR ("metabolo*" AND "NMR")) AND "pharmacogenomic*") OR (("metabolomic*" OR ("metabolo*" AND "mass spectrometry") OR ("metabolo*" AND "NMR")) AND "fluxomic*") OR (("metabolomic*" OR ("metabolo*" AND "mass spectrometry") OR ("metabolo*" AND "NMR")) AND "interactomic*") OR (("proteomic*" OR ("proteo*" AND "mass spectrometry")) AND "phosphoproteomic*") OR (("proteomic*" OR ("proteo*" AND "mass spectrometry")) AND "epitranscriptomic*") OR (("proteomic*" OR ("proteo*" AND "mass spectrometry")) AND	

"lipidomic*") OR (("proteomic*" OR ("proteo*" AND "mass spectrometry")) AND "glycomic*") OR (("proteomic*" OR ("proteo*" AND "mass spectrometry")) AND "pharmacogenomic*") OR (("proteomic*" OR ("proteo*" AND "mass spectrometry")) AND "fluxomic*") OR (("proteomic*" OR ("proteo*" AND "mass spectrometry")) AND "interactomic*"))) OR ("multiomic*" OR "multi-omic*" OR "crossomic*" OR "cross-omic*" OR "cross omic*" OR "panomic*" OR "pan-omic*" OR "transomic*" OR "trans-omic*" OR "multiple omic*")) AND ("producer cell line" OR "immortalized cell line" OR "immortalised cell line" OR "stable cell line" OR "cell line development" OR "cell line engineering" OR "suspension culture" OR "adherent culture" OR "defined media" OR "serum-free media" OR "fed-batch" OR "semi-perfusion" OR "perfusion culture" OR "media optimization" OR "media optimisation" OR "media components" OR "bioprocess" OR "bioreactor" OR "upstream processing" OR "downstream processing" OR "process scale-up" OR "scale-up" OR "continuous bioprocessing"))	
TS=((("Human Embryonic Kidney" OR "HEK293*" OR "HEK 293*") AND "cell*" NOT ("Chinese Hamster Ovary" OR "CHO cell*" OR "CHO-K1" OR "CHO-S" OR "CHO-DG44" OR "CHO-DUK" OR "CHOZN" OR "CHO-T" OR "CHO-Lec*" OR "CHO-H" OR "CHO-AT3-2" OR "CHO-C" OR "CHO-M" OR "CHO-AI") AND "cell*") AND (((("genomic*" OR "genom* sequencing") AND ("transcriptomic*" OR "transcriptom* sequencing" OR "RNA seq*")) OR (("genomic*" OR "genom* sequencing") AND ("epigenomic*" OR "epigenom* sequencing")) OR (("genomic*" OR "genom* sequencing") AND ("metabolomic*" OR ("metabolo*" AND "mass spectrometry") OR ("metabolo*" AND "NMR"))) OR (("genomic*" OR "genom* sequencing") AND ("proteomic*" OR ("proteo*" AND "mass spectrometry"))) OR (("transcriptomic*" OR "transcriptom* sequencing" OR "RNA seq*") AND ("epigenomic*" OR "epigenom* sequencing")) OR (("transcriptomic*" OR "transcriptom* sequencing" OR "RNA seq*") AND ("metabolomic*" OR ("metabolo*" AND "mass spectrometry") OR ("metabolo*" AND "NMR"))) OR (("transcriptomic*" OR "transcriptom* sequencing" OR "RNA seq*") AND ("proteomic*" OR ("proteo*" AND "mass spectrometry"))) OR (("epigenomic*" OR "epigenom* sequencing") AND ("metabolomic*" OR ("metabolo*" AND "mass spectrometry") OR ("metabolo*" AND "NMR"))) OR (("epigenomic*" OR "epigenom* sequencing") AND ("proteomic*" OR ("proteo*" AND "mass spectrometry"))) OR (("metabolomic*" OR ("metabolo*" AND "mass spectrometry") OR ("metabolo*" AND "NMR")) AND ("proteomic*" OR ("proteo*" AND "mass spectrometry"))) OR (("genomic*" OR "genom* sequencing") AND "phosphoproteomic*") OR (("genomic*" OR "genom* sequencing") AND "epitranscriptomic*") OR (("genomic*" OR "genom* sequencing") AND "lipidomic*") OR	4

(("genomic*" OR "genom* sequencing") AND "glycomic*") OR (("genomic*" OR "genom* sequencing") AND "pharmacogenomic*") OR (("genomic*" OR "genom* sequencing") AND "fluxomic*") OR (("genomic*" OR "genom* sequencing") AND "interactomic*") OR (("transcriptomic*" OR "transcriptom* sequencing" OR "RNA seq*") AND "phosphoproteomic*") OR (("transcriptomic*" OR "transcriptom* sequencing" OR "RNA seq*") AND "epitranscriptomic*") OR (("transcriptomic*" OR "transcriptom* sequencing" OR "RNA seq*") AND "lipidomic*") OR (("transcriptomic*" OR "transcriptom* sequencing" OR "RNA seq*") AND "glycomic*") OR (("transcriptomic*" OR "transcriptom* sequencing" OR "RNA seq*") AND "pharmacogenomic*") OR (("transcriptomic*" OR "transcriptom* sequencing" OR "RNA seq*") AND "fluxomic*") OR (("transcriptomic*" OR "transcriptom* sequencing" OR "RNA seq*") AND "interactomic*") OR (("epigenomic*" OR "epigenom* sequencing") AND "phosphoproteomic*") OR (("epigenomic*" OR "epigenom* sequencing") AND "epitranscriptomic*") OR (("epigenomic*" OR "epigenom* sequencing") AND "lipidomic*") OR (("epigenomic*" OR "epigenom* sequencing") AND "glycomic*") OR (("epigenomic*" OR "epigenom* sequencing") AND "pharmacogenomic*") OR (("epigenomic*" OR "epigenom* sequencing") AND "fluxomic*") OR (("epigenomic*" OR "epigenom* sequencing") AND "interactomic*") OR (("metabolomic*" OR ("metabolo*" AND "mass spectrometry") OR ("metabolo*" AND "NMR")) AND "phosphoproteomic*") OR (("metabolomic*" OR ("metabolo*" AND "mass spectrometry") OR ("metabolo*" AND "NMR")) AND "epitranscriptomic*") OR (("metabolomic*" OR ("metabolo*" AND "mass spectrometry") OR ("metabolo*" AND "NMR")) AND "lipidomic*") OR (("metabolomic*" OR ("metabolo*" AND "mass spectrometry") OR ("metabolo*" AND "NMR")) AND "glycomic*") OR (("metabolomic*" OR ("metabolo*" AND "mass spectrometry") OR ("metabolo*" AND "NMR")) AND "pharmacogenomic*") OR (("metabolomic*" OR ("metabolo*" AND "mass spectrometry") OR ("metabolo*" AND "NMR")) AND "fluxomic*") OR (("metabolomic*" OR ("metabolo*" AND "mass spectrometry") OR ("metabolo*" AND "NMR")) AND "interactomic*") OR (("proteomic*" OR ("proteo*" AND "mass spectrometry")) AND "phosphoproteomic*") OR (("proteomic*" OR ("proteo*" AND "mass spectrometry")) AND "epitranscriptomic*") OR (("proteomic*" OR ("proteo*" AND "mass spectrometry")) AND "lipidomic*") OR (("proteomic*" OR ("proteo*" AND "mass spectrometry")) AND "glycomic*") OR (("proteomic*" OR ("proteo*" AND "mass spectrometry")) AND "pharmacogenomic*") OR (("proteomic*" OR ("proteo*" AND "mass spectrometry")) AND "fluxomic*") OR (("proteomic*" OR ("proteo*" AND "mass spectrometry")) AND "interactomic*"))) AND ("producer cell line" OR "immortalized cell line" OR "immortalised cell line" OR "stable cell line" OR "cell line	

development" OR "cell line engineering" OR "suspension culture" OR "adherent culture" OR "defined media" OR "serum-free media" OR "fed-batch" OR "semi-perfusion" OR "perfusion culture" OR "media optimization" OR "media optimisation" OR "media components" OR "bioprocess" OR "bioreactor" OR "upstream processing" OR "downstream processing" OR "process scale-up" OR "scale-up" OR "continuous bioprocessing"))	
TS=((("Human Embryonic Kidney" OR "HEK293*" OR "HEK 293*") AND "cell*" NOT ("Chinese Hamster Ovary" OR "CHO cell*" OR "CHO-K1" OR "CHO-S" OR "CHO-DG44" OR "CHO-DUK" OR "CHOZN" OR "CHO-T" OR "CHO-Lec*" OR "CHO-H" OR "CHO-AT3-2" OR "CHO-C" OR "CHO-M" OR "CHO-AI") AND "cell*") AND (((("genomic*" OR "genom* sequencing") AND ("transcriptomic*" OR "transcriptom* sequencing" OR "RNA seq*")) OR (("genomic*" OR "genom* sequencing") AND ("epigenomic*" OR "epigenom* sequencing")) OR (("genomic*" OR "genom* sequencing") AND ("metabolomic*" OR ("metabolo*" AND "mass spectrometry") OR ("metabolo*" AND "NMR"))) OR (("genomic*" OR "genom* sequencing") AND ("proteomic*" OR ("proteo*" AND "mass spectrometry"))) OR (("transcriptomic*" OR "transcriptom* sequencing" OR "RNA seq*") AND ("epigenomic*" OR "epigenom* sequencing")) OR (("transcriptomic*" OR "transcriptom* sequencing" OR "RNA seq*") AND ("metabolomic*" OR ("metabolo*" AND "mass spectrometry") OR ("metabolo*" AND "NMR"))) OR (("transcriptomic*" OR "transcriptom* sequencing" OR "RNA seq*") AND ("proteomic*" OR ("proteo*" AND "mass spectrometry"))) OR (("epigenomic*" OR "epigenom* sequencing") AND ("metabolomic*" OR ("metabolo*" AND "mass spectrometry") OR ("metabolo*" AND "NMR"))) OR (("epigenomic*" OR "epigenom* sequencing") AND ("proteomic*" OR ("proteo*" AND "mass spectrometry"))) OR (("metabolomic*" OR ("metabolo*" AND "mass spectrometry") OR ("metabolo*" AND "NMR")) AND ("proteomic*" OR ("proteo*" AND "mass spectrometry"))) OR (("genomic*" OR "genom* sequencing") AND "phosphoproteomic*") OR (("genomic*" OR "genom* sequencing") AND "epitranscriptomic*") OR (("genomic*" OR "genom* sequencing") AND "lipidomic*") OR (("genomic*" OR "genom* sequencing") AND "glycomic*") OR (("genomic*" OR "genom* sequencing") AND "pharmacogenomic*") OR (("genomic*" OR "genom* sequencing") AND "fluxomic*") OR (("genomic*" OR "genom* sequencing") AND "interactomic*") OR (("transcriptomic*" OR "transcriptom* sequencing" OR "RNA seq*") AND "phosphoproteomic*") OR (("transcriptomic*" OR "transcriptom* sequencing" OR "RNA seq*") AND "epitranscriptomic*") OR (("transcriptomic*" OR "transcriptom* sequencing" OR "RNA seq*") AND "lipidomic*") OR (("transcriptomic*" OR "transcriptom* sequencing" OR "RNA seq*") AND "glycomic*")	3

OR (("transcriptomic*" OR "transcriptom* sequencing" OR "RNA seq*") AND "pharmacogenomic*") OR (("transcriptomic*" OR "transcriptom* sequencing" OR "RNA seq*") AND "fluxomic*") OR (("transcriptomic*" OR "transcriptom* sequencing" OR "RNA seq*") AND "interactomic*") OR (("epigenomic*" OR "epigenom* sequencing") AND "phosphoproteomic*") OR (("epigenomic*" OR "epigenom* sequencing") AND "epitranscriptomic*") OR (("epigenomic*" OR "epigenom* sequencing") AND "lipidomic*") OR (("epigenomic*" OR "epigenom* sequencing") AND "glycomic*") OR (("epigenomic*" OR "epigenom* sequencing") AND "pharmacogenomic*") OR (("epigenomic*" OR "epigenom* sequencing") AND "fluxomic*") OR (("epigenomic*" OR "epigenom* sequencing") AND "interactomic*") OR (("metabolomic*" OR ("metabolo*" AND "mass spectrometry") OR ("metabolo*" AND "NMR")) AND "phosphoproteomic*") OR (("metabolomic*" OR ("metabolo*" AND "mass spectrometry") OR ("metabolo*" AND "NMR")) AND "epitranscriptomic*") OR (("metabolomic*" OR ("metabolo*" AND "mass spectrometry") OR ("metabolo*" AND "NMR")) AND "lipidomic*") OR (("metabolomic*" OR ("metabolo*" AND "mass spectrometry") OR ("metabolo*" AND "NMR")) AND "glycomic*") OR (("metabolomic*" OR ("metabolo*" AND "mass spectrometry") OR ("metabolo*" AND "NMR")) AND "pharmacogenomic*") OR (("metabolomic*" OR ("metabolo*" AND "mass spectrometry") OR ("metabolo*" AND "NMR")) AND "fluxomic*") OR (("metabolomic*" OR ("metabolo*" AND "mass spectrometry") OR ("metabolo*" AND "NMR")) AND "interactomic*") OR (("proteomic*" OR ("proteo*" AND "mass spectrometry")) AND "phosphoproteomic*") OR (("proteomic*" OR ("proteo*" AND "mass spectrometry")) AND "epitranscriptomic*") OR (("proteomic*" OR ("proteo*" AND "mass spectrometry")) AND "lipidomic*") OR (("proteomic*" OR ("proteo*" AND "mass spectrometry")) AND "glycomic*") OR (("proteomic*" OR ("proteo*" AND "mass spectrometry")) AND "pharmacogenomic*") OR (("proteomic*" OR ("proteo*" AND "mass spectrometry")) AND "fluxomic*") OR (("proteomic*" OR ("proteo*" AND "mass spectrometry")) AND "interactomic*"))) AND ("multiomic*" OR "multi-omic*" OR "crossomic*" OR "cross-omic*" OR "cross omic*" OR "panomic*" OR "pan-omic*" OR "transomic*" OR "trans-omic*" OR "multiple omic*") AND ("producer cell line" OR "immortalized cell line" OR "immortalised cell line" OR "stable cell line" OR "cell line development" OR "cell line engineering" OR "suspension culture" OR "adherent culture" OR "defined media" OR "serum-free media" OR "fed-batch" OR "semi-perfusion" OR "perfusion culture" OR "media optimization" OR "media optimisation" OR "media components" OR "bioprocess" OR "bioreactor" OR "upstream processing" OR "downstream processing" OR "process scale-up" OR "scale-up" OR "continuous bioprocessing"))	

TS=((("Human Embryonic Kidney" OR "HEK293*" OR "HEK 293*") AND "cell*" NOT ("Chinese Hamster Ovary" OR "CHO cell*" OR "CHO-K1" OR "CHO-S" OR "CHO-DG44" OR "CHO-DUK" OR "CHOZN" OR "CHO-T" OR "CHO-Lec*" OR "CHO-H" OR "CHO-AT3-2" OR "CHO-C" OR "CHO-M" OR "CHO-AI") AND "cell*") AND ((((("genomic*" OR "genom* sequencing") AND ("transcriptomic*" OR "transcriptom* sequencing" OR "RNA seq*")) OR (("genomic*" OR "genom* sequencing") AND ("epigenomic*" OR "epigenom* sequencing")) OR (("genomic*" OR "genom* sequencing") AND ("metabolomic*" OR ("metabolo*" AND "mass spectrometry") OR ("metabolo*" AND "NMR"))) OR (("genomic*" OR "genom* sequencing") AND ("proteomic*" OR ("proteo*" AND "mass spectrometry"))) OR (("transcriptomic*" OR "transcriptom* sequencing" OR "RNA seq*") AND ("epigenomic*" OR "epigenom* sequencing")) OR (("transcriptomic*" OR "transcriptom* sequencing" OR "RNA seq*") AND ("metabolomic*" OR ("metabolo*" AND "mass spectrometry") OR ("metabolo*" AND "NMR"))) OR (("transcriptomic*" OR "transcriptom* sequencing" OR "RNA seq*") AND ("proteomic*" OR ("proteo*" AND "mass spectrometry"))) OR (("epigenomic*" OR "epigenom* sequencing") AND ("metabolomic*" OR ("metabolo*" AND "mass spectrometry") OR ("metabolo*" AND "NMR"))) OR (("epigenomic*" OR "epigenom* sequencing") AND ("proteomic*" OR ("proteo*" AND "mass spectrometry"))) OR (("metabolomic*" OR ("metabolo*" AND "mass spectrometry") OR ("metabolo*" AND "NMR")) AND ("proteomic*" OR ("proteo*" AND "mass spectrometry"))) OR (("genomic*" OR "genom* sequencing") AND "phosphoproteomic*") OR (("genomic*" OR "genom* sequencing") AND "epitranscriptomic*") OR (("genomic*" OR "genom* sequencing") AND "lipidomic*") OR (("genomic*" OR "genom* sequencing") AND "glycomic*") OR (("genomic*" OR "genom* sequencing") AND "pharmacogenomic*") OR (("genomic*" OR "genom* sequencing") AND "fluxomic*") OR (("genomic*" OR "genom* sequencing") AND "interactomic*") OR (("transcriptomic*" OR "transcriptom* sequencing" OR "RNA seq*") AND "phosphoproteomic*") OR (("transcriptomic*" OR "transcriptom* sequencing" OR "RNA seq*") AND "epitranscriptomic*") OR (("transcriptomic*" OR "transcriptom* sequencing" OR "RNA seq*") AND "lipidomic*") OR (("transcriptomic*" OR "transcriptom* sequencing" OR "RNA seq*") AND "glycomic*") OR (("transcriptomic*" OR "transcriptom* sequencing" OR "RNA seq*") AND "pharmacogenomic*") OR (("transcriptomic*" OR "transcriptom* sequencing" OR "RNA seq*") AND "fluxomic*") OR (("transcriptomic*" OR "transcriptom* sequencing" OR "RNA seq*") AND "interactomic*") OR (("epigenomic*" OR "epigenom* sequencing") AND "phosphoproteomic*") OR (("epigenomic*" OR "epigenom* sequencing") AND "epitranscriptomic*") OR (("epigenomic*" OR "epigenom* sequencing") AND "lipidomic*")	4

OR (("epigenomic*" OR "epigenom* sequencing") AND "glycomic*") OR (("epigenomic*" OR "epigenom* sequencing") AND "pharmacogenomic*") OR (("epigenomic*" OR "epigenom* sequencing") AND "fluxomic*") OR (("epigenomic*" OR "epigenom* sequencing") AND "interactomic*") OR (("metabolomic*" OR ("metabolo*" AND "mass spectrometry") OR ("metabolo*" AND "NMR")) AND "phosphoproteomic*") OR (("metabolomic*" OR ("metabolo*" AND "mass spectrometry") OR ("metabolo*" AND "NMR")) AND "epitranscriptomic*") OR (("metabolomic*" OR ("metabolo*" AND "mass spectrometry") OR ("metabolo*" AND "NMR")) AND "lipidomic*") OR (("metabolomic*" OR ("metabolo*" AND "mass spectrometry") OR ("metabolo*" AND "NMR")) AND "glycomic*") OR (("metabolomic*" OR ("metabolo*" AND "mass spectrometry") OR ("metabolo*" AND "NMR")) AND "pharmacogenomic*") OR (("metabolomic*" OR ("metabolo*" AND "mass spectrometry") OR ("metabolo*" AND "NMR")) AND "fluxomic*") OR (("metabolomic*" OR ("metabolo*" AND "mass spectrometry") OR ("metabolo*" AND "NMR")) AND "interactomic*") OR (("proteomic*" OR ("proteo*" AND "mass spectrometry")) AND "phosphoproteomic*") OR (("proteomic*" OR ("proteo*" AND "mass spectrometry")) AND "epitranscriptomic*") OR (("proteomic*" OR ("proteo*" AND "mass spectrometry")) AND "lipidomic*") OR (("proteomic*" OR ("proteo*" AND "mass spectrometry")) AND "glycomic*") OR (("proteomic*" OR ("proteo*" AND "mass spectrometry")) AND "pharmacogenomic*") OR (("proteomic*" OR ("proteo*" AND "mass spectrometry")) AND "fluxomic*") OR (("proteomic*" OR ("proteo*" AND "mass spectrometry")) AND "interactomic*"))) OR ("multiomic*" OR "multi-omic*" OR "crossomic*" OR "cross-omic*" OR "cross omic*" OR "panomic*" OR "pan-omic*" OR "transomic*" OR "trans-omic*" OR "multiple omic*")) AND ("producer cell line" OR "immortalized cell line" OR "immortalised cell line" OR "stable cell line" OR "cell line development" OR "cell line engineering" OR "suspension culture" OR "adherent culture" OR "defined media" OR "serum-free media" OR "fed-batch" OR "semi-perfusion" OR "perfusion culture" OR "media optimization" OR "media optimisation" OR "media components" OR "bioprocess" OR "bioreactor" OR "upstream processing" OR "downstream processing" OR "process scale-up" OR "scale-up" OR "continuous bioprocessing"))	
TS=((("Chinese Hamster Ovary" OR "CHO cell*" OR "CHO-K1" OR "CHO-S" OR "CHO-DG44" OR "CHO-DUK" OR "CHOZN" OR "CHO-T" OR "CHO-Lec*" OR "CHO-H" OR "CHO-AT3-2" OR "CHO-C" OR "CHO-M" OR "CHO-AI") AND "cell*" AND ("Human Embryonic Kidney" OR "HEK293*" OR "HEK 293*") AND "cell*") AND (((("genomic*" OR "genom* sequencing") AND ("transcriptomic*" OR "transcriptom* sequencing" OR "RNA seq*")) OR (("genomic*" OR "genom* sequencing") AND	1

("epigenomic*" OR "epigenom* sequencing")) OR (("genomic*" OR "genom* sequencing") AND ("metabolomic*" OR ("metabolo*" AND "mass spectrometry") OR ("metabolo*" AND "NMR"))) OR (("genomic*" OR "genom* sequencing") AND ("proteomic*" OR ("proteo*" AND "mass spectrometry"))) OR (("transcriptomic*" OR "transcriptom* sequencing" OR "RNA seq*") AND ("epigenomic*" OR "epigenom* sequencing")) OR (("transcriptomic*" OR "transcriptom* sequencing" OR "RNA seq*") AND ("metabolomic*" OR ("metabolo*" AND "mass spectrometry") OR ("metabolo*" AND "NMR"))) OR (("transcriptomic*" OR "transcriptom* sequencing" OR "RNA seq*") AND ("proteomic*" OR ("proteo*" AND "mass spectrometry"))) OR (("epigenomic*" OR "epigenom* sequencing") AND ("metabolomic*" OR ("metabolo*" AND "mass spectrometry") OR ("metabolo*" AND "NMR"))) OR (("epigenomic*" OR "epigenom* sequencing") AND ("proteomic*" OR ("proteo*" AND "mass spectrometry"))) OR (("metabolomic*" OR ("metabolo*" AND "mass spectrometry") OR ("metabolo*" AND "NMR")) AND ("proteomic*" OR ("proteo*" AND "mass spectrometry"))) OR (("genomic*" OR "genom* sequencing") AND "phosphoproteomic*") OR (("genomic*" OR "genom* sequencing") AND "epitranscriptomic*") OR (("genomic*" OR "genom* sequencing") AND "lipidomic*") OR (("genomic*" OR "genom* sequencing") AND "glycomic*") OR (("genomic*" OR "genom* sequencing") AND "pharmacogenomic*") OR (("genomic*" OR "genom* sequencing") AND "fluxomic*") OR (("genomic*" OR "genom* sequencing") AND "interactomic*") OR (("transcriptomic*" OR "transcriptom* sequencing" OR "RNA seq*") AND "phosphoproteomic*") OR (("transcriptomic*" OR "transcriptom* sequencing" OR "RNA seq*") AND "epitranscriptomic*") OR (("transcriptomic*" OR "transcriptom* sequencing" OR "RNA seq*") AND "lipidomic*") OR (("transcriptomic*" OR "transcriptom* sequencing" OR "RNA seq*") AND "glycomic*") OR (("transcriptomic*" OR "transcriptom* sequencing" OR "RNA seq*") AND "pharmacogenomic*") OR (("transcriptomic*" OR "transcriptom* sequencing" OR "RNA seq*") AND "fluxomic*") OR (("transcriptomic*" OR "transcriptom* sequencing" OR "RNA seq*") AND "interactomic*") OR (("epigenomic*" OR "epigenom* sequencing") AND "phosphoproteomic*") OR (("epigenomic*" OR "epigenom* sequencing") AND "epitranscriptomic*") OR (("epigenomic*" OR "epigenom* sequencing") AND "lipidomic*") OR (("epigenomic*" OR "epigenom* sequencing") AND "glycomic*") OR (("epigenomic*" OR "epigenom* sequencing") AND "pharmacogenomic*") OR (("epigenomic*" OR "epigenom* sequencing") AND "fluxomic*") OR (("epigenomic*" OR "epigenom* sequencing") AND "interactomic*") OR (("metabolomic*" OR ("metabolo*" AND "mass spectrometry") OR ("metabolo*" AND "NMR")) AND "phosphoproteomic*") OR (("metabolomic*" OR ("metabolo*" AND "mass spectrometry")	

OR ("metabolo*" AND "NMR")) AND "epitranscriptomic*") OR (("metabolomic*" OR ("metabolo*" AND "mass spectrometry") OR ("metabolo*" AND "NMR")) AND "lipidomic*") OR (("metabolomic*" OR ("metabolo*" AND "mass spectrometry") OR ("metabolo*" AND "NMR")) AND "glycomic*") OR (("metabolomic*" OR ("metabolo*" AND "mass spectrometry") OR ("metabolo*" AND "NMR")) AND "pharmacogenomic*") OR (("metabolomic*" OR ("metabolo*" AND "mass spectrometry") OR ("metabolo*" AND "NMR")) AND "fluxomic*") OR (("metabolomic*" OR ("metabolo*" AND "mass spectrometry") OR ("metabolo*" AND "NMR")) AND "interactomic*") OR (("proteomic*" OR ("proteo*" AND "mass spectrometry")) AND "phosphoproteomic*") OR (("proteomic*" OR ("proteo*" AND "mass spectrometry")) AND "epitranscriptomic*") OR (("proteomic*" OR ("proteo*" AND "mass spectrometry")) AND "lipidomic*") OR (("proteomic*" OR ("proteo*" AND "mass spectrometry")) AND "glycomic*") OR (("proteomic*" OR ("proteo*" AND "mass spectrometry")) AND "pharmacogenomic*") OR (("proteomic*" OR ("proteo*" AND "mass spectrometry")) AND "fluxomic*") OR (("proteomic*" OR ("proteo*" AND "mass spectrometry")) AND "interactomic*"))) AND ("producer cell line" OR "immortalized cell line" OR "immortalised cell line" OR "stable cell line" OR "cell line development" OR "cell line engineering" OR "suspension culture" OR "adherent culture" OR "defined media" OR "serum-free media" OR "fed-batch" OR "semi-perfusion" OR "perfusion culture" OR "media optimization" OR "media optimisation" OR "media components" OR "bioprocess" OR "bioreactor" OR "upstream processing" OR "downstream processing" OR "process scale-up" OR "scale-up" OR "continuous bioprocessing"))	
TS=((("Chinese Hamster Ovary" OR "CHO cell*" OR "CHO-K1" OR "CHO-S" OR "CHO-DG44" OR "CHO-DUK" OR "CHOZN" OR "CHO-T" OR "CHO-Lec*" OR "CHO-H" OR "CHO-AT3-2" OR "CHO-C" OR "CHO-M" OR "CHO-AI") AND "cell*" AND ("Human Embryonic Kidney" OR "HEK293*" OR "HEK 293*") AND "cell*") AND (((("genomic*" OR "genom* sequencing") AND ("transcriptomic*" OR "transcriptom* sequencing" OR "RNA seq*")) OR (("genomic*" OR "genom* sequencing") AND ("epigenomic*" OR "epigenom* sequencing")) OR (("genomic*" OR "genom* sequencing") AND ("metabolomic*" OR ("metabolo*" AND "mass spectrometry") OR ("metabolo*" AND "NMR"))) OR (("genomic*" OR "genom* sequencing") AND ("proteomic*" OR ("proteo*" AND "mass spectrometry"))) OR (("transcriptomic*" OR "transcriptom* sequencing" OR "RNA seq*") AND ("epigenomic*" OR "epigenom* sequencing")) OR (("transcriptomic*" OR "transcriptom* sequencing" OR "RNA seq*") AND ("metabolomic*" OR ("metabolo*" AND "mass spectrometry") OR ("metabolo*" AND "NMR"))) OR (("transcriptomic*" OR "transcriptom* sequencing" OR "RNA	0

seq*") AND ("proteomic*" OR ("proteo*" AND "mass spectrometry"))) OR (("epigenomic*" OR "epigenom* sequencing") AND ("metabolomic*" OR ("metabolo*" AND "mass spectrometry") OR ("metabolo*" AND "NMR"))) OR (("epigenomic*" OR "epigenom* sequencing") AND ("proteomic*" OR ("proteo*" AND "mass spectrometry"))) OR (("metabolomic*" OR ("metabolo*" AND "mass spectrometry") OR ("metabolo*" AND "NMR")) AND ("proteomic*" OR ("proteo*" AND "mass spectrometry"))) OR (("genomic*" OR "genom* sequencing") AND "phosphoproteomic*") OR (("genomic*" OR "genom* sequencing") AND "epitranscriptomic*") OR (("genomic*" OR "genom* sequencing") AND "lipidomic*") OR (("genomic*" OR "genom* sequencing") AND "glycomic*") OR (("genomic*" OR "genom* sequencing") AND "pharmacogenomic*") OR (("genomic*" OR "genom* sequencing") AND "fluxomic*") OR (("genomic*" OR "genom* sequencing") AND "interactomic*") OR (("transcriptomic*" OR "transcriptom* sequencing" OR "RNA seq*") AND "phosphoproteomic*") OR (("transcriptomic*" OR "transcriptom* sequencing" OR "RNA seq*") AND "epitranscriptomic*") OR (("transcriptomic*" OR "transcriptom* sequencing" OR "RNA seq*") AND "lipidomic*") OR (("transcriptomic*" OR "transcriptom* sequencing" OR "RNA seq*") AND "glycomic*") OR (("transcriptomic*" OR "transcriptom* sequencing" OR "RNA seq*") AND "pharmacogenomic*") OR (("transcriptomic*" OR "transcriptom* sequencing" OR "RNA seq*") AND "fluxomic*") OR (("transcriptomic*" OR "transcriptom* sequencing" OR "RNA seq*") AND "interactomic*") OR (("epigenomic*" OR "epigenom* sequencing") AND "phosphoproteomic*") OR (("epigenomic*" OR "epigenom* sequencing") AND "epitranscriptomic*") OR (("epigenomic*" OR "epigenom* sequencing") AND "lipidomic*") OR (("epigenomic*" OR "epigenom* sequencing") AND "glycomic*") OR (("epigenomic*" OR "epigenom* sequencing") AND "pharmacogenomic*") OR (("epigenomic*" OR "epigenom* sequencing") AND "fluxomic*") OR (("epigenomic*" OR "epigenom* sequencing") AND "interactomic*") OR (("metabolomic*" OR ("metabolo*" AND "mass spectrometry") OR ("metabolo*" AND "NMR")) AND "phosphoproteomic*") OR (("metabolomic*" OR ("metabolo*" AND "mass spectrometry") OR ("metabolo*" AND "NMR")) AND "epitranscriptomic*") OR (("metabolomic*" OR ("metabolo*" AND "mass spectrometry") OR ("metabolo*" AND "NMR")) AND "lipidomic*") OR (("metabolomic*" OR ("metabolo*" AND "mass spectrometry") OR ("metabolo*" AND "NMR")) AND "glycomic*") OR (("metabolomic*" OR ("metabolo*" AND "mass spectrometry") OR ("metabolo*" AND "NMR")) AND "pharmacogenomic*") OR (("metabolomic*" OR ("metabolo*" AND "mass spectrometry") OR ("metabolo*" AND "NMR")) AND "fluxomic*") OR (("metabolomic*" OR ("metabolo*" AND "mass spectrometry") OR ("metabolo*" AND "NMR")) AND "interactomic*") OR	

(("proteomic*" OR ("proteo*" AND "mass spectrometry")) AND "phosphoproteomic*") OR (("proteomic*" OR ("proteo*" AND "mass spectrometry")) AND "epitranscriptomic*") OR (("proteomic*" OR ("proteo*" AND "mass spectrometry")) AND "lipidomic*") OR (("proteomic*" OR ("proteo*" AND "mass spectrometry")) AND "glycomic*") OR (("proteomic*" OR ("proteo*" AND "mass spectrometry")) AND "pharmacogenomic*") OR (("proteomic*" OR ("proteo*" AND "mass spectrometry")) AND "fluxomic*") OR (("proteomic*" OR ("proteo*" AND "mass spectrometry")) AND "interactomic*"))) AND ("multiomic*" OR "multi-omic*" OR "crossomic*" OR "cross-omic*" OR "cross omic*" OR "panomic*" OR "pan-omic*" OR "transomic*" OR "trans-omic*" OR "multiple omic*") AND ("producer cell line" OR "immortalized cell line" OR "immortalised cell line" OR "stable cell line" OR "cell line development" OR "cell line engineering" OR "suspension culture" OR "adherent culture" OR "defined media" OR "serum-free media" OR "fed-batch" OR "semi-perfusion" OR "perfusion culture" OR "media optimization" OR "media optimisation" OR "media components" OR "bioprocess" OR "bioreactor" OR "upstream processing" OR "downstream processing" OR "process scale-up" OR "scale-up" OR "continuous bioprocessing"))	
TS=((("Chinese Hamster Ovary" OR "CHO cell*" OR "CHO-K1" OR "CHO-S" OR "CHO-DG44" OR "CHO-DUK" OR "CHOZN" OR "CHO-T" OR "CHO-Lec*" OR "CHO-H" OR "CHO-AT3-2" OR "CHO-C" OR "CHO-M" OR "CHO-AI") AND "cell*" AND ("Human Embryonic Kidney" OR "HEK293*" OR "HEK 293*") AND "cell*") AND (((((("genomic*" OR "genom* sequencing") AND ("transcriptomic*" OR "transcriptom* sequencing" OR "RNA seq*")) OR (("genomic*" OR "genom* sequencing") AND ("epigenomic*" OR "epigenom* sequencing")) OR (("genomic*" OR "genom* sequencing") AND ("metabolomic*" OR ("metabolo*" AND "mass spectrometry") OR ("metabolo*" AND "NMR"))) OR (("genomic*" OR "genom* sequencing") AND ("proteomic*" OR ("proteo*" AND "mass spectrometry"))) OR (("transcriptomic*" OR "transcriptom* sequencing" OR "RNA seq*") AND ("epigenomic*" OR "epigenom* sequencing")) OR (("transcriptomic*" OR "transcriptom* sequencing" OR "RNA seq*") AND ("metabolomic*" OR ("metabolo*" AND "mass spectrometry") OR ("metabolo*" AND "NMR"))) OR (("transcriptomic*" OR "transcriptom* sequencing" OR "RNA seq*") AND ("proteomic*" OR ("proteo*" AND "mass spectrometry"))) OR (("epigenomic*" OR "epigenom* sequencing") AND ("metabolomic*" OR ("metabolo*" AND "mass spectrometry") OR ("metabolo*" AND "NMR"))) OR (("epigenomic*" OR "epigenom* sequencing") AND ("proteomic*" OR ("proteo*" AND "mass spectrometry"))) OR (("metabolomic*" OR ("metabolo*" AND "mass spectrometry") OR ("metabolo*" AND "NMR")) AND ("proteomic*" OR ("proteo*"	2

AND "mass spectrometry"))) OR (("genomic*" OR "genom* sequencing") AND "phosphoproteomic*") OR (("genomic*" OR "genom* sequencing") AND "epitranscriptomic*") OR (("genomic*" OR "genom* sequencing") AND "lipidomic*") OR (("genomic*" OR "genom* sequencing") AND "glycomic*") OR (("genomic*" OR "genom* sequencing") AND "pharmacogenomic*") OR (("genomic*" OR "genom* sequencing") AND "fluxomic*") OR (("genomic*" OR "genom* sequencing") AND "interactomic*") OR (("transcriptomic*" OR "transcriptom* sequencing" OR "RNA seq*") AND "phosphoproteomic*") OR (("transcriptomic*" OR "transcriptom* sequencing" OR "RNA seq*") AND "epitranscriptomic*") OR (("transcriptomic*" OR "transcriptom* sequencing" OR "RNA seq*") AND "lipidomic*") OR (("transcriptomic*" OR "transcriptom* sequencing" OR "RNA seq*") AND "glycomic*") OR (("transcriptomic*" OR "transcriptom* sequencing" OR "RNA seq*") AND "pharmacogenomic*") OR (("transcriptomic*" OR "transcriptom* sequencing" OR "RNA seq*") AND "fluxomic*") OR (("transcriptomic*" OR "transcriptom* sequencing" OR "RNA seq*") AND "interactomic*") OR (("epigenomic*" OR "epigenom* sequencing") AND "phosphoproteomic*") OR (("epigenomic*" OR "epigenom* sequencing") AND "epitranscriptomic*") OR (("epigenomic*" OR "epigenom* sequencing") AND "lipidomic*") OR (("epigenomic*" OR "epigenom* sequencing") AND "glycomic*") OR (("epigenomic*" OR "epigenom* sequencing") AND "pharmacogenomic*") OR (("epigenomic*" OR "epigenom* sequencing") AND "fluxomic*") OR (("epigenomic*" OR "epigenom* sequencing") AND "interactomic*") OR (("metabolomic*" OR ("metabolo*" AND "mass spectrometry") OR ("metabolo*" AND "NMR")) AND "phosphoproteomic*") OR (("metabolomic*" OR ("metabolo*" AND "mass spectrometry") OR ("metabolo*" AND "NMR")) AND "epitranscriptomic*") OR (("metabolomic*" OR ("metabolo*" AND "mass spectrometry") OR ("metabolo*" AND "NMR")) AND "lipidomic*") OR (("metabolomic*" OR ("metabolo*" AND "mass spectrometry") OR ("metabolo*" AND "NMR")) AND "glycomic*") OR (("metabolomic*" OR ("metabolo*" AND "mass spectrometry") OR ("metabolo*" AND "NMR")) AND "pharmacogenomic*") OR (("metabolomic*" OR ("metabolo*" AND "mass spectrometry") OR ("metabolo*" AND "NMR")) AND "fluxomic*") OR (("metabolomic*" OR ("metabolo*" AND "mass spectrometry") OR ("metabolo*" AND "NMR")) AND "interactomic*") OR (("proteomic*" OR ("proteo*" AND "mass spectrometry")) AND "phosphoproteomic*") OR (("proteomic*" OR ("proteo*" AND "mass spectrometry")) AND "epitranscriptomic*") OR (("proteomic*" OR ("proteo*" AND "mass spectrometry")) AND "lipidomic*") OR (("proteomic*" OR ("proteo*" AND "mass spectrometry")) AND "glycomic*") OR (("proteomic*" OR ("proteo*" AND "mass spectrometry")) AND "pharmacogenomic*") OR (("proteomic*" OR ("proteo*" AND	

"mass spectrometry")) AND "fluxomic*") OR (("proteomic*" OR ("proteo*" AND "mass spectrometry")) AND "interactomic*"))) OR ("multiomic*" OR "multi-omic*" OR "crossomic*" OR "cross-omic*" OR "cross omic*" OR "panomic*" OR "pan-omic*" OR "transomic*" OR "trans-omic*" OR "multiple omic*")) AND ("producer cell line" OR "immortalized cell line" OR "immortalised cell line" OR "stable cell line" OR "cell line development" OR "cell line engineering" OR "suspension culture" OR "adherent culture" OR "defined media" OR "serum-free media" OR "fed-batch" OR "semi-perfusion" OR "perfusion culture" OR "media optimization" OR "media optimisation" OR "media components" OR "bioprocess" OR "bioreactor" OR "upstream processing" OR "downstream processing" OR "process scale-up" OR "scale-up" OR "continuous bioprocessing"))	

Supplementary Note S4

To validate our results across databases, we adapted our final search query for studies which contained both cell lines, at least two omics term or multi-omics terms and bioprocessing terms and applied it to Scopus and PubMed (*Table S15).* Scopus retrieved more studies(n=3) compared to Web of Science (n=2), while PubMed as expected returned no results (n=0). Interestingly no publications were common to both WoS and Scopus highlighting the importance of choice when selecting an appropriate database source for literature searches.

Table S15 Final Search query adapted for Scopus and PubMed

**Scopus Search Term**	**Number of Studies**
(((TITLE-ABS-KEY(((("Chinese Hamster Ovary" OR "CHO cell*" OR "CHO-K1" OR "CHO-S" OR "CHO-DG44" OR "CHO-DUK" OR "CHOZN" OR "CHO-T" OR "CHO-Lec*" OR "CHO-H" OR "CHO-AT3-2" OR "CHO-C" OR "CHO-M" OR "CHO-AI") AND "cell*" AND ("Human Embryonic Kidney" OR "HEK293*" OR "HEK 293*") AND "cell*") AND ((((("genomic*" OR "genom* sequencing") AND ("transcriptomic*" OR "transcriptom* sequencing" OR "RNA seq*")) OR (("genomic*" OR "genom* sequencing") AND ("epigenomic*" OR "epigenom* sequencing")) OR (("genomic*" OR "genom* sequencing") AND ("metabolomic*" OR ("metabolo*" AND "mass spectrometry") OR ("metabolo*" AND "NMR"))) OR (("genomic*" OR "genom* sequencing") AND ("proteomic*" OR ("proteo*" AND "mass spectrometry"))) OR (("transcriptomic*" OR "transcriptom* sequencing" OR "RNA seq*") AND ("epigenomic*" OR "epigenom* sequencing")) OR (("transcriptomic*" OR "transcriptom* sequencing" OR "RNA seq*") AND ("metabolomic*" OR ("metabolo*" AND "mass spectrometry") OR ("metabolo*" AND "NMR"))) OR (("transcriptomic*" OR "transcriptom* sequencing" OR "RNA seq*") AND ("proteomic*" OR ("proteo*" AND "mass	3

spectrometry"))) OR (("epigenomic*" OR "epigenom* sequencing") AND ("metabolomic*" OR ("metabolo*" AND "mass spectrometry") OR ("metabolo*" AND "NMR"))) OR (("epigenomic*" OR "epigenom* sequencing") AND ("proteomic*" OR ("proteo*" AND "mass spectrometry"))) OR (("metabolomic*" OR ("metabolo*" AND "mass spectrometry") OR ("metabolo*" AND "NMR")) AND ("proteomic*" OR ("proteo*" AND "mass spectrometry"))) OR (("genomic*" OR "genom* sequencing") AND "phosphoproteomic*") OR (("genomic*" OR "genom* sequencing") AND "epitranscriptomic*") OR (("genomic*" OR "genom* sequencing") AND "lipidomic*") OR (("genomic*" OR "genom* sequencing") AND "glycomic*") OR (("genomic*" OR "genom* sequencing") AND "pharmacogenomic*") OR (("genomic*" OR "genom* sequencing") AND "fluxomic*") OR (("genomic*" OR "genom* sequencing") AND "interactomic*") OR (("transcriptomic*" OR "transcriptom* sequencing" OR "RNA seq*") AND "phosphoproteomic*") OR (("transcriptomic*" OR "transcriptom* sequencing" OR "RNA seq*") AND "epitranscriptomic*") OR (("transcriptomic*" OR "transcriptom* sequencing" OR "RNA seq*") AND "lipidomic*") OR (("transcriptomic*" OR "transcriptom* sequencing" OR "RNA seq*") AND "glycomic*") OR (("transcriptomic*" OR "transcriptom* sequencing" OR "RNA seq*") AND "pharmacogenomic*") OR (("transcriptomic*" OR "transcriptom* sequencing" OR "RNA seq*") AND "fluxomic*") OR (("transcriptomic*" OR "transcriptom* sequencing" OR "RNA seq*") AND "interactomic*") OR (("epigenomic*" OR "epigenom* sequencing") AND "phosphoproteomic*") OR (("epigenomic*" OR "epigenom* sequencing") AND "epitranscriptomic*") OR (("epigenomic*" OR "epigenom* sequencing") AND "lipidomic*") OR (("epigenomic*" OR "epigenom* sequencing") AND "glycomic*") OR (("epigenomic*" OR "epigenom* sequencing") AND "pharmacogenomic*") OR (("epigenomic*" OR "epigenom* sequencing") AND "fluxomic*") OR (("epigenomic*" OR "epigenom* sequencing") AND "interactomic*") OR (("metabolomic*" OR ("metabolo*" AND "mass spectrometry") OR ("metabolo*" AND "NMR")) AND "phosphoproteomic*") OR (("metabolomic*" OR ("metabolo*" AND "mass spectrometry") OR ("metabolo*" AND "NMR")) AND "epitranscriptomic*") OR (("metabolomic*" OR ("metabolo*" AND "mass spectrometry") OR ("metabolo*" AND "NMR")) AND "lipidomic*") OR (("metabolomic*" OR ("metabolo*" AND "mass spectrometry") OR ("metabolo*" AND "NMR")) AND "glycomic*") OR (("metabolomic*" OR ("metabolo*" AND "mass spectrometry") OR ("metabolo*" AND "NMR")) AND "pharmacogenomic*") OR (("metabolomic*" OR ("metabolo*" AND "mass spectrometry") OR ("metabolo*" AND "NMR")) AND "fluxomic*") OR (("metabolomic*" OR ("metabolo*" AND	

"mass spectrometry") OR ("metabolo*" AND "NMR")) AND "interactomic*") OR (("proteomic*" OR ("proteo*" AND "mass spectrometry")) AND "phosphoproteomic*") OR (("proteomic*" OR ("proteo*" AND "mass spectrometry")) AND "epitranscriptomic*") OR (("proteomic*" OR ("proteo*" AND "mass spectrometry")) AND "lipidomic*") OR (("proteomic*" OR ("proteo*" AND "mass spectrometry")) AND "glycomic*") OR (("proteomic*" OR ("proteo*" AND "mass spectrometry")) AND "pharmacogenomic*") OR (("proteomic*" OR ("proteo*" AND "mass spectrometry")) AND "fluxomic*") OR (("proteomic*" OR ("proteo*" AND "mass spectrometry")) AND "interactomic*"))) OR ("multiomic*" OR "multi-omic*" OR "crossomic*" OR "cross-omic*" OR "cross omic*" OR "panomic*" OR "pan-omic*" OR "transomic*" OR "trans-omic*" OR "multiple omic*")) AND ("producer cell line" OR "immortalized cell line" OR "immortalised cell line" OR "stable cell line" OR "cell line development" OR "cell line engineering" OR "suspension culture" OR "adherent culture" OR "defined media" OR "serum-free media" OR "fed-batch" OR "semi-perfusion" OR "perfusion culture" OR "media optimization" OR "media optimisation" OR "media components" OR "bioprocess" OR "bioreactor" OR "upstream processing" OR "downstream processing" OR "process scale-up" OR "scale-up" OR "continuous bioprocessing"))))))	
**Pubmed Search term**	
(("Chinese Hamster Ovary"[Title/Abstract] OR "CHO cell*"[Title/Abstract] OR "CHO-K1"[Title/Abstract] OR "CHO-S"[Title/Abstract] OR "CHO-DG44"[Title/Abstract] OR "CHO-DUK"[Title/Abstract] OR "CHOZN"[Title/Abstract] OR "CHO-T"[Title/Abstract] OR "CHO-Lec*"[Title/Abstract] OR "CHO-H"[Title/Abstract] OR "CHO-AT3-2"[Title/Abstract] OR "CHO-C"[Title/Abstract] OR "CHO-M"[Title/Abstract] OR "CHO-AI"[Title/Abstract]) AND "cell*"[Title/Abstract] AND ("Human Embryonic Kidney"[Title/Abstract] OR "HEK293*"[Title/Abstract] OR "HEK 293*"[Title/Abstract]) AND "cell*"[Title/Abstract]) AND ((((("genomic*"[Title/Abstract] OR "genom* sequencing"[Title/Abstract]) AND ("transcriptomic*"[Title/Abstract] OR "transcriptom* sequencing"[Title/Abstract] OR "RNA seq*"[Title/Abstract])) OR (("genomic*"[Title/Abstract] OR "genom* sequencing"[Title/Abstract]) AND ("epigenomic*"[Title/Abstract] OR "epigenom* sequencing"[Title/Abstract])) OR (("genomic*"[Title/Abstract] OR "genom* sequencing"[Title/Abstract]) AND ("metabolomic*"[Title/Abstract] OR ("metabolo*"[Title/Abstract] AND "mass spectrometry"[Title/Abstract]) OR ("metabolo*"[Title/Abstract]	0

AND "NMR"[Title/Abstract]))) OR (("genomic*"[Title/Abstract] OR "genom* sequencing"[Title/Abstract]) AND ("proteomic*"[Title/Abstract] OR ("proteo*"[Title/Abstract] AND "mass spectrometry"[Title/Abstract]))) OR (("transcriptomic*"[Title/Abstract] OR "transcriptom* sequencing"[Title/Abstract] OR "RNA seq*"[Title/Abstract]) AND ("epigenomic*"[Title/Abstract] OR "epigenom* sequencing"[Title/Abstract])) OR (("transcriptomic*"[Title/Abstract] OR "transcriptom* sequencing"[Title/Abstract] OR "RNA seq*"[Title/Abstract]) AND ("metabolomic*"[Title/Abstract] OR ("metabolo*"[Title/Abstract] AND "mass spectrometry"[Title/Abstract]) OR ("metabolo*"[Title/Abstract] AND "NMR"[Title/Abstract]))) OR (("transcriptomic*"[Title/Abstract] OR "transcriptom* sequencing"[Title/Abstract] OR "RNA seq*"[Title/Abstract]) AND ("proteomic*"[Title/Abstract] OR ("proteo*"[Title/Abstract] AND "mass spectrometry"[Title/Abstract]))) OR (("epigenomic*"[Title/Abstract] OR "epigenom* sequencing"[Title/Abstract]) AND ("metabolomic*"[Title/Abstract] OR ("metabolo*"[Title/Abstract] AND "mass spectrometry"[Title/Abstract]) OR ("metabolo*"[Title/Abstract] AND "NMR"[Title/Abstract]))) OR (("epigenomic*"[Title/Abstract] OR "epigenom* sequencing"[Title/Abstract]) AND ("proteomic*"[Title/Abstract] OR ("proteo*"[Title/Abstract] AND "mass spectrometry"[Title/Abstract]))) OR (("metabolomic*"[Title/Abstract] OR ("metabolo*"[Title/Abstract] AND "mass spectrometry"[Title/Abstract]) OR ("metabolo*"[Title/Abstract] AND "NMR"[Title/Abstract])) AND ("proteomic*"[Title/Abstract] OR ("proteo*"[Title/Abstract] AND "mass spectrometry"[Title/Abstract]))) OR (("genomic*"[Title/Abstract] OR "genom* sequencing"[Title/Abstract]) AND "phosphoproteomic*"[Title/Abstract]) OR (("genomic*"[Title/Abstract] OR "genom* sequencing"[Title/Abstract]) AND "epitranscriptomic*"[Title/Abstract]) OR (("genomic*"[Title/Abstract] OR "genom* sequencing"[Title/Abstract]) AND "lipidomic*"[Title/Abstract]) OR (("genomic*"[Title/Abstract] OR "genom* sequencing"[Title/Abstract]) AND "glycomic*"[Title/Abstract]) OR (("genomic*"[Title/Abstract] OR "genom* sequencing"[Title/Abstract]) AND "pharmacogenomic*"[Title/Abstract]) OR (("genomic*"[Title/Abstract] OR "genom* sequencing"[Title/Abstract]) AND "fluxomic*"[Title/Abstract])	

OR (("genomic*"[Title/Abstract] OR "genom* sequencing"[Title/Abstract]) AND "interactomic*"[Title/Abstract]) OR (("transcriptomic*"[Title/Abstract] OR "transcriptom* sequencing"[Title/Abstract] OR "RNA seq*"[Title/Abstract]) AND "phosphoproteomic*"[Title/Abstract]) OR (("transcriptomic*"[Title/Abstract] OR "transcriptom* sequencing"[Title/Abstract] OR "RNA seq*"[Title/Abstract]) AND "epitranscriptomic*"[Title/Abstract]) OR (("transcriptomic*"[Title/Abstract] OR "transcriptom* sequencing"[Title/Abstract] OR "RNA seq*"[Title/Abstract]) AND "lipidomic*"[Title/Abstract]) OR (("transcriptomic*"[Title/Abstract] OR "transcriptom* sequencing"[Title/Abstract] OR "RNA seq*"[Title/Abstract]) AND "glycomic*"[Title/Abstract]) OR (("transcriptomic*"[Title/Abstract] OR "transcriptom* sequencing"[Title/Abstract] OR "RNA seq*"[Title/Abstract]) AND "pharmacogenomic*"[Title/Abstract]) OR (("transcriptomic*"[Title/Abstract] OR "transcriptom* sequencing"[Title/Abstract] OR "RNA seq*"[Title/Abstract]) AND "fluxomic*"[Title/Abstract]) OR (("transcriptomic*"[Title/Abstract] OR "transcriptom* sequencing"[Title/Abstract] OR "RNA seq*"[Title/Abstract]) AND "interactomic*"[Title/Abstract]) OR (("epigenomic*"[Title/Abstract] OR "epigenom* sequencing"[Title/Abstract]) AND "phosphoproteomic*"[Title/Abstract]) OR (("epigenomic*"[Title/Abstract] OR "epigenom* sequencing"[Title/Abstract]) AND "epitranscriptomic*"[Title/Abstract]) OR (("epigenomic*"[Title/Abstract] OR "epigenom* sequencing"[Title/Abstract]) AND "lipidomic*"[Title/Abstract]) OR (("epigenomic*"[Title/Abstract] OR "epigenom* sequencing"[Title/Abstract]) AND "glycomic*"[Title/Abstract]) OR (("epigenomic*"[Title/Abstract] OR "epigenom* sequencing"[Title/Abstract]) AND "pharmacogenomic*"[Title/Abstract]) OR (("epigenomic*"[Title/Abstract] OR "epigenom* sequencing"[Title/Abstract]) AND "fluxomic*"[Title/Abstract]) OR (("epigenomic*"[Title/Abstract] OR "epigenom* sequencing"[Title/Abstract]) AND "interactomic*"[Title/Abstract]) OR (("metabolomic*"[Title/Abstract] OR ("metabolo*"[Title/Abstract] AND "mass spectrometry"[Title/Abstract]) OR ("metabolo*"[Title/Abstract] AND "NMR"[Title/Abstract])) AND "phosphoproteomic*"[Title/Abstract]) OR (("metabolomic*"[Title/Abstract] OR ("metabolo*"[Title/Abstract] AND "mass spectrometry"[Title/Abstract]) OR ("metabolo*"[Title/Abstract]	

AND "NMR"[Title/Abstract])) AND "epitranscriptomic*"[Title/Abstract]) OR (("metabolomic*"[Title/Abstract] OR ("metabolo*"[Title/Abstract] AND "mass spectrometry"[Title/Abstract]) OR ("metabolo*"[Title/Abstract] AND "NMR"[Title/Abstract])) AND "lipidomic*"[Title/Abstract]) OR (("metabolomic*"[Title/Abstract] OR ("metabolo*"[Title/Abstract] AND "mass spectrometry"[Title/Abstract]) OR ("metabolo*"[Title/Abstract] AND "NMR"[Title/Abstract])) AND "glycomic*"[Title/Abstract]) OR (("metabolomic*"[Title/Abstract] OR ("metabolo*"[Title/Abstract] AND "mass spectrometry"[Title/Abstract]) OR ("metabolo*"[Title/Abstract] AND "NMR"[Title/Abstract])) AND "pharmacogenomic*"[Title/Abstract]) OR (("metabolomic*"[Title/Abstract] OR ("metabolo*"[Title/Abstract] AND "mass spectrometry"[Title/Abstract]) OR ("metabolo*"[Title/Abstract] AND "NMR"[Title/Abstract])) AND "fluxomic*"[Title/Abstract]) OR (("metabolomic*"[Title/Abstract] OR ("metabolo*"[Title/Abstract] AND "mass spectrometry"[Title/Abstract]) OR ("metabolo*"[Title/Abstract] AND "NMR"[Title/Abstract])) AND "interactomic*"[Title/Abstract]) OR (("proteomic*"[Title/Abstract] OR ("proteo*"[Title/Abstract] AND "mass spectrometry"[Title/Abstract])) AND "phosphoproteomic*"[Title/Abstract]) OR (("proteomic*"[Title/Abstract] OR ("proteo*"[Title/Abstract] AND "mass spectrometry"[Title/Abstract])) AND "epitranscriptomic*"[Title/Abstract]) OR (("proteomic*"[Title/Abstract] OR ("proteo*"[Title/Abstract] AND "mass spectrometry"[Title/Abstract])) AND "lipidomic*"[Title/Abstract]) OR (("proteomic*"[Title/Abstract] OR ("proteo*"[Title/Abstract] AND "mass spectrometry"[Title/Abstract])) AND "glycomic*"[Title/Abstract]) OR (("proteomic*"[Title/Abstract] OR ("proteo*"[Title/Abstract] AND "mass spectrometry"[Title/Abstract])) AND "pharmacogenomic*"[Title/Abstract]) OR (("proteomic*"[Title/Abstract] OR ("proteo*"[Title/Abstract] AND "mass spectrometry"[Title/Abstract])) AND "fluxomic*"[Title/Abstract]) OR (("proteomic*"[Title/Abstract] OR ("proteo*"[Title/Abstract] AND "mass spectrometry"[Title/Abstract])) AND "interactomic*"[Title/Abstract]))) OR ("multiomic*"[Title/Abstract] OR "multi-omic*"[Title/Abstract] OR "crossomic*"[Title/Abstract] OR "cross-omic*"[Title/Abstract] OR "cross omic*"[Title/Abstract] OR "panomic*"[Title/Abstract] OR "pan-omic*"[Title/Abstract] OR "transomic*"[Title/Abstract] OR "trans-omic*"[Title/Abstract]	

OR "multiple omic*"[Title/Abstract])) AND ("producer cell line"[Title/Abstract] OR "immortalized cell line"[Title/Abstract] OR "immortalised cell line"[Title/Abstract] OR "stable cell line"[Title/Abstract] OR "cell line development"[Title/Abstract] OR "cell line engineering"[Title/Abstract] OR "suspension culture"[Title/Abstract] OR "adherent culture"[Title/Abstract] OR "defined media"[Title/Abstract] OR "serum-free media"[Title/Abstract] OR "fed-batch"[Title/Abstract] OR "semi-perfusion"[Title/Abstract] OR "perfusion culture"[Title/Abstract] OR "media optimization"[Title/Abstract] OR "media optimisation"[Title/Abstract] OR "media components"[Title/Abstract] OR "bioprocess"[Title/Abstract] OR "bioreactor"[Title/Abstract] OR "upstream processing"[Title/Abstract] OR "downstream processing"[Title/Abstract] OR "process scale-up"[Title/Abstract] OR "scale-up"[Title/Abstract] OR "continuous bioprocessing"[Title/Abstract])	

References

1 Tang, F. *et al.* mRNA-Seq whole-transcriptome analysis of a single cell. *Nature Methods* **6**, 377-382 (2009). https://doi.org:10.1038/nmeth.1315

2 Method of the Year 2013. *Nature Methods* **11**, 1-1 (2014). https://doi.org:10.1038/nmeth.2801

3 Smallwood, S. A. *et al.* Single-cell genome-wide bisulfite sequencing for assessing epigenetic heterogeneity. *Nat Methods* **11**, 817-820 (2014). https://doi.org:10.1038/nmeth.3035

4 Buenrostro, J. D. *et al.* Single-cell chromatin accessibility reveals principles of regulatory variation. *Nature* **523**, 486-490 (2015). https://doi.org:10.1038/nature14590

5 DataM Intelligence 4 Market Research LLP. Single Cell Omics Market Size, Share, Industry Growth Report. (openPR.com, 2025).

PRISMA 2020 Checklist

Section and Topic	Item #	Checklist item	Location where item is reported
**TITLE**			
Title	1	Identify the report as a systematic review.	Page 1
**ABSTRACT**			
Abstract	2	See the PRISMA 2020 for Abstracts checklist.	Page 2
**INTRODUCTION**			
Rationale	3	Describe the rationale for the review in the context of existing knowledge.	Page 2+3
Objectives	4	Provide an explicit statement of the objective(s) or question(s) the review addresses.	Page 4
**METHODS**			
Eligibility criteria	5	Specify the inclusion and exclusion criteria for the review and how studies were grouped for the syntheses.	Page 27
Information sources	6	Specify all databases, registers, websites, organisations, reference lists and other sources searched or consulted to identify studies. Specify the date when each source was last searched or consulted.	Page 26
Search strategy	7	Present the full search strategies for all databases, registers and websites, including any filters and limits used.	Page 27 + Supplementary
Selection process	8	Specify the methods used to decide whether a study met the inclusion criteria of the review, including how many reviewers screened each record and each report retrieved, whether they worked independently, and if applicable, details of automation tools used in the process.	Page 27
Data collection process	9	Specify the methods used to collect data from reports, including how many reviewers collected data from each report, whether they worked independently, any processes for obtaining or confirming data from study investigators, and if applicable, details of automation tools used in the process.	Page 28
Data items	10a	List and define all outcomes for which data were sought. Specify whether all results that were compatible with each outcome domain in each study were sought (e.g. for all measures, time points, analyses), and if not, the methods used to decide which results to collect.	Page 28-27
	10b	List and define all other variables for which data were sought (e.g. participant and intervention characteristics, funding sources). Describe any assumptions made about any missing or unclear information.	Page 15 + 19
Study risk of bias assessment	11	Specify the methods used to assess risk of bias in the included studies, including details of the tool(s) used, how many reviewers assessed each study and whether they worked independently, and if applicable, details of automation tools used in the process.	Page 17
Effect measures	12	Specify for each outcome the effect measure(s) (e.g. risk ratio, mean difference) used in the synthesis or presentation of results.	Page 23
Synthesis methods	13a	Describe the processes used to decide which studies were eligible for each synthesis (e.g. tabulating the study intervention characteristics and comparing against the planned groups for each synthesis (item #5)).	Page 14 +19
	13b	Describe any methods required to prepare the data for presentation or synthesis, such as handling of missing summary statistics, or data conversions.	Page 19 + Supplementary Code
	13c	Describe any methods used to tabulate or visually display results of individual studies and syntheses.	Supplementary Code
	13d	Describe any methods used to synthesize results and provide a rationale for the choice(s). If meta-analysis was performed, describe the model(s), method(s) to identify the presence and extent of statistical heterogeneity, and software package(s) used.	Page 27-28 + Supplementary Code
	13e	Describe any methods used to explore possible causes of heterogeneity among study results (e.g. subgroup analysis, meta-regression).	Page 23
	13f	Describe any sensitivity analyses conducted to assess robustness of the synthesized results.	Page 28

PRISMA

PRISMA 2020 Checklist

Section and Topic	Item #	Checklist item	Location where item is reported
Reporting bias assessment	14	Describe any methods used to assess risk of bias due to missing results in a synthesis (arising from reporting biases).	Page 17
Certainty assessment	15	Describe any methods used to assess certainty (or confidence) in the body of evidence for an outcome.	Page 28
**RESULTS**			
Study selection	16a	Describe the results of the search and selection process, from the number of records identified in the search to the number of studies included in the review, ideally using a flow diagram.	Pages 5, 7-15, + Supplementary Materials
	16b	Cite studies that might appear to meet the inclusion criteria, but which were excluded, and explain why they were excluded.	Page 19
Study characteristics	17	Cite each included study and present its characteristics.	Pages 19-23
Risk of bias in studies	18	Present assessments of risk of bias for each included study.	Page 19
Results of individual studies	19	For all outcomes, present, for each study: (a) summary statistics for each group (where appropriate) and (b) an effect estimate and its precision (e.g. confidence/credible interval), ideally using structured tables or plots.	Page 25
Results of syntheses	20a	For each synthesis, briefly summarise the characteristics and risk of bias among contributing studies.	Page 15 + 19
	20b	Present results of all statistical syntheses conducted. If meta-analysis was done, present for each the summary estimate and its precision (e.g. confidence/credible interval) and measures of statistical heterogeneity. If comparing groups, describe the direction of the effect.	Page 15
	20c	Present results of all investigations of possible causes of heterogeneity among study results.	Page 15-19
	20d	Present results of all sensitivity analyses conducted to assess the robustness of the synthesized results.	Page 15-19
Reporting biases	21	Present assessments of risk of bias due to missing results (arising from reporting biases) for each synthesis assessed.	Page 4, 10, 17 +26
Certainty of evidence	22	Present assessments of certainty (or confidence) in the body of evidence for each outcome assessed.	Page 25
**DISCUSSION**			
Discussion	23a	Provide a general interpretation of the results in the context of other evidence.	Page 23
	23b	Discuss any limitations of the evidence included in the review.	Page 15 + 23
	23c	Discuss any limitations of the review processes used.	Page 15
	23d	Discuss implications of the results for practice, policy, and future research.	Page 24 +25
**OTHER INFORMATION**			
Registration and protocol	24a	Provide registration information for the review, including register name and registration number, or state that the review was not registered.	N/A
	24b	Indicate where the review protocol can be accessed, or state that a protocol was not prepared.	N/A
	24c	Describe and explain any amendments to information provided at registration or in the protocol.	N/A
Support	25	Describe sources of financial or non-financial support for the review, and the role of the funders or sponsors in the review.	Page 29
Competing	26	Declare any competing interests of review authors.	Page 29

PRISMA 2020 Checklist

Section and Topic	Item #	Checklist item	Location where item is reported
interests			
Availability of data, code and other materials	27	Report which of the following are publicly available and where they can be found: template data collection forms; data extracted from included studies; data used for all analyses; analytic code; any other materials used in the review.	Page 29

From: Page MJ, McKenzie JE, Bossuyt PM, Boutron I, Hoffmann TC, Mulrow CD, et al. The PRISMA 2020 statement: an updated guideline for reporting systematic reviews. BMJ 2021;372:n71. doi: 10.1136/bmj.n71.
This work is licensed under CC BY 4.0. To view a copy of this license, visit https://creativecommons.org/licenses/by/4.0/
